# Dynamic Field Theory of Executive Function: Identifying Early Neurocognitive Markers

**DOI:** 10.1111/mono.12478

**Published:** 2024-12-04

**Authors:** Alexis McCraw, Jacqueline Sullivan, Kara Lowery, Rachel Eddings, Hollis R. Heim, Aaron T. Buss

**Affiliations:** ^1^ Department of Psychology University of Tennessee Knoxville

## Abstract

In this Monograph, we explored neurocognitive predictors of executive function (EF) development in a cohort of children followed longitudinally from 30 to 54 months of age. We tested predictions of a dynamic field model that explains development in a benchmark measure of EF development, the dimensional change card sort (DCCS) task. This is a rule‐use task that measures children's ability to switch between sorting cards by shape or color rules. A key developmental mechanism in the model is that dimensional label learning drives EF development.

Data collection began in February 2019 and was completed in April 2022 on the Knoxville campus of the University of Tennessee. Our cohort included 20 children (13 female) all of whom were White (not Hispanic/Latinx) from an urban area in southern United States, and the sample annual family income distribution ranged from low to high (most families falling between $40,000 and 59,000 per year (note that we address issues of generalizability and the small sample size throughout the monograph)). We tested the influence of dimensional label learning on DCCS performance by longitudinally assessing neurocognitive function across multiple domains at 30 and 54 months of age. We measured dimensional label learning with comprehension and production tasks for shape and color labels. Simple EF was measured with the Simon task which required children to respond to images of a cat or dog with a lateralized (left/right) button press. Response conflict was manipulated in this task based on the spatial location of the stimulus which could be neutral (central), congruent, or incongruent with the spatial lateralization of the response. Dimensional understanding was measured with an object matching task requiring children to generalize similarity between objects that matched within the dimensions of color or shape. We first identified neural measures associated with performance and development on each of these tasks. We then examined which of these measures predicted performance on the DCCS task at 54 months. We measured neural activity with functional near‐infrared spectroscopy across bilateral frontal, temporal, and parietal cortices.

Our results identified an array of neurocognitive mechanisms associated with development within each domain we assessed. Importantly, our results suggest that dimensional label learning impacts the development of EF. Neural activation in left frontal cortex during dimensional label production at 30 months of age predicted EF performance at 54 months of age. We discussed these results in the context of efforts to train EF with broad transfer. We also discussed a new autonomy‐centered EF framework. The dynamic field model on which we have motivated the current research makes decisions autonomously and various factors can influence the types of decisions that the model makes. In this way, EF is a property of neurocognitive dynamics, which can be influenced by individual factors and contextual effects. We also discuss how this conceptual framework can generalize beyond the specific example of dimensional label learning and DCCS performance to other aspects of EF and how this framework can help to understand how EF unfolds in unique individual, cultural, and contextual factors.

Measures of EF during early childhood are associated with a wide range of development outcomes, including academic skills and quality of life. The hope is that broad aspects of development can be improved by implementing interventions aimed at facilitating EF development. However, this promise has been largely unrealized. Previous work on EF development has been limited by a focus on EF components, such as inhibition, working memory, and switching. Similarly, intervention research has focused on practicing EF tasks that target these specific components of EF. While performance typically improves on the practiced task, improvement rarely generalizes to other EF tasks or other developmental outcomes. The current work is unique because we looked beyond EF itself to identify the lower‐level learning processes that predict EF development. Indeed, the results of this study identify the first learning mechanism involved in the development of EF.

Although the work here provides new targets for interventions in future work, there are also important limitations. First, our sample is not representative of the underlying population of children in the United States under the age of 5. This is a problem in much of the existing developmental cognitive neuroscience research. We discussed challenges to the generalizability of our findings to the population at large. This is particularly important given that our theory is largely contextual, suggesting that children's unique experiences with learning labels for visual dimensions will impact EF development. Second, we identified a learning mechanism to target in future intervention research; however, it is not clear whether such interventions would benefit all children or how to identify children who would benefit most from such interventions. We also discuss prospective lines of research that can address these limitations, such as targeting families that are typically underrepresented in research, expanding longitudinal studies to examine longer term outcomes such as school‐readiness and academic skills, and using the dynamic field (DF) model to systematically explore how exposure to objects and labels can optimize the neural representations underlying dimensional label learning. Future work remains to understand how such learning processes come to define the contextually and culturally specific skills that emerge over development and how these skills lay the foundation for broad developmental trajectories.

## I Theoretical Issues in the Development of Executive Function (EF)

### Introduction: Defining the Problem of EF Development

One of the most striking aspects of early cognitive development is the emergence of children's ability to control and regulate their behavior in a goal‐directed manner (Blair et al., 2005). These abilities are typically attributed to a set of skills that are collectively referred to as EF. EF is needed in a wide range of situations, such as when a context has a salient stimulus that is not goal appropriate. For example, a classroom contains stimuli associated with an array of behaviors. Sometimes those behaviors need to be inhibited, such as playing with a friend during quiet story time. Sometimes different behaviors should be engaged in different contexts, such as playing with toys during free time, but also putting those toys away at the end of free time. EF abilities are also crucial when a prepotent behavior is no longer appropriate and a new behavior needs to be selected—such as when the child is playing red light/green light and must stop running toward their peer when “red light” is shouted—or when multiple behaviors are potentially relevant but only one is appropriate for currently represented goals—for example when selecting which marker to use to color a tree. Yet in other times, children need to think flexibly about the nature of objects they interact with. For example, they may need to switch from using a marker to color a tree to using that same marker to roll out some Play‐Doh. In this case, the child is focusing on the color of the object in one context but the shape of the object in another context.

Across these examples, children structure their behaviors (e.g., playing with a friend or sitting quietly) and their cognitive processes (e.g., using attention to select specific aspects of the environment that they are remembering or representing) to accommodate goal directed behavior. In the laboratory setting, children can perform simple skills such as inhibiting an immediately desired behavior (e.g., eating a snack) as early as age 2. By age 5, children can typically display more complex abilities such as alternating between different behavioral rules (e.g., sort cards by shape or color; Carlson, 2005). Much research has been directed at uncovering not just the different types of EF skills that children develop, but also how those skills impact broad development outcomes, such as quality‐of‐life and academic skills. For instance, measures of EF during early childhood are associated with health, wealth, and criminal offending outcomes three and four decades later (Moffitt et al., 2011; Richmond‐Rakerd et al., 2021). Moreover, lab‐based measures of EF are also associated with socio‐emotional competencies (Raver et al., 2011) and better predict math and literacy achievement than IQ (Ahmed et al., 2021, 2019; Blair et al., 2005; Bull et al., 2008; Cortés Pascual et al., 2019; Lee et al., 2012; Robson et al., 2020; Santana et al., 2022; Spiegel et al., 2021; St Clair‐Thompson & Gathercole, 2006; Swanson et al., 2008). Thus, EF skills are important for how individuals make decisions that can support long‐term goals and how receptive children are to learning environments. For these reasons, EF is a common target in intervention studies aimed at improving developmental trajectories across a wide range of outcomes.

Many aspects of children's experiences are known to influence EF, such as socioeconomic status (SES) and culture. That is, higher SES is typically associated with better EF (Hackman et al., 2015). Although this effect has been replicated cross‐culturally (Fernald et al., 2011), different rates of EF development across cultures have also been documented. For example, EF was shown to develop more rapidly in a sample of Hong Kong adolescents relative to their counterparts in the United Kingdom, but adult levels of EF did not differ between cultures (Ellefson et al., 2017). Other research has demonstrated cultural differences in the relative impact of SES on EF development. In one study, measures of early childhood EF were compared across the SES gradient between a low‐ to middle‐income country (South Africa) and a high‐income country (Australia). Although EF increased across SES quintiles within samples from each country, participants from the lowest SES quintiles in the low‐ to middle‐income country (South Africa) outperformed individuals from the highest quintile in the high‐income country (Australia; Howard et al., 2020). While these findings illustrate that the gradient effect of SES on EF generalizes across cultures, it also highlights that the behaviors and structures across cultures can influence EF development in unique ways. Thus, it is also important to consider how structure of child care, social interactions, values, and norms within different cultures (e.g., Madhavan & Gross, 2013) can foster the formation of EF processes. Thus, income levels within a country or culture are not the only factor influencing developmental outcomes related to EF. Rather, EF can be impacted by the interactive influence of culture and SES.

Other factors associated with differences in EF performance include race, ethnicity, or cultural background. All typically developing children will get better on EF tasks with age, but these age‐related improvements are stronger in White children than in African American children (Assari, 2020). African American and Hispanic children often enter Kindergarten with lower scores on EF tests (Little, 2017), though these students typically catch up to their peers as school progresses, suggesting that early education experiences do help close the gaps between these students. However, EF tests show racial/ethnic disparities even into adulthood (Rea‐Sandin et al., 2021). Such results should be interpreted with caution, however. The nature of these differences are likely due to differences in children's structured experiences or opportunities for learning across these sociocultural factors. In general, there have historically been economic, racial, and ethnic disparities in educational opportunity and resources, beginning in early childhood (Fram & Kim, 2012). However, it is also important to consider the nature of EF measures. In many ways, EF enables competent and healthy behavior in cultural activities. Across measures of cognitive function, not just EF, scales are typically developed and tested on primarily White and middle‐class children. Indeed, it has recently been suggested that we need to take a different approach to assessing EF that is sensitive to the unique ways that participating in organized behaviors varies cross‐culturally (Gaskins & Alcalá, 2023). We return to discussion of this critical issue in Chapter VIII.

SES and cultural influences are high dimensional and complex, which presents challenges for understanding the exact role of experience on EF development. Nevertheless, these findings demonstrate that EF development is a malleable process. Our goal in this monograph is to provide evidence for how children's early neurocognitive development lays the foundation for later EF development so that more specific aspects of children's experiences can be targeted in future intervention work.

#### Guide for Reading This Monograph and Overview of Chapters

In this first chapter, we describe the current prevailing theories of EF development and highlight the limitations of these theories to identify mechanisms of EF that can be used to improve broad developmental outcomes. Briefly, the primary challenge facing theories of EF development is to strike a balance between generality and specificity. EF inherently operates at a general level: EFs should be able to regulate behavior across a range of contexts and behaviors. For example, inhibiting the behavior of playing with a friend during story time at school likely relies upon similar skills used to stay seated at the table during dinner time. However, EF needs to also be grounded in children's experiences so that we can begin to understand how learning impacts EF development and how EF skills are connected to the specific details of each context.

In Chapter II we describe EF through the lens of dynamic field (DF) theory (Schoner et al., 2016), a computational approach that uses neural population dynamics to explain cognition, behavior, and neural function. In this chapter we provide technical details of the modeling framework and step through examples illustrating the computational properties of the model. However, we also include a Conceptual Summary of the model at the end that is accessible to a more general audience. Briefly, neural population dynamics refers simply to the way that groups of neurons interact with each other to create behavior. The DF model can address the primary challenges of explaining EF development because neural populations in the model are tuned to dimensions of the perception‐action system. Previous work (Buss & Kerr‐German, 2019; Buss & Spencer, 2014, 2018) has demonstrated that this framework can explain a wide range of behavioral and neural data on the development of EF and makes predictions that have been supported by empirical data. In the DF model, EF is a property of neural population dynamics rather than a structural component of the cognitive system. The key insight provided by this framework is that dimensional label‐learning (e.g., forming associations between labels such as “red” and “color” with the visual feature dimension of color) fosters object‐based attention skills that can explain developmental improvement on key measures of EF. In the DF model, forming associations between labels and visual dimensions builds neural connections that can be used to enhance processing of visual feature information that is represented as goal relevant. In this way, the model illustrates how label learning can help children make sense of their perception/action systems and to guide the processing of information to achieve goals in a flexible manner across diverse contexts.

Chapters III through VII describe a longitudinal study that assessed the neurocognitive function across a broad range of cognitive domains between 30 and 54 months of age. Note that Chapter III provides the technical details of the study, including data processing and analyses, in addition to the details of the sample of children included in the study. Chapters IV through VI each include an opening paragraph describing the content of the chapter and a conclusion section which can stand alone to provide a summary of the primary information contained in each chapter.

The goal of the current study was to identify which measures of neurocognitive function are predictive of EF at 54 months of age. Specifically, we tested the central prediction of the DF model that label learning will impact the development of EF. Thus, we measured neurocognitive function across the domains of dimensional label comprehension and production (Chapter IV), dimensional understanding and categorization (Chapter V), and simple EF tasks (Chapter VI) at 30 months. We then identified the neurocognitive measures that were predictive of EF at 54 months of age (Chapter VII). To preview, our results suggest that dimensional label learning is the best predictor of future EF development and changes in the neural systems involved in dimensional label production account for individual variability in a benchmark measure of EF skills. Moreover, the predictive nature of these measures generalizes across the contexts of both complex and simple measures of EF. In Chapter VIII we summarize the overall results of our study and discuss these findings within the broader context of EF development.

### Prevailing Theories of EF

EF was first identified in neuropsychological research involving patients with damage to the frontal cortex. Although these individuals had relatively preserved cognitive functions, they had severe difficulty regulating these cognitive functions or controlling impulses (Duncan, 1986; Lhermitte et al., 1972; Luria, 1973). At its early conception, EF was characterized as a singular function which guided behavior to achieve goal‐directed behavior. Various influential theories proliferated that incorporated such a central executive to explain a wide range of skilled behavior (Atkinson & Shiffrin, 1968; Baddeley & Hitch, 1974; Norman & Shallice, 1986). These views, however, were noted for their limitation in explaining EF because control processes were carried out by an unspecified “central executive” or “control homunculus” that was aware of task goals and the means to achieve them. Thus, these views described the types of functions that fell under the scope of EF and when such functions were needed but did not explain how they were actually carried out.

More recent approaches to EF have attempted to “fractionate” the control system (Monsell & Driver, 2000) by identifying smaller EF components or mechanisms that are involved in goal‐directed behavior. The most common components include, but are not limited to, inhibitory control, working memory, and cognitive switching. *Inhibitory control* is defined as the ability to suppress behaviors and cognitive processes that are irrelevant or inappropriate for currently represented goals; *working memory* is defined as the capacity to manipulate or update actively represented information that can be used to guide future behaviors and decisions; and *cognitive switching* is defined as the ability to update ongoing cognitive processes and representations when goals or contexts change. The various forms of controlled and goal‐directed behavior that are observed in both lab and real‐world settings are thought to arise through the use and combination of such EF components.

This component‐based view of EF is supported by research using factor‐analysis approaches that identify associations between measures of performance on tasks that involve EF. Work with young adults and children has shown that measures of EF load onto factors that can be mapped onto concepts such as inhibition, working memory, or switching. In a seminal study of young adults, Miyake and colleagues (2000) administered a battery of nine “simple” EF tasks that were designed to measure specific components of EF (three tasks each targeting inhibition, working memory, or switching). These tasks have task demands that require only a single EF component. For example, hearing a list of numbers and repeating them requires only the EF component of working memory. Additional tasks were also administered that were designed to be “complex” measures of EF that required more elaborate cognitive processing (e.g., the tower of Hanoi and the Wisconsin card sort). These tasks have additional task demands beyond the engagement of a single EF component. For example, the Wisconsin card sort requires participants to hold in mind categorical information about the sorting cards, learn through trial and error which sorting rule to use, and inhibit the urge to sort based on rules from previous trials. Factor analyses showed that the tasks targeting specific EF components loaded onto common factors (e.g., the three tasks targeting inhibitory control uniquely loaded onto a single factor together). Moreover, these factors also predicted performance on “complex” EF tasks. For example, the tower of Hanoi task was predicted by the latent factor corresponding to the inhibition component and the Wisconsin card sort task was predicted by the factor corresponding to the switching component. Interestingly, not all of the complex tasks were predicted by a latent factor, suggesting that the sampling of tasks in this study did not comprehensively assess all possible EF components. These initial findings, however, gave traction to the idea that EF can be parcellated into smaller component processes.

Developmental research has applied similar factor analysis approaches to demonstrate that children's performance on EF tasks show similar associations to those that parcellate EF with adult participants. Moreover, the complexity of children's EF increases over development, such that measures of children's EF initially load onto a single factor and gradually differentiate to load onto two and eventually the three‐factor structure seen in young adulthood (Agostino et al., 2010; Fuhs & Day, 2011; Huizinga et al., 2006; Lee et al., 2013; Lehto et al., 2003; McAuley & White, 2011; Rose et al., 2011; Shing et al., 2010; van der Sluis et al., 2007; Van der Ven et al., 2012; Wiebe et al., 2008; Willoughby et al., 2012; Wu et al., 2011). It is also important to note that this latent variable approach has yielded inconsistent results regarding the differentiation of EF components over development, suggesting that the structure revealed by this statistical approach depends upon the tasks being administered and the dependent variables that are measured from those tasks (Miller et al., 2012).

The structural component view is appealing because it decomposes complex behavior into simpler functions. Moreover, these components provide a way of explaining domain‐general skills that create the capacity to control behavior beyond the details of a particular task (Zelazo & Carlson, 2023). Generally, this view aligns with findings that specific and overlapping neural processes underlie a wide variety of tasks designed to measure EF ability. That is, EF tasks often vary in terms of specific task content, yet they activate similar frontotemporal regions that presumably support an overarching EF system that is defined independently of the processes and domains over which control is being exerted. This is because these domain‐specific abilities are needed in many other non‐EF tasks and yet do not activate the frontotemporal regions in the way that EF tasks do.

#### Criticisms of the Component View

There are, however, many criticisms against characterizing EF in terms of components. One is that these constructs are largely defined by the tasks used to assess them. Thus, these theoretical constructs do not go beyond simply describing the processing required by the task. This is particularly problematic in developmental work in which the developmental explanation is conflated with the cognitive description. For example, it is theoretically circular to say improvement on an inhibitory control task is due to growth in the inhibitory control component (for discussion see Morton, 2010; Munakata et al., 2003). Second, EF components are conceptualized as domain‐general functions that are defined independently of the informational content in a task or context. Conceptualized in this way, EF components can be used to explain behavior across contexts, but this conceptualization leads to issues when considering the mechanisms that create developmental changes. Specifically, the abstract nature of EF components presents challenges when considering how these components can interface with the details of contexts in which control must be exerted. By drawing such a sharp distinction between the EF component and the information being regulated, there is little opportunity for children's learning or experiences to impact the quality of EF. Indeed, the prevailing theories of EF development propose that maturational processes give rise to growth in EF skills with little consideration of the role of learning (Bunge & Zelazo, 2006; Fiske & Holmboe, 2019; Moriguchi & Hiraki, 2009). Thus, the generalizability of EF components comes at the expense of being able to consider how EF can be built from children's experiences and opportunities for learning.

The component view of EF is also challenged by empirical data on interventions aimed at improving EF. Because it is well‐established that EF is an essential skill set and measures of EF during early childhood are predictive of future success (e.g., academic achievement), identifying targets for intervention that can yield broad transfer is a research priority. These efforts have focused training on tasks that engage specific EF components such as practicing an inhibitory control task; however, these efforts have not led to broad generalization. For example, lab‐based interventions aimed at building EF have shown improvements on the targeted EF domain (e.g., inhibitory control abilities), but not other EFs (e.g., working memory, cognitive switching) (Enge et al., 2014; Morrison & Chein, 2011). Moreover, these domain‐specific improvements often do not generalize to the real‐world behavior or different lab‐based tasks (Aksayli et al., 2019; De Simoni & von Bastian, 2018; Friese et al., 2017; Gobet & Sala, 2023; Jacob & Parkinson, 2015; Kassai et al., 2019; Melby‐Lervåg & Hulme, 2013; Melby‐Lervåg et al., 2016; Nesbitt & Farran, 2021; Rapport et al., 2013; Sala & Gobet, 2019; Shipstead, Hicks, et al., 2012; Shipstead, Redick, et al., 2012; Simons et al., 2016; Takacs & Kassai, 2019). These results challenge the notion that EF is composed of these structural components and motivate a reconceptualization of EF. Our strategy in the current work is to look outside the domains that are typically considered EF to determine the role that dimensional label learning plays in cultivating EF skills.

### Measuring EF Development With The Dimensional Change Card Sort (DCCS) Task

EF follows a protracted developmental time course. The time between ages 2 and 5 is particularly important, however, as this period sees the emergence and refinement of many new EF skills. A wide variety of tasks have been developed to assess different aspects of EF in the lab (e.g., Carlson, 2005); however, one task that has emerged as a benchmark measure of the developmental status of EF is the DCCS task (Zelazo et al., 2003). This task involves using two separate feature dimensions—color and shape—to sort objects. Participants are first instructed to sort by one feature dimension (the pre‐switch dimension) for a series of trials and then to switch to sort by the other dimension (the post‐switch dimension). This task has garnered attention in the literature due to the dramatic shift in performance over development. Most 3‐year‐olds will fail to switch rules despite continuous reminders that the rules have changed whereas most 4‐year‐olds will have little difficulty switching rules (Zelazo et al., 2003). Thus, between the ages of 3 and 5, children's EF undergoes critical developmental change which confers the ability to switch rules in this task.

**Figure 1:**
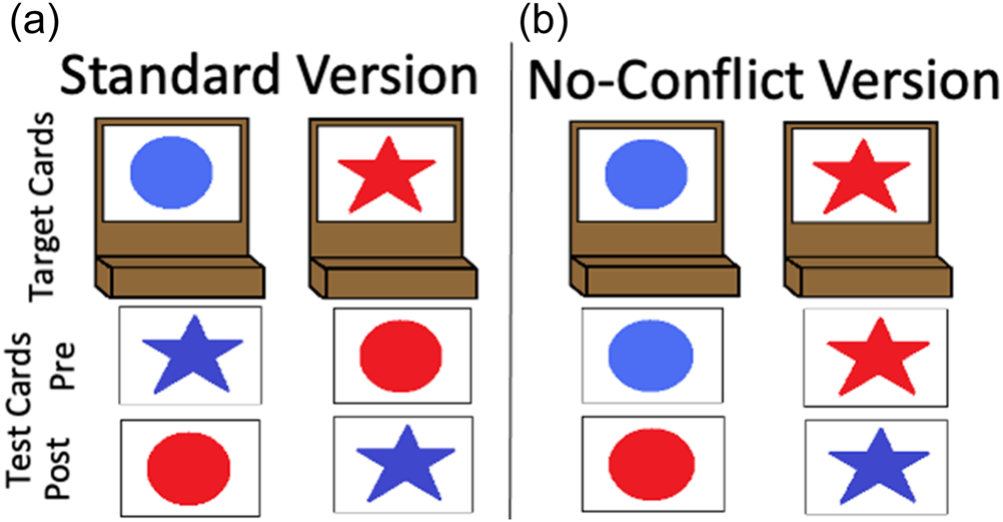
Example of stimuli used in the standard version of the dimensional change card sort task (a) and the no‐conflict version (b).

The DCCS task can be characterized as having relatively straightforward demands on rule‐switching because children are directly instructed on which rules to use for sorting. This contrasts with other tasks that either involve multiple sequences of rule‐switch and rule‐repeat trials (Rogers & Monsell, 1995) or tasks that require participants to infer rule switches based on feedback (Chelune & Baer, 1986). Consideration of the task details, however, illustrates the complex nature of cognitive operations required to switch rules. First, the DCCS task uses target cards that are affixed to the location where children sort cards. These target cards provide visual structure that show which features should be sorted to which location for the shape and color rules, for example, a blue circle and a red star (see Figure 1a). Second, the test cards that children sort match both target cards along different dimensions, for example, a blue star and a red circle (see Figure 1a). Thus, children must selectively attend to the relevant dimension and inhibit attention to the irrelevant dimension to match a test card to the correct sorting location. Third, children must hold in working memory the currently relevant rules to sort by color or shape. Fourth, habits accumulate during the initial sorting phase that both enhance the strength of representation of one dimension and weaken the strength of representation of the other dimension. For example, if sorting by color during the initial sorting phase, then the representation of the color dimension would be more strongly represented than the shape dimension after the initial sorting phase is completed. When children are instructed to sort by shape during the post‐switch phase, it is necessary to use EF resources to overcome the imbalance in representational strength across dimensions. Thus, this task presents multiple challenges that the cognitive system must resolve.

Recently, this task has been expanded in the NIH Toolbox to provide a graded measure of EF abilities beyond early childhood in a way that is more informative than just the binary pass/fail outcome reported in the original task. The new scoring system integrates reaction times and accuracy to create a more holistic DCCS performance score. This is made possible via the NIH Cognition Toolbox's (Zelazo et al., 2013) inclusion of a block of trials in which the rules are mixed. This block is administered if the post‐switch phase is completed successfully. This mixed block is structured so that participants are cued on one‐third of the trials to sort by the pre‐switch rules and are cued on two‐thirds of trials to sort by the post‐switch rules. Performance can then be scored in terms of both accuracy and reaction time, providing a continuous metric of EF skills.

#### Neural Mechanisms of EF in the DCCS Task

Many previous studies have found associations between the development of EF skills and neural activity. Moriguchi and Hiraki (2009) found that preschool age children who switched rules showed stronger frontal cortex activation while completing the DCCS task compared to children who failed to switch rules. These results are typically interpreted as evidence that brain maturation leads to the ability to activate this brain region, which then causes the development of the ability to regulate behavior (Bunge & Zelazo, 2006). Other studies, however, have demonstrated that manipulations to the DCCS task can impact the ability of children to switch rules and engage the frontal cortex. As discussed above, the DCCS provides visual cues, referred to as target cards, to indicate which features are to be sorted at the two locations for each task. The test cards that children sort are configured such that they match both target cards along different dimensions. As shown in Figure 1a, the target cards are a red circle and a blue star, but the two test cards are a red star and a blue circle. This configuration creates attentional conflict that requires selective attention to focus processing on the task‐relevant dimension. This conflict can be eliminated by having children sort test cards that match the target cards along both dimensions. For example, the no‐conflict version in Figure 1b shows the test cards during the pre‐switch phase as a red circle and a blue star, which match the images on the target cards. Standard conflict cards are used during the post‐switch phase. In this condition, most 3‐year‐olds can successfully switch rules (Müller et al., 2006; Zelazo et al., 2003).

Buss and Spencer (2018) examined neural activation across the standard DCCS task and the no‐conflict DCCS task using functional near‐infrared spectroscopy (fNIRS). Results from this study showed that children who failed to switch in the standard DCCS task, and showed weak frontal cortex activation when doing so, nevertheless showed strong frontal cortex activation when correctly switching rules in the no‐conflict version of the task. Thus, activation in frontal cortex and the ability to switch rules is not only influenced by developmental status but also by children's experiences in the task. That is, the same children that supposedly had an “immature” frontal cortex as evidenced by a lack of activation during the standard task, showed stronger activation when correctly switching rules in an easier version of the task. This finding suggests that frontal activation is not limited by the developmental status of this brain region but is also influenced by children's learning within a task.

#### Explanations of Developmental Changes in Performance on the DCCS Task

The componential view of EF has had a strong influence on theories of EF development. For example, theories of development in the DCCS task have focused on some combination of the inhibition, working memory, and cognitive control components to explain developmental changes in performance on this task (Brace et al., 2006; Diamond et al., 2005; Zelazo, 2004). The most influential of these theories is cognitive complexity and control (CCC) theory put forth by Zelazo and colleagues (Zelazo, 2004; Zelazo et al., 2003). This theory is grounded in rule‐representation processes that are implemented as production rules such as “*if* it is red, *then* sort to this location” and “*if* it is a circle, *then* sort to this location.” In this case, rules that are used gain a higher level of saliency whereas rules that conflict with the rules currently being used are suppressed to lower levels of activation. When instructed to switch to the post‐switch rules, children's representation of the rule for sorting by shape (e.g., “*if* it is a circle, *then* sort to this location”) will be more weakly represented relative to the representation of the rule for sorting by color (e.g., “*if* it is red, *then* sort to this location”). The development of rule‐use abilities is the result of increases in the complexity of rules that children can represent. More complex rule‐representations allow children to behave flexibly based on a nested hierarchy of representations. For example, to succeed in the DCCS task, children could use a production rule such as, “*if* I am playing the color game, and *if* it is blue, *then* sort here; but *if* I am playing the shape game, and *if* it is a circle, *then* sort there.” With this production rule, children can make decisions for an object (a blue circle) based on different properties of that object. Without the ability to nest multiple *if* statements, children will persist in using the more strongly activated rule (i.e., the initial sorting rule) rather than switching to the new rules. Increases in the complexity of rule representation are mediated by changes in the level of representation (referred to in this theoretical perspective as a level of consciousness) that children can attain. By taking time to reflect on the nature of the problem, children achieve higher levels of representation in which greater complexity of rule‐representations can be supported. Mechanistically, children progress to higher levels of complexity of rule representation and LOC as the frontal cortex matures (Bunge & Zelazo, 2006).

#### New Conceptualizations of EF

More recent ideas about EF development, however, have pushed back against a rigid component structure to characterize EF in terms of the processes involved in cognitive control that are inherently contextual. Doebel (2020) describes an alternative perspective in which EF is not a set of separable skills but instead a more general ability to use self‐control. That is, Doebel (2020) proposes that there are not multiple EFs, but instead the emerging ability to use self‐control that is tied to the details of the specific context in which children are behaving. Here, the emphasis is on the child's conceptual structure (e.g., acquired knowledge, values, beliefs, preferences, etc.) that are relevant in a particular context and to which they can ground their use of cognitive control. Thus, the ability to use self‐control will depend on the other factors involved in the context of the task, the child's environment, and the child's previous experience. To the extent that lab‐based measures predict behavior in other tasks or outside of the lab, this is due to the overlap in underlying concepts across measures.

Bardikoff and Sabbagh (2021) recently offered an example of such an approach in which they proposed that developmental improvement of performance on the DCCS task is grounded in a conceptual and abstract understanding of dimensionality. When applied to the DCCS task, this object‐based approach posits that the objects involved in the task—the shapes and colors–are more important for successful completion than the rules of the task—*sorting* by shape and color. In this sense, the emphasis is on the representational content of the task. Representational content refers to the information involved in the task that the child will use to complete the task goals. In this case, the representational content is a conceptual understanding that objects are composed of separable dimensions. Thus, success in the DCCS task hinges on children's conceptual understanding of dimensionality which allows children to use cognitive control to flexibly sort objects.

Bardikoff and Sabbagh (2021) tested the role of dimensionality understanding on DCCS performance by training children on multidimensionality prior to completion of the DCCS. The training consisted of a game where children were shown a black‐and‐white outline of a shape and a colored blob. Children were then instructed to select a colored shape, combining the shape outline and the colored blob. Training on both the relevant feature dimensions (the dimensions used in the canonical DCCS task, color and shape) and irrelevant feature dimensions (in this study, the pattern of an object) lead to better performance on the canonical DCCS task in preschool‐aged children. Thus, Bardikoff and Sabbagh (2021) interpreted these data as indicating that training children on the multidimensionality of objects impacted children's ability to think flexibly about the nature of objects so that they could flexibly apply different sets of rules.

#### Limitations of Current EF Theories

Although these new proposals are noteworthy for shifting away from traditional ideas about EF, two important challenges still linger. First, these proposals do not resolve the tension between generality and specificity because the emphasis is now on the context rather than the domain general cognitive control skill. Thus, the main issue is to specify how the emerging capacity for cognitive control connects to variations in context or children's underlying conceptual structures. Doebel's proposal avoids reification of EF into components by suggesting that EF is comprised of a set of skills whose use depends upon contextual information and factors intrinsic to the child; however, Doebel's proposal also falls short by not specifying *how* these skills are used in context‐specific ways. Because this perspective is a verbally specified theory, accounting for such a complex interaction and the range of factors that could potentially influence the use of control is a daunting task.

Second, these new proposals do not specify how cognitive control is impacted by learning and children's experiences. For example, Bardikoff and Sabbagh (2021) argue that an understanding of dimensionality impacts a child's performance on the DCCS task. Dimensional understanding is described as an abstract form of representation that exerts top‐down control on cognitive processing. This perspective, however, does not specify the link between children's experiences and conceptual structure, nor the link between abstract understanding of dimensionality and the processes of representing and making decisions about multi‐dimensional objects.

To address these challenges, Perone and colleagues (2021) argue that theories should focus on how control becomes a property of a neurocognitive system rather than on skills or components that are used in the service of control. In this regard, dynamic systems theory provides the concepts of self‐organization and autonomy, which can provide new traction to the study of EF. Specifically, when components of a system are coupled to one another, it is possible for the system to behave in ways that are not programmed into the system but are a product of interactions between components (Thelen & Smith, 1994). Such organization arises in a neural system as populations of neurons tuned to dimensions of perception and action interact with one another. These interactions can result in the formation of stable neural states that drive behavior. In the context of the DCCS task, for example, neurons that encode both the color red and the shape star interact and become coupled with neurons that drive the action to sort the card. Moreover, quantitative changes in the properties of these neural populations and how they interact with one another can produce qualitative change in both neural activity and behavior. In other words, the dynamic systems approach addresses the key shortcomings associated with other theories of EF development by providing the tools to think about autonomy in a principled fashion and linking the types of changes brought about through learning and experience to the emergence of EF skills. In this Monograph, we focus on one specific instantiation of a dynamic systems theory called dynamic field (DF) theory (Chapter II).

### Conclusion: Reconceptualizing EF Development to Include Autonomy

EF is critically important in a broad array of developmental outcomes. Yet, empirical and theoretical work have yet to capitalize on the centrality of EF. This work has been hampered by the inability to identify developmental mechanisms related to EF development. Consequently, intervention research has failed to improve developmental trajectories by directly training aspects of EF. Historically, concepts surrounding EF have been limited due to the inherent nature of EF which revolves around issues of autonomy and self‐organization. Descriptions of EF centered on components of EF struggle to explain how such components develop in ways that are not self‐referential. More recent conceptualization centered on the context‐dependent nature of EF struggle in different ways to link learning of specific information to variable contexts in which EF is achieved. To address these persistent issues in the study of EF, the approach in our study is to further explore the predictions of a neurodynamical model (a DF model). Specifically, this model looks beyond EF itself to identify learning‐based mechanisms of development. This work shows how a learning process that associates labels with visual features can explain the development of EF. As labels are learned for visual features and dimensions, multimodal representations are built that provide neurocognitive mechanisms for directing attention to task‐ or goal‐relevant features of objects. As label representations are strengthened in the model, aspects of EF are displayed as properties of neural interactions. In the next chapter, we discuss the computational properties of this model, highlighting how the model builds EF processes from perception‐action systems involved in object representation and label learning. We summarize the empirical support for this model and outline the specific predictions that we aimed to test in the current study. This chapter (Chapter II) also concludes with a conceptual summary of the DF model for readers not familiar with computational approaches to neurocognitive development.

## II EF Through the Lens of DF Theory

### Introduction to DF Theory

In this chapter, we describe a neurocomputational model grounded in dynamic field (DF) theory. DF models have been used to replicate human data in numerous studies of both children and adults across several tasks of EF and word learning (Schoner et al., 2016). In DF theory, behavior arises from interactions between populations of neurons that are either excitatory or inhibitory. For example, a neuron that responds to the blue hue will increase the activation of other neurons that also prefer the blue hue and inhibit activation from neighboring neurons that respond to other hues. These interactions between neurons are what eventually drive an individual's behavior.

The model is composed of two primary systems. An object representation system builds representations of multi‐feature objects (e.g., a red star) within neural populations that are tuned to visual features and spatial location. An object representation arises as a pattern of activation for visual features within different visual dimensions (e.g., color and shape) along the spatial dimension. The model also includes a label representation system. This system implements processes related to word learning by forming associations between labels (e.g., “red” or “color”) and visual features (e.g., the red hue) and dimensions (e.g., color). The dimensional labeling system is reciprocally coupled with the object representation system such that activation for visual features can be boosted by activating labels (and *vice versa*). In this way, the model can use its understanding of labels to prioritize task‐relevant visual information and follow rules. The stronger the connection between labels and visual features, the better the model performs on the DCCS task. Thus, the primary developmental mechanism implicated by the model is a label learning process. Conceptually, as children's neural representations of shape and color labels improve, performance improves on the DCCS task.

DF theory has been extensively applied to explain and predict children's behavioral responses and neural activation across various findings from the DCCS and other tasks (Buss & Kerr‐German, 2019; Buss & Spencer, 2014, 2018; Perone et al., 2015, 2019). This DF model frames EF around object‐based attention. Specifically, performance is a result of the ability to prioritize the processing of perceptual information based on task goals. Instead of viewing action as a consequence of a “central executive” or homuncular control system, the DF model demonstrates how cognitive control is an emergent property of interactions between neurocognitive systems and the environment. Importantly, this computational framework provides a means of quantifying properties of the neurocognitive system and the various contextual factors as they relate to behavior and development. In this context, rather than EF being defined in terms of structural components such as inhibition or switching, EF is instead defined as a property of how label representations guide object‐based attention. Across various projects, the DF model has provided a rigorous quantitative explanation of behavioral and neural development across a range of different task demands.

### Model Architecture and Dynamics

DF models are built with populations of neurons that are tuned to metric dimensions of perception (e.g., color) and action (e.g., spatial direction of a planned action). Such metric dimensions of neural tuning have been widely reported in various neurophysiological studies (Erlhagen et al., 1999; Jancke et al., 1999; Markounikau et al., 2010). These neural populations interact through local excitation and lateral inhibition tuning profiles. This means that activation of one neuron may either decrease or increase activation of the neurons connected to it depending on the feature preferences of those other neurons. If one neuron is activated for a particular hue of color such as red, for example, it may share excitation with neurons that respond to similar hues. While this neuron is enhancing activation via direct excitatory connections, it also inhibits others through the process of lateral inhibition that is mediated by inhibitory interneurons. For example, another neuron in the population may activate in response to a different color, such as blue. While the neuron that reacts to red is activated, the neuron that is responsive to blue receives inhibitory input. This pattern of excitation and inhibition make it possible for neurons to produce the stable neural states that are needed to drive behavior: local‐excitation enhances activation of neurons participating in a representation while lateral‐inhibition suppresses other neurons. Stability is an important dynamic because neurons receive noisy inputs from the world and from other neurons to which they are connected. Neural states must have a mechanism to protect against these sources of noise so that a representation can be maintained over time to drive a behavior. This pattern of neural connectivity provides a general mechanism by which neural populations can produce such stable and self‐sustaining states (Schoner et al., 2016).

DF models make a strong commitment to embodiment by using neural populations tuned to dimensions of perception and action. That is, the models are built around the interface of the body with the environment, grounding representations in sensory surfaces and dimensions of the motor system. DF models, however, are not limited to simple perceptual and motor representations. These dimensions can be combined in DF models that build more elaborate and abstracted representations, as we will describe below. By combining dimensions of perception/action representations, models can perform complex tasks such as building representations of multi‐feature objects (Johnson et al., 2008), making sorting decisions as in the DCCS (Buss & Spencer, 2014), searching for visual objects in a cluttered scene (Grieben et al., 2020), or learning words (Bhat et al., 2022). Thus, DF theory explains behavior through the interactions within and between populations of neurons and through these interactions, it is possible to produce more complex or abstract representations.

**Figure 2:**
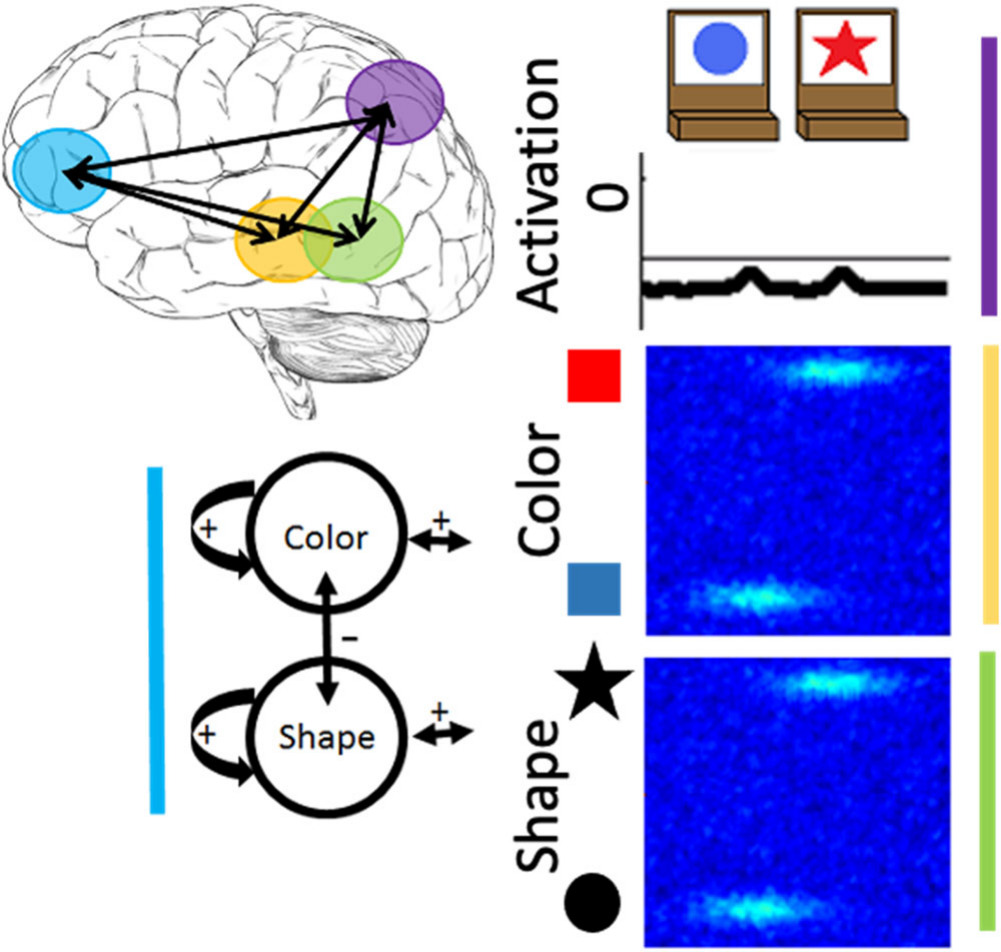
Architecture and cortical mapping of components of the dynamic neural field model used to simulate the dimensional change card sort task.

We have previously developed a DF model that simulates a wide range of behavioral findings with the DCCS task; made predictions about behavior in novel variations of the DCCS task (Buss & Spencer, 2014); simulated neural activation and made novel predictions about neural activation (Buss & Spencer, 2018); generalized to explain developmental changes in a set of other tasks involving categorization and priming (Buss & Kerr‐German, 2019); and predicted the impact of prior exposure to perceptual dimensions (Perone et al., 2015, 2019). The model architecture includes an object representation system. In this system, there are three fields: (1) a spatial motor planning field corresponding to processing within the parietal cortex, a (2) color‐space field, and (3) a shape‐space field corresponding to processing within the temporal cortex (Figure 2). The model builds representations of objects by binding feature dimensions to a common spatial frame of reference. Thus, the color‐space and shape‐space field are composed of neural units that have a joint preference for a visual feature and a spatial location. These fields are reciprocally coupled to the spatial motor planning field. That is, the color‐space and shape‐space fields send excitatory activity to the spatial motor planning field, and the spatial motor planning field sends excitatory information back to the shape‐space and color‐space fields. Lastly, each field has an inhibitory layer which provides lateral inhibitory input at the location of activation within each field. As discussed above, the inhibitory population is needed to stabilize activation of a peak and to force selective activation dynamics. That is, through lateral inhibition, each field is only able to form a peak at a single location.

**Figure 3:**
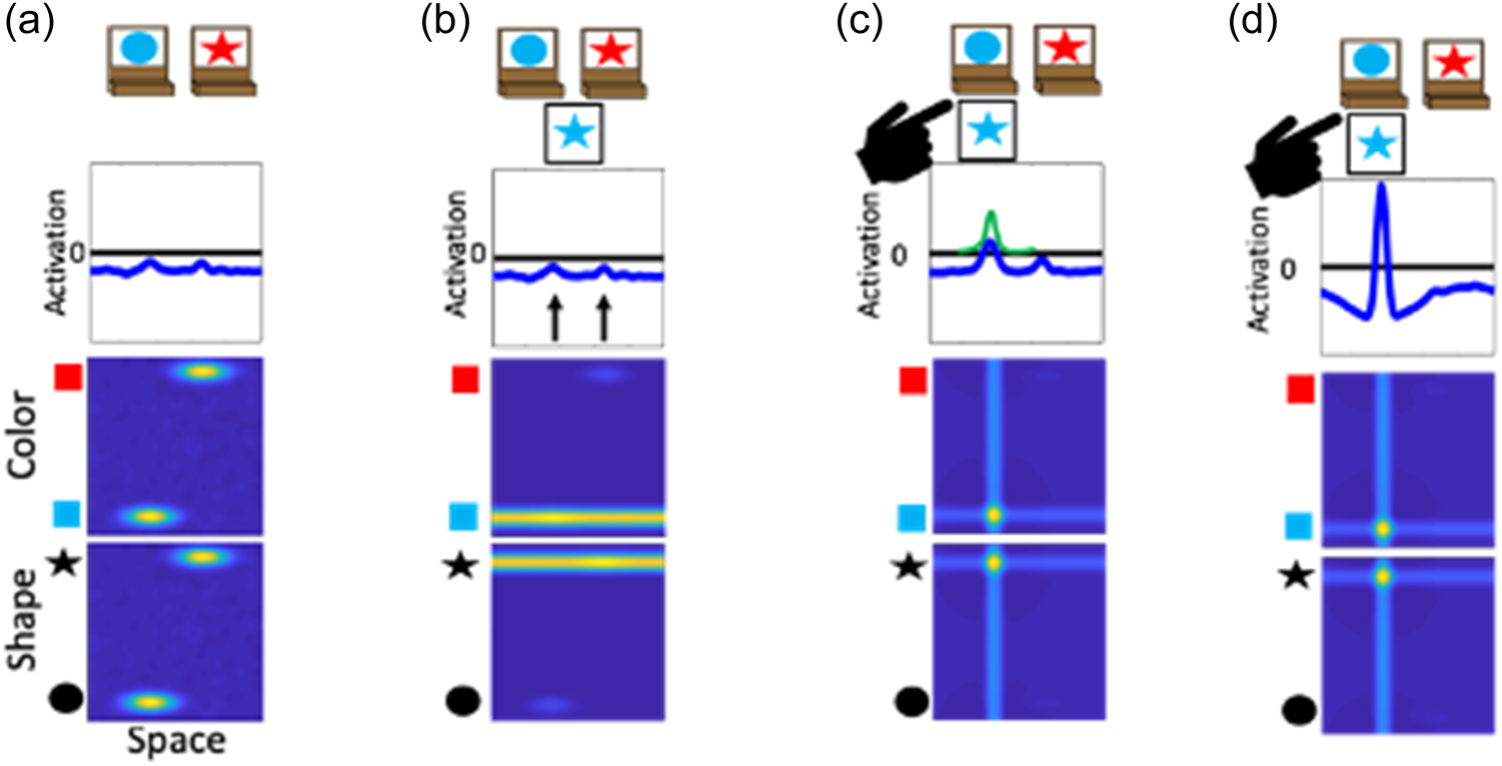
Example model dynamics during a trial in which the experimenter demonstrates how to sort a blue star by color. Panel (a) shows the presentation of the target cards to the model. Panel (b) shows the onset of the presentation of the target card to the model. Panel (c) shows the addition of a spatial input at the leftward location reflecting the demonstration of where to sort this target card. Panel (d) shows the model after a robust representation of the target card at the leftward location has been built.

To illustrate the dynamics and interactions that produce object representations in the context of the DCCS task, Figure 3 displays the sequence of events during a trial in which the experimenter sorts a card to a tray to demonstrate the color rules. First, in Panel a each field has inputs corresponding to the locations of the features on the target cards. In the spatial field, there are two sub‐threshold inputs at the leftward and rightward locations. In the color‐space field, there are sub‐threshold inputs for blue at the left and red at the right. Similarly, in the shape‐space field there are sub‐threshold inputs for circle on the left and star on the right. In Panel b, the model is illustrated shortly after a test‐card is presented to the model. In this example, the model is shown a blue star. The features blue and star within the color‐ and shape‐space fields are ridges of input across the entire spatial dimension. At this point, there is no information about the spatial localization of these features in the context of the task‐space. In Panel c, the model is shown after the spatial demonstration of sorting this card to the leftward location. Of note, there is a strong spatial input to the leftward location in the spatial field (plotted in the green line). This is creating ridges of input at the leftward spatial location across the color‐ and shape‐space fields. Finally, in Panel d the model is shown after the formation of the object representation is complete. Here, the model now has peaks of activation across all three fields at the leftward spatial location. That is, the model has activated the blue feature and star feature at the leftward spatial location to represent the sorting of the blue star object to the leftward location.

**Figure 4:**
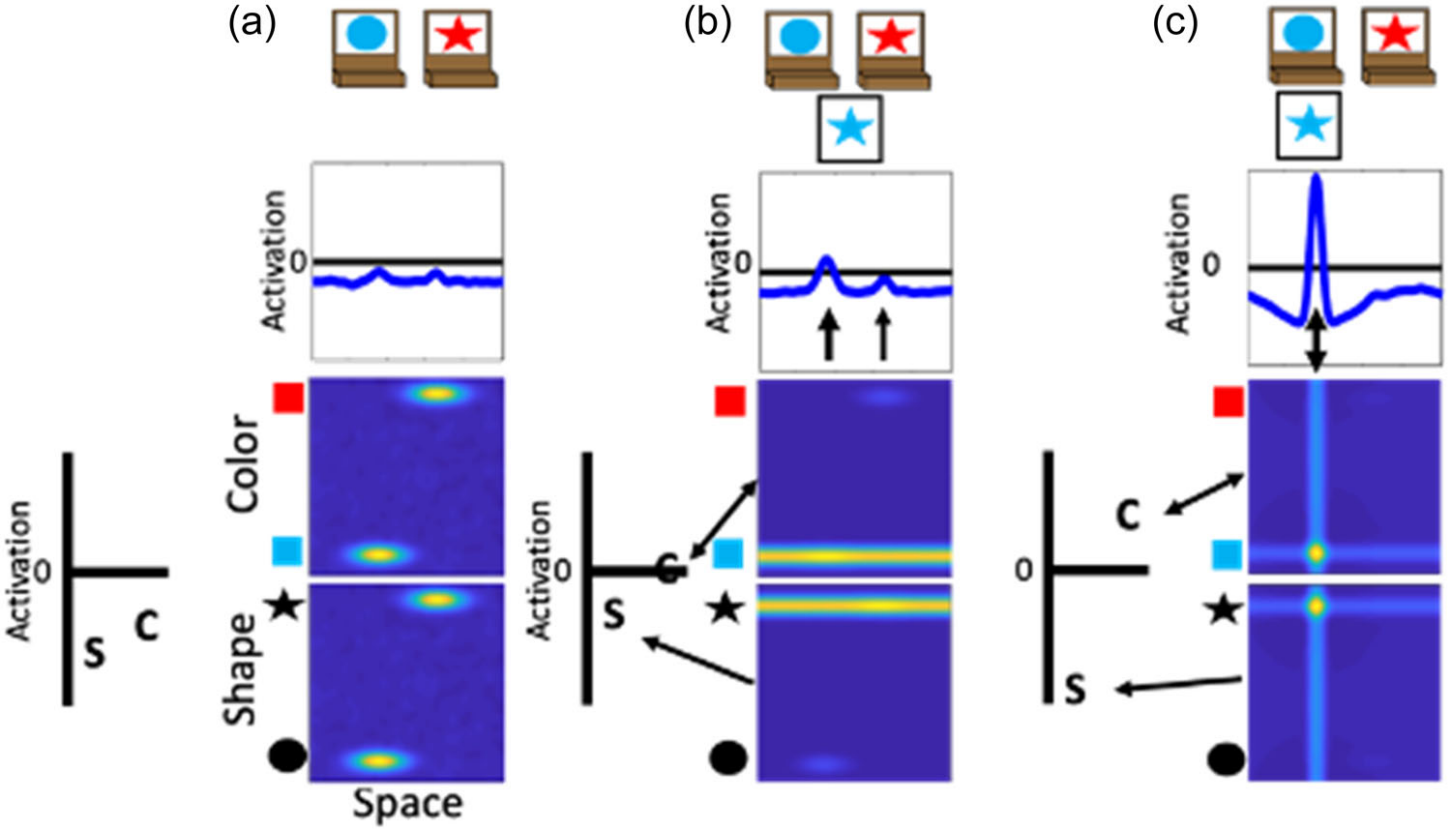
Example model dynamics illustrating autonomous rule‐use of the dynamic field (DF) model. Panel (a) shows the presentation of the target cards to the model wtih the addition of the dimensional label representations. The color (c) node is boosted relative to the shape (s) node because the model has been instructed to sort by color. Panel (b) shows the onset of the presentation of the target card to the model. Activation from the feature‐space fields is being output to the dimensional label nodes and the spatial field. Panel (c) shows the model after a decision has been made to sort the test card to the leftward location. This decision is made because the color label node has been activated which has suppressed activation of the shape node and boosted activation of the color‐space field.

In the above example, the experimenter provides the spatial location information (illustrated as the green input line at the leftward spatial location) needed to overcome the spatial conflict between features on the test card. That is, the blue feature overlaps with the target card input at the leftward location, whereas the star feature overlaps with the target card input at the rightward location. A second component is needed for the model to overcome this imbalance and sort in an autonomous fashion. Specifically, the model also contains a dimensional label system corresponding to processing in the frontal cortex (see Figure 2). This system is simplified as a two‐neuron system that has a representation of the label “color” and the label “shape.” These neurons are reciprocally coupled to the color‐space and shape‐space field, respectively. The model is “instructed” to sort by a particular dimension by providing an input that boosts baseline activity for the relevant label neuron. For example, in Figure 4 the model is instructed to sort by color and the color label neuron is at a higher resting level than the shape label neuron. When a test card input is presented to the model and activation builds within the color‐ and shape‐space fields, excitation is sent to the dimensional label neurons as illustrated by the arrows in panel B. The color label neuron reaches the activation threshold more rapidly than the shape label neuron. At this point, the color label neuron begins to contribute excitation globally to the color‐space field as illustrated by the double‐sided arrow. The arrow illustrating interactions between the shape‐label neuron and the shape‐space field is one‐sided because the shape neuron is receiving excitation from the shape‐space field but is not near the activation threshold to send excitation back to the shape‐space field. With the color‐space field boosted, the input to the spatial field is receiving stronger excitation at the leftward spatial location. This imbalance in input is due to the overlap between the ridge of input from the test card and the localized input for the blue feature on the target card at the leftward spatial location. Finally, in Panel c the model has built a peak of activation in the leftward location in the spatial field, reflecting the decision of the model to sort to the left sorting tray. As a result, the spatial field has stabilized this object representation by sending ridges of spatial excitation to the color‐ and shape‐space fields, forming peaks for the color blue and the shape star at the leftward location. Moreover, local excitation and lateral inhibition interactions have increased the activation of the color neuron far above the activation threshold. The activation of the color label neuron is suppressing the activity of the shape label neuron. The model is, thus, “attending” to the color dimension. Although this figure only provides snap‐shots in time, these dynamics play out in real‐time as activation cascades through the network.

**Figure 5:**
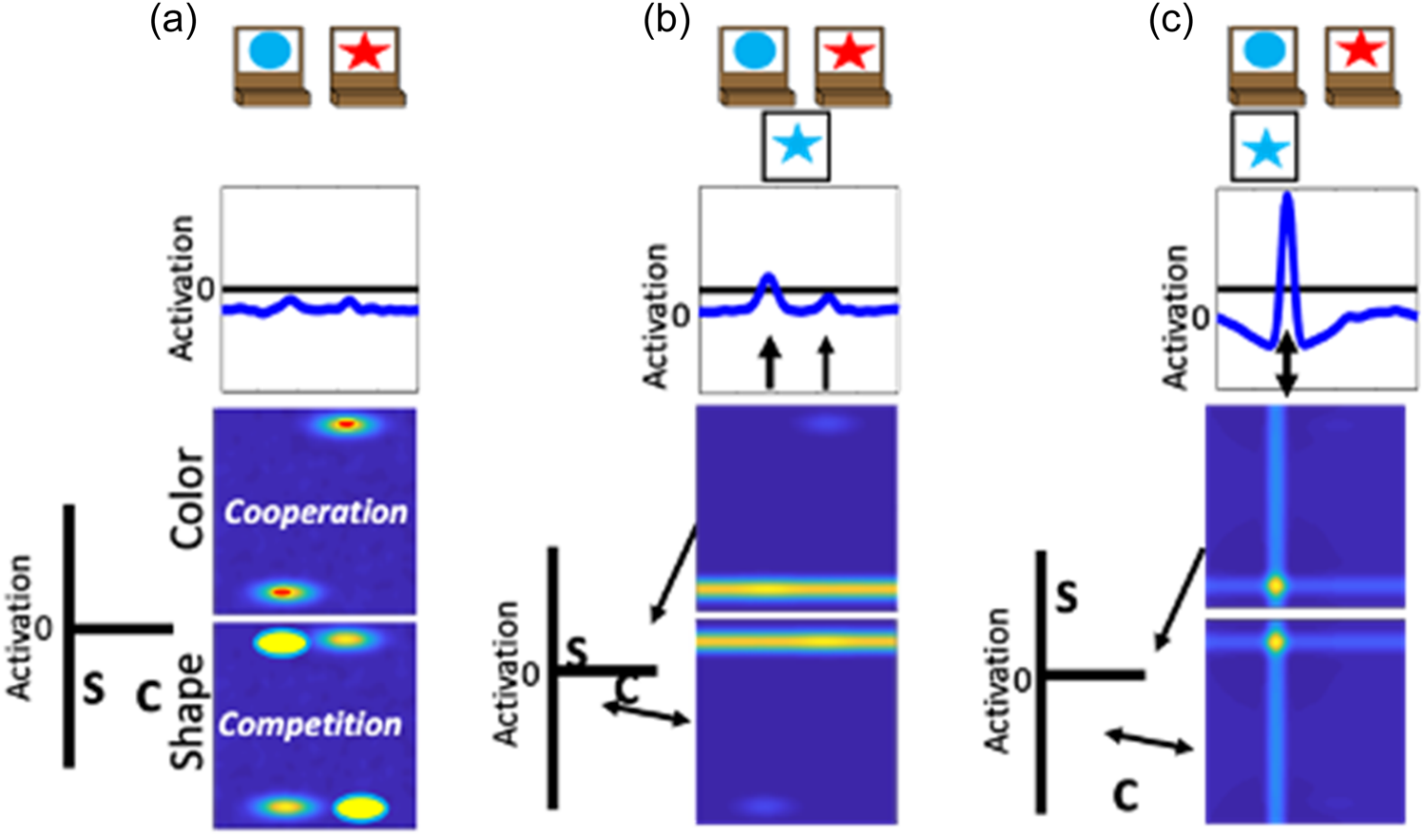
Illustration of model dynamics during the post‐switch phase of a “young” model with weak coupling between labels and visual dimensions. Panel (a) shows the presentation of the target cards to the model with the addition of the dimensional label representations. Note also that there is cooperation between the memory traces and the sorting location in the previously‐sorted color dimension but competition for the shape dimension. Panel (b) shows the onset of the presentation of the test card to the model with a concurrent of activation in the shape‐space field. Panel (c) shows the model making a decision to sort the card to the leftward location even after being “instructed” to sort by shape, representing the perseverative behavior of a young child.

**Figure 6:**
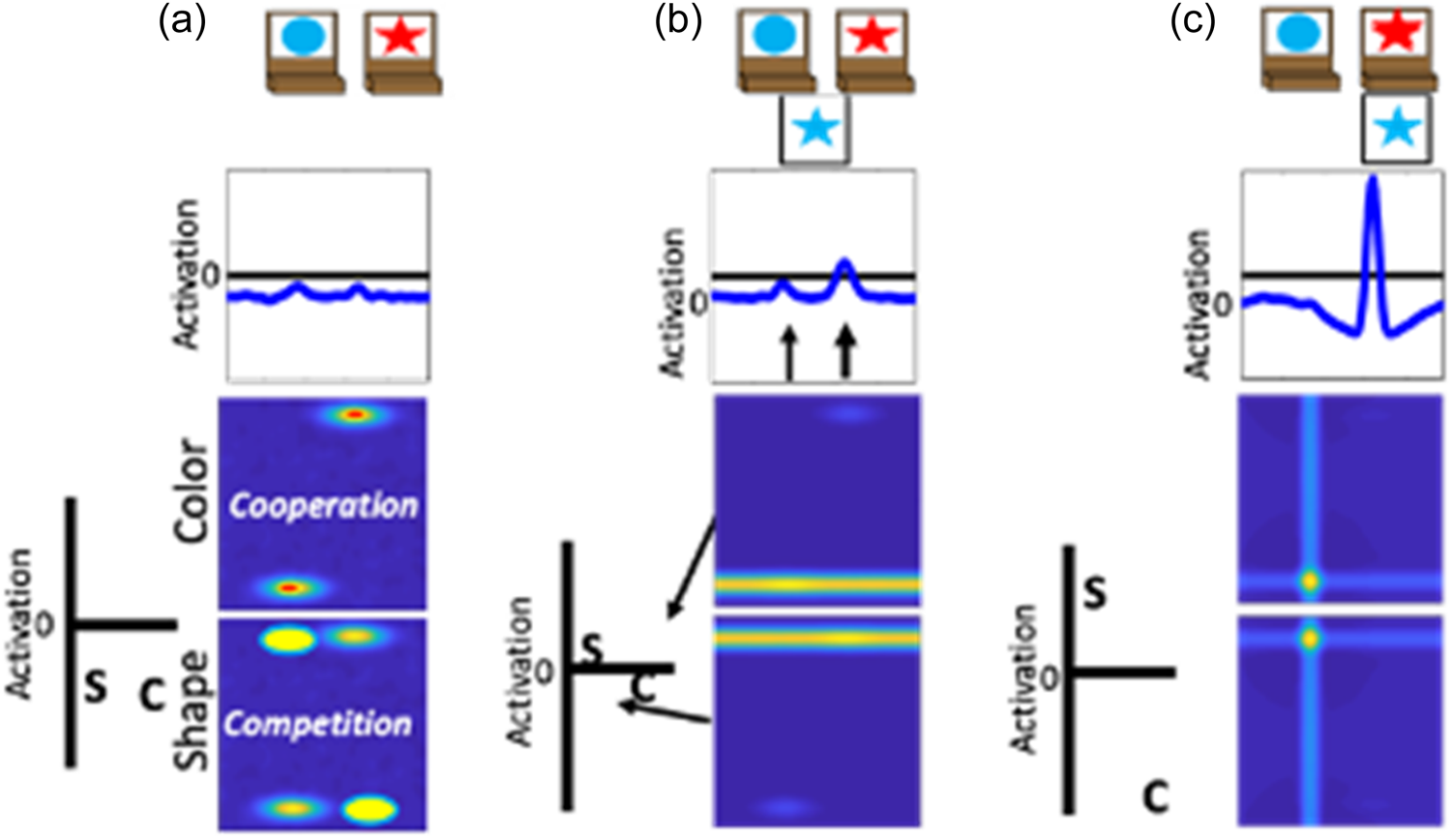
Illustration of model dynamics during the post‐switch phase of an “older” model with stronger coupling between labels and visual dimensions. Panel (a) shows the presentation of the target cards to the model with the addition of the dimensional label representations. Just as in Figure 5, this model also exhibits cooperation within the color‐space field and competition within the shape‐space field. Panel (b) shows the onset of the presentation of the test card to the model with a concurrent boost in the shape‐space field. Panel (c) shows the model making a decision to sort the card to the rightward location after being "instructed" to now sort by shape, representing the ability of an older child to overcome the influence of memory traces and switch sorting dimensions.

A key element to the model's explanation of performance across the different variations of the DCCS task is the formation of memory traces for specific feature‐space conjunctions. In the examples above, the model was sorted by color. But the model, just like a human, is incapable of making a decision about the color of an object without also binding that decision to the object's shape. Based on the conjunction of features on the test cards, the model builds memory traces that link the blue and star features to the leftward location within their respective feature‐space fields. In addition, the model also builds memory traces that link the red and circle features to the rightward location within their respective feature‐space fields. Memory traces are lasting neural representations of the object features in space. Functionally, these traces increase the resting level of neurons so that they are closer to their activation threshold and require less input to become activated. These neural representations create a stronger influence on activation states as they are further reinforced by repeated trials. Because the model sorted by color, the memory traces within the color‐space field will align with the inputs from the target cards. In this case, the color‐space field will be more sensitive for the red feature at the rightward location and the blue feature at the leftward location because those locations are now closer to the activation threshold. However, within the shape‐space field, the memory traces will be at the opposite location of the target card inputs (note the yellow ovals in Panel a of Figures 5 and 6). In this case, the star and circle features will experience lateral inhibition based on the sensitivity to both spatial locations for these features.

Previous work has demonstrated that the performance of the model on the DCCS is impacted by the strength of coupling between label neurons and feature‐space fields (Buss & Kerr‐German, 2019). With weak coupling, as illustrated in Figure 5, the model perseverates. In Panel a, the shape label neuron is now boosted through the instruction input (telling the model to sort by shape) and is closer to the activation threshold than the color label neuron. In Panel b, a test card is presented as in the previous examples. In this case, however, the correct response would be to sort to the rightward location because the red feature is located on the target card on the right. As can be seen in the spatial field, however, there is more input being sent to the leftward location due to the combination of memory traces across the shape‐ and color‐space fields at this spatial location. As a result, the model sorts this test card to the leftward location as shown in Panel c. This decision is made despite the model correctly engaging activation of the shape label neuron. This failure represents the behavior that a young child would show and is the result of weaker coupling between feature labels (“color” or “shape”) and the feature dimension (color and shape).

With stronger coupling, as illustrated in Figure 6, the model correctly updates its behavior and matches by shape. In this figure, the model is given the same test card as in Figure 6 and the correct response would be to sort to the rightward location. In Panel b, however, there is now stronger activation at the rightward location in the spatial field. This is due to the stronger boosting of the shape‐space field. The input for the star feature overlaps with the target card input at the rightward location. The target card inputs are stronger than the memory traces. Thus, the star feature is closer to threshold at the rightward location than the leftward location. By more strongly engaging activation of the shape‐space field, the rightward spatial location is now more strongly activated than the leftward spatial location in the spatial field. As a result, the model correctly selects the rightward location as shown in Panel c.

Distributions of parameter values were generated for the strength of coupling between label neurons and feature‐space fields and individual parameter values from these distributions were applied to individual runs of the model. Over a population of model simulations each with its own unique parameter settings, the model reproduced the pattern of behavior of 3‐ and 4‐year‐olds across a wide range of variations in the DCCS task. This includes situations in which perseveration of 3‐year‐olds persists despite manipulations to visual features between the pre‐ and post‐switch phases as well as manipulations that improve the performance of 3‐year‐olds. Moreover, the model has been used to predict the behavior of children in new versions of the task that manipulate the spatial structure between the pre‐ and post‐switch phases (Buss & Spencer, 2014). The model also explains the brain‐behavior relationship by simulating the increase in frontal cortex activity observed for children that switch rules compared to children who fail to switch rules. In this context, the model also generated predictions that children who perseverate in the standard task, and show weak frontal cortex activation when doing so, nonetheless show strong frontal cortex activation when correctly switching in an “easier” version of the task. This difference in activation between conditions within the same children is a result of memory traces aligning with the target card inputs within the feature‐space field that is relevant for the post‐switch phase. This alignment between target inputs and memory traces creates a stronger input from the feature‐space fields to the dimensional label neurons, thus increasing the activation in this component of the model that corresponds to the frontal cortex (Buss & Spencer, 2018). Lastly, the model is not a special‐purpose EF model. It generalizes to explain performance on other tasks that do not explicitly involve the use of verbal rules or labels (Buss & Kerr‐German, 2019). That is, the same model operating under the same parameters can generalize to explain performance on categorization and priming tasks simply based on changes to the input that reflects the details of these other tasks.

The key novel aspect of the DF model is the grounding of rule‐use in object representation and label learning processes. We have previously published data showing that dimensional label learning impacts the development of EF (Lowery et al., 2022). A prediction of the DF model is that measures of dimensional label learning should be related to performance on EF tasks that involve attention to visual dimensions, such as the DCCS task, but not on other measures of EF that do not have this task demand. For example, the flanker task assesses spatial attention and should be unrelated to the dynamics involved in dimensional label learning. In the flanker task, children are instructed to attend to an arrow in the center of the screen and execute a left/right button response based on the direction that the arrow is pointing (i.e., press the leftward button if the central arrow is pointing left and press the rightward button if the central arrow is pointing right). Participants are typically slower and more error‐prone when the flanking stimuli are pointing in the opposite direction as the central stimulus (Rueda et al., 2004). Because this task does not involve any dimensional labeling like the DCCS does, it would not be expected that dimensional labeling abilities would affect performance. We previously conducted a longitudinal study comparing dimensional label production and comprehension to the DCCS and flanker tasks. We found that activation in left middle frontal gyrus during dimensional label production at 33 months of age was positively associated with performance on the DCCS task at 45 months of age, but was unrelated to any aspect of performance on the flanker task (Lowery et al., 2022). That is, the more strongly participants activated this region at 33 months of age during dimensional label production, the better they performed on the DCCS task a year later. This evidence suggests that the neural mechanisms supporting dimensional label comprehension and production also generalize to impact performance on specific measures of EF. This study by Lowery and colleagues provides a breakthrough in how we understand EF development but has several limitations regarding methods and results. Most importantly, this study administered the dimensional label learning (DLL) task at 33 months but not again at 45 months. Because of this, we cannot assess the growth in dimensional labeling abilities in the children and cannot relate this growth to individual differences in DCCS performances at 45 months. The current study administered the DLL task at both 30 and 54 months, allowing us to measure this growth in labeling ability from child to child.

#### How DF Theory Explains EF Development

The DF model can relate to various aspects of previous theories and debates surrounding the development of EF. As argued by Doebel (2020), the conceptual representations required by a task impact the ability of children to exert control. In the context of the DF model's explanation of the DCCS task, the relevant conceptual structures are dimensional label representations. In contrast to Doebel's proposal, however, control is situated within the dynamics of an autonomously behaving neurocognitive model. In this sense, control is a property of the neurocognitive system. Also similar to the argument put forth by Zelazo and Carlson (2023), there is value in having domain‐general cognitive skills that can transfer across contexts. In this case, dimensional label representations can be used in any number of contexts to guide attention to visual dimensions. As illustrated by Buss and Kerr‐German (2019), dimensional label representations have also been used to explain associations in performance on a priming and a categorization task that do not involve explicit use of verbal labels. Lastly, in relation to the dimensional understanding explanation, the DF model provides a way of grounding dimensional understanding in a label learning process. The primary result from Bardikoff and Sabbagh's (2021) multidimensionality training is that training on the separability of visual dimensions that comprise objects conferred a benefit to subsequent performance. This finding suggests that performance on the DCCS involves a general understanding of the dimensional nature of objects. The key distinction between these explanations is that the notion of dimensional attention provided by the DF model is grounded in children's learning about label representations, visual features, and visual dimensions. The implementation of these processes provides a formalized explanation across a wide range of manipulations to the objects and contexts involved in the DCCS task. The notion of dimensional understanding in Bardikoff and Sabbagh's (2021) perspective, however, is grounded in an abstract conceptual understanding of the separability of dimensions which was manipulated in their task by providing experiences with separated and integrated dimensions. By highlighting the multiple dimensions of objects as in their task, children's representations of dimensions may have been boosted via memory traces, making it less imperative that the child has a strong representation of object‐features and their labels.

#### DCCS and EF: The Influence of Language

The DF model suggests that label learning plays a central role in performance on the DCCS task. Previous work has demonstrated the influence of labels on DCCS performance. For example, Yerys and Munakata (2006) showed that children were better at switching if the pre‐switch was instructed with generic language, such as playing a “sorting” game rather than the “color” game. Moreover, children were also better at switching if novel features with novel labels were used during the pre‐switch phase. In both cases, the lack of specific or familiar language during the pre‐switch phase builds up a weaker bias for the pre‐switch dimension. In another line of work, Buss and Nikam (2020) showed that the label “shape” is much less frequent than “color” in the CHILDES database, a database of recordings of verbal interactions between children and their caregivers (MacWhinney, 2000). They then tested 4‐year‐olds, who would typically pass the DCCS, in a new version of the DCCS that only provided the labels “shape” or “color” during the instructions. For example, children were told, “We're going to play the shape game. Put this shape here (pointing to one target card) and this shape there (pointing to the other target card).” Children perseverated at a significantly higher rate when switching to shape if only the label “shape” was used in the instructions. This finding suggests that the quality of learning for specific labels impacts how well children can use those labels to guide attention.

Beyond the DCCS, various other lines of work have identified language effects on EF measures. There is often a bilingual advantage on measures of EF (Bialystok & Martin, 2004; Poulin‐Dubois et al., 2011), suggesting that learning multiple languages facilitates EF skills. This finding, however, is controversial because the association between bilingualism and EF varies based on the cultural context from which bilinguals are sampled or whether potential confounds are accounted for in analyses (Dick et al., 2019). Linking the effects of label learning and SES, previous research has also demonstrated a profound language input difference across SES. Research has identified an effect commonly referred to as “the 30 million‐word gap,” in which children from low SES backgrounds receive significantly less language input (Golinkoff et al., 2019). This finding could indicate a mechanistic relationship between language and EF that can be moderated by factors such as SES. As we reviewed above in the context of the CHILDES database, the relative frequency of specific labels, in this case “color” and “shape,” impacts performance on EF tasks. Thus, global differences in language input are likely to also be associated with differences in dimensional label input and, relatedly, measures of EF development.

Cognitive control abilities and dimensional attention are also evident in non‐human primates that do not have language abilities (Beran et al., 2016). It is important to point out, however, that such performance requires extensive training to associate cues with visual dimensions. In many ways this is consistent with the framework sketched out the DF model. Language is not a special process in the model; rather, the attentional function of language is a byproduct of associating representations across different perceptual dimensions. Thus, training non‐human primates to attend to visual dimensions, for example, would be the result of artificial associations between cues and visual dimensions which are a natural product of label learning.

DF models also provide potential avenues to think about how factors such as SES and culture influence the developmental trajectories of EF. Bhat et al. (2022) recently developed the word‐object learning via visual exploration in space (WOLVES) model, which is an expanded DF model that autonomously learns labels through cross‐situational exposure to labels and objects. In other words, WOLVES represents the ways that language can both affect and be affected by perception of the child's enviornment. This framework provides an opportunity to probe how exposure to language structures the representations that children come to use for EF skills. WOLVES may, then, be able to explain how unique aspects of children's learning can create individual differences in children's development in relation to cultural or SES factors.

### Conclusions: Model Summary and the Current Study

In the current research project, we more comprehensively explored the relationship between dimensional label learning and EF. The current project builds upon a decade of theory development which has formulated a DF model that explains a wide range of findings with the DCCS, predicted children's performance in novel conditions of the DCCS, predicted the impact of prior exposure to shape and color dimensions, simulated developmental changes in hemodynamic responses, and predicted patterns of neural activation in novel conditions. Across all these different findings, the unifying explanation is that dimensional label learning contributes to the development of EF and generalizes to not only explain and predict behavioral data, but also explains and predicts patterns of neural activation over development. No other theory achieves nearly this level of quantitative explanation of data. Very little work, however, has identified the neural mechanisms of dimensional label learning and their impact on EF development.

In the previous project by Lowery et al. (2022), we examined whether dimensional label learning uniquely predicts DCCS performance among other measures of EF outcomes. However, this project did not assess changes in neurocognitive function associated with dimensional label learning nor did it assess changes in neurocognitive function across other domains. In the current project, we again addressed this question but also asked whether DCCS performance is best predicted by dimensional label learning compared to other factors (i.e., simple EF and dimensional understanding). The goal of the current study was to identify the mechanisms underlying EF development and to determine which measurements from these tasks at 30 months predict EF ability at 54 months. A key developmental mechanism in the model is a label learning processes which cultivates neural representations that can organize decision‐making around visual feature dimensions.

Thus, our primary hypothesis is that neural signatures of dimensional label comprehension and production will predict the future development of EF skills. In other words, we expect neural responses during dimensional label tasks to reflect the quality of neural representations supporting dimensional label learning. These neural responses measured at 30 months should be predictive of performance on the DCCS task at 54 months.

In contrast, other theories have proposed that EF develops via maturational growth (Bunge & Zelazo, 2006; Diamond, 2002). In this case, we might expect that later EF development would be better predicted by early measures of EF. That is, if EF is organized around components that undergo maturational changes over development, then measuring the developmental status of simple EF at earlier points in development should best predict later developmental status of complex EF. We used the Simon task to test this alternative viewpoint. This task was chosen because it has similar arbitrary response selection demands to the DCCS but is simpler so that younger children could perform it. In the Simon task, children need to ignore or inhibit the spatial location of a stimulus which can interfere with the spatial location of a response.

Lastly, another recent proposal reviewed above suggests that children develop an abstract understanding of dimensionality which gives rise to flexibility on the DCCS task (Bardikoff & Sabbagh, 2021). Specifically, previous research on dimensional label learning has examined children's ability to categorize by color or shape using a dimensional matching task that does not use dimensional labels but instead requires children to generalize “sameness” within shape or color dimensions (Sandhofer & Smith, 1999). Thus, we also measured neurocognitive function during this task to determine whether the understanding of dimensionality would predict development of EF skills. The next five chapters present data from a longitudinal study that was aimed at identifying early precursors of later EF.

## III General Methods and Analyses

The current chapter focuses on the general methods and analytical procedures used in this study. Data comes from a longitudinal study in which children were initially recruited at 30 months of age and came back at 54 months of age. Participants were recruited beginning in February 2019 and data collection was completed in April 2022. Data collection was also planned when participants were 42 months of age; however, it was during this time that in‐person research activities were halted due to the COVID‐19 pandemic. Because of this, we were not able to bring children in for the 42‐month sessions but were able to bring back 21 children for their 54‐month sessions.

### Participants

Participants were recruited through a database maintained by the University of Tennessee of families who expressed interest in participating in research activities. Families are enrolled in our database through a variety of methods. Mailers are sent out to families based on birth records provided by the state of Tennessee which provided email links and physical cards that they can return to be added to the database. Families are also recruited by research staff during local community events and settings designed for caregivers and children (e.g., library reading hours). Research staff then contacted families via phone or email for the current study when children were 30 months of age (±2 weeks) and were recruited back to lab when children were 54 months of age (±2 weeks). The average age of participants during the first year of data collection was 30.29 months (*SD* = .52). At follow‐up, the average age of participants was 54.3 months (*SD* = .55). All parents provided informed consent and were compensated with $20 per visit and a $100 bonus upon completion of all four visits over the span of 2 years. Children also picked out a toy to take home after each visit. Research activities were approved by the University of Tennessee Institutional Review Board.

Originally, 67 children enrolled in the study. Of these children, 42 provided complete data during the initial year of participation. Of the 25 that did not provide complete year 1 data, 1 did not continue after consenting, 17 fussed out during the first session of year 1, 3 were dropped due to technical malfunction, 3 fussed out at the second session of year 1.

When research activities were able to resume, 15 had already aged‐out of the follow‐up age and 6 opted not to return, and 1 was color blind leaving 20 children in our final sample that provided complete data (60% female, 100% White, 100% non‐Hispanic/Latinx). The group of 48 participants that were initially recruited but did not end up contributing to the completed analyses, either because they did not complete every session or were deemed ineligible due to colorblindness, was slightly more diverse. Of these 48, 3 participants reported being Latinx, 1 Native American, 2 mixed‐race, and 1 Asian. No included participating families reported being on public assistance, though three dropped families did. Reported annual household incomes ranged from $20,000–$39,999 (*n* = 2 included, *n* = 3 dropped), $40,000–$59,999 (*n* = 6 included, *n* = 14 dropped), $60,000–$79,999 (*n* = 3 included, *n* = 8 dropped), $80,000–$99,999 (*n* = 3 included, *n* = 3 dropped), and $100,000 or more (*n* = 3 included, *n* = 6 dropped). Some families opted not to report their household income levels (*n* = 3 included, *n* = 14 dropped) (Table 1).

**Table 1:** Demographics Breakdown of Children/Families Included in the Longitudinal Sample and Those That Consented for the Study But for Various Reasons (Specified in the Text) Did Not Contribute Data to the Analyses in This Monograph

*Demographic Breakdown of Families Recruited for the Study*			
		Included	Not Included
Family income	20,000–39,999	2	3
	40,000–59,999	6	14
	60,000–79,999	3	8
	80,000–99,999	3	3
	100,000+	3	6
	Not disclosed	3	14
Race	Indigenous	0	1
	Asian	0	1
	Multiple	0	2
	Black	0	1
	White	20	42
	Not disclosed	0	0
Ethnicity	Hispanic	0	3
	Not Hispanic	20	43
	Not disclosed	0	1
Receiving public assistance	Yes	1	3
	No	19	42
	Not disclosed	0	2

**Table 2:** Clusters Identified in Analysis of Functional Near‐Infrared Spectroscopy Data for the Dimensional Labeling Task

*Neural Regions of Activation Associated With the Dimensional Labeling Task*							
		MNI Coordinates					
Effects	Region	*X*	*Y*	*Z*	Volume (mm^3^)	GES	Activation Pattern
Hb	Left SMG	60.8	33.6	27.8	4,144	.030	HbR > HbO
	Right AG	−46.2	68.9	36.2	1,368	.028	HbO > HbR
Dim × Hb	Right iPL	−53.4	55.8	39.6	1,880	.013	Color > Shape
	Left mOG	40.4	74.2	31.1	1,264	.006	Shape > Color
	Left sPL	35.9	55.9	63.1	920	.010	Deactivation
	Left SMG	62.2	27.5	26.3	776	.006	Deactivation
Hb × Task	Right mTG	−65.9	36.3	6.6	1,336	.012	Prod. > Comp.
	Left PCG	58.4	−4.7	20.3	624	.006	Prod. > Comp.
Hb × Dim × Task	Left sPL	30.1	62.7	58.3	2,336	.020	Shape Comp.
	Left iPL	36.1	74.2	41.0	1,912	.027	Color Comp. and Shape Prod.
	Right AG	−52.8	56.1	36	1,848	.014	Shape Comp. and Color Prod.
	Right PCG	−58.6	−8.6	19.1	608	.013	Color Prod.
Hb × Age	Left mOG	28.8	79.7	39.8	1,400	.024	30 months > 54 months
	Right mTG	−63.8	49.0	6.2	616	.023	30 months > 54 months
Hb × Dim × Age	Right iFG	−56.6	−16.6	20.5	2,872	.017	30 months Color and 54 months Shape
	Right sPL	−28.1	65.0	58.8	1,952	.009	30 months Shape and 54 months Color
	Left sTG	64.7	40.1	12.5	608	.009	30 months Shape
Hb × Task × Age	Left iFG	55.6	−11.6	14.9	856	.008	54 months: Prod. > Comp.
	Right sPL	−29.9	60.8	62.4	616	.015	Deactivation
Hb × Age × Dim × Task	Left AG	55.9	54.9	28.7	1,216	.025	Shape Comp. 54 months
	Left iPL	32.6	72.1	46.1	1,056	.019	Deactivation (sp54)
	Left iFG	51.4	−20.8	26.9	768	.005	Color Prod. 54 months

*Note*. AG = angular gyrus; HbO = oxygenated hemoglobin; HbR = deoxygenated hemoglobin; iFG = inferior frontal gyrus; iPL = inferior parietal lobule; mOG = middle occipital gyrus; mTG = middle temporal gyrus; PCG = precentral gyrus; SMG = supramarginal gyrus; sPL = superior parietal; sTG = superior temporal gyrus.

To compare our Knoxville sample (https://data.census.gov/profile/Knoxville_city,_Tennessee?g=160XX00US4740000#populations‐and‐people) to the general population (https://www.census.gov/quickfacts/fact/table/US/PST045222), the median household income in the city of Knoxville in 2022 was $52,826, which is significantly lower than the 2023 median household income of the United states at $75,149. Of the Knoxville population, 17.5% is considered to be in poverty compared to 11.5% of the USA population. Roughly 29% of the Knoxville population identifies as non‐White (15% African American, 2.3% Asian, .06% Native American, 4.2% other race, and 7.3% mixed race), whereas 24.8% of the general US population identifies as non‐White (13.7% African American, 6.4% Asian, 1.3% Native American, .3% other race, and 3.1% mixed race). Lastly, 7.9% of the Knoxville population identifies as Hispanic and 19.5% of the general US population identifies as Hispanic. Even compared to the relatively homogenous populations of both Knoxville and the United States at large, this longitudinal sample is small and demographically homogenous, which we acknowledge is an important limitation and a pervasive issue in developmental cognitive neuroscience research (Qu et al., 2020). We return to this issue in the Discussion (Chapter VIII) and plans to address concerns around generalizability in future work.

We also examined differences in behavioral performance across our tasks between the 20 children who completed both years of data collection and the other 27 children who provided complete year 1 data but didn't return for the 54‐month follow up. Specifically, we compared behavioral accuracy on shape and color label comprehension and production, accuracy on shape and color matching tasks, as well as accuracy and reaction time on the neutral, congruent, and incongruent trials on the Simon task. Across these 12 data points, there were no significant differences between these groups (all *p* > .18).

### Procedure

Children completed a large battery of tasks broken up into two sessions completed each year. At 30 months, children were strapped into a standard highchair and were seated approximately 42 cm away from a touchscreen monitor. Children completed the dimensional label comprehension and production tasks, dimensional matching tasks designed to measure children's understanding of dimensionality, and resting state task during one session. During another session, the children completed the Simon task and a snack delay task not included in the current report. The order of sessions was counterbalanced across children.

At 54 months, children were seated in a chair approximately 42 cm from a touchscreen monitor. During one session, the children completed the dimensional label comprehension and production tasks and dimensional matching tasks. Children were also administered the multisensory attention assessment protocol (Bahrick et al., 2018), but data from this task are not presented here. During another session, the children completed a resting state protocol (Kerr‐German et al., 2022), the DCCS task, and the Simon task.

**Figure 7:**
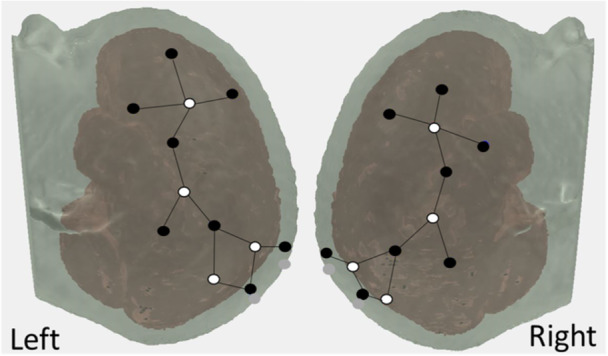
Depicted here is the near‐infrared spectroscopy probe targeting bilateral frontal, temporal, and parietal. Here sources are depicted in white and detectors in black. Lines connecting the sources and detectors display the channels.

During each session, fNIRS data were collected using a TechEN (Milford, MA, USA) CW7 system with 8 sources and 16 detectors. These optodes were configured in a custom cap with 48 channels having 3 cm separation between sources and detectors. Channels were positioned over bilateral frontal, temporal, and parietal cortices. We used two cap sizes (52 and 54 cm) to accommodate differences in children's head sizes across years. Each cap had the same probe configuration and channel separation. Figure 7 displays the configuration of the NIRS probe on a participant's head.

fNIRS is a neuroimaging technique that uses infrared light to measure neural activity. Two wavelengths of light (690 and 850 nm) are emitted and the presence of oxygenated and deoxygenated blood in brain regions underlying the fNIRS probe by measuring the amount of light that is absorbed. Deoxygenated (HbR) and oxygenated (HbO) hemoglobin absorb different amounts of these wavelengths of light, so a measure of how much light is absorbed indicates the presence of these chromophores. Measuring blood flow is an indirect measure of neural activity that is the result of a process called neurovascular coupling. That is, the activation of neurons triggers an influx of oxygenated blood and a relative decrease in deoxygenated blood within the activated region (Moon et al., 2021). The measure obtained by fNIRS is similar to that obtained with fMRI by observing changes in the relative concentration of HbO and HbR. Whereas fMRI measures a blood oxygen level‐dependent (BOLD) signal that is a singular measure of the relative oxygenation in neural tissue, fNIRS measures absolute concentration values of both HbO and HbR. In fNIRS, for a response to be considered relevant for neural activation, there should be an inverse relationship between HbO and HbR with an increase in HbO (Kinder et al., 2022). Statistically assessing both HbO and HbR reduces the influence of motion artifacts and systemic blood flow on functional measurements (Cui et al., 2010).

Because of its ease of use compared to fMRI, fNIRS has made it possible to collect neural activity using the hemodynamic response in very young age groups (Anderson et al., 2017; Ding et al., 2023; Kerr‐German & Buss, 2020; Kerr‐German et al., 2023; Lowery et al., 2022; Zhou et al., 2022, 2024), which have often been previously unexplored. fNIRS also has a much lower startup cost than fMRI, allowing researchers to own their own systems and collect data more independently than collaborating with medical centers or other research institutions. However, fNIRS is not without its limitations. First, there is immense variety in head shapes and sizes. This makes it hard to ensure that channels that are placed on the scalp are mapping onto the same cortical location among participants. This is why comparing channel activity across a sample may not provide an accurate representation of neural activity: there is no guarantee that a channel was placed over the same brain region in every participant. To remedy this, image‐reconstruction methods have been developed that use digitized three‐dimensional spatial locations of the sources and detectors in the fNIRS probe relative to standard anatomical landmarks on the scalp. In this way, channel information can be aligned across participants and registered relative to subject‐specific or age‐appropriate standard brain atlas. After fNIRS data have gone through image reconstruction, standard fMRI analysis methods can be applied to analyze and visualize activation within the brain atlas. The following paragraphs are relevant for readers seeking a deeper understanding of how the fNIRS analyses were conducted.

### fNIRS Setup

First, children's head circumference was measured to determine which size EasyCap (EasyCap GmbH, Woerthsee‐Etterschlag, Germany) to use. Children's heads were then measured from right left preaural areas (space directly in front of the ear) and nasion (space above the nose and between the eyebrows) to inion (ridge in the skull directly above the neck) to find the central point on the top of the head (referred to as cZ). An EasyCap fitted with the array of sources and detectors was then placed on the child's head. The EasyCap has the international 10–20 coordinates marked and the Cz location on the cap was aligned with the Cz marked on the child's head to ensure consistent cap placement across participants. Using a Polhemus (Colchester, VT, USA) Patriot system, the five anatomical landmarks on the scalp (nasion, inion, Cz, and left/right preaural areas) were marked along with the locations of the sources and detectors of the fNIRS probe.

### fNIRS Data Processing

**Figure 8:**
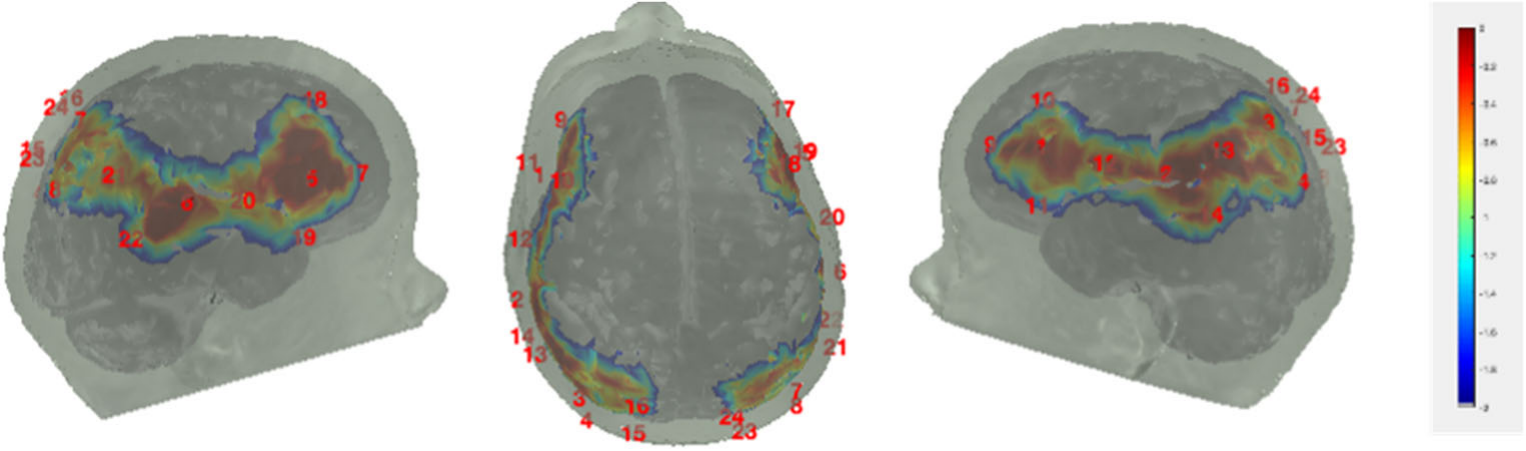
Sensitivity profile for subject number 116 at 54 months.

fNIRS data were processed using an advanced image‐reconstruction pipeline (Forbes et al., 2021). The first step in the analysis pipeline is to create a sensitivity profile that captures the region of cortex from which the fNIRS probe is measuring. Using AtlasViewer (Aasted et al., 2015), a segmented anatomical image is aligned with the digitized locations of the landmarks and fNIRS probe. We used segmented templates corresponding to 30‐ and 54‐month‐olds for data collected at 30 and 54 months of age, respectively. The segmented anatomical image differentiates tissues such as superficial layers of the scalp and skull, white matter, gray matter, and cerebrospinal fluid. Using different parameters for the optical properties of these different tissues (Jacques, 2013), Monte Carlo simulations were conducted using 100 million photons for each wavelength (690 and 830 nm) and each channel in the fNIRS probe to then create a model of the pathway that light will have traveled for each channel and the corresponding tissues that were detected in the anatomical volume (i.e., a sensitivity profile). This sensitivity profile was then clipped to include only spatial regions corresponding to the cortical surface. The depth of the pathway that the light travels is a function of the distance between the source and detector with the signal‐to‐noise ratio decreasing as the separation distance increases. Thus, the 3 cm separation is typical because it optimizes the trade‐off between depth and signal quality. However, because of this constraint, data collection was limited to about 2 cm into the surface of the cortex. Figure 8 illustrates the registration of the fNIRS probe and the sensitivity profile for a representative participant. Analyses were conducted only within voxels for which all participants contributed data.

Next, fNIRS data were processed using Homer2 (Huppert et al., 2009). First, data were converted into optical density units corresponding to the amount of light that is absorbed in each channel. Another common issue with fNIRS data analysis, especially when working with toddlers, is the presence of motion artifacts. Simply put, a motion artifact is a disruption in the conversation between the source‐detector pair due to excessive movement of the participant's head. To identify and smooth out disturbances caused by motion artifacts, the wavelet motion correction function (Molavi & Dumont, 2012) was used (interquartile threshold value of 0.72). Lastly, there are many systematic fluctuations occurring in hemodynamic measures that are faster or slower than the frequency of the expected functional response. These frequencies can be due to factors such as breathing, heart rate, or systemic changes in blood‐pressure (e.g., Mayer waves). To remove frequencies outside of what would be considered due to a functional hemodynamic response, data were bandpass filtered to remove frequencies outside the range of 0.016 and 0.5 Hz.

Next, tools in the NeuroDOT toolbox (Eggebrecht & Culver, 2019) were used to project the channel‐based fNIRS data into the volume of the sensitivity profile. First, the sensitivity profile was pruned to remove values lower than .01. Next, a global signal regression is carried out on the optical density data. This step removes changes occurring globally throughout the entire set of measurements and retains signals that are specific to each channel (Murphy & Fox, 2017). This regression also increases the contrast to noise and provides greater spatial specificity of the variance in the data.

The sensitivity matrix was then inverted for each wavelength. This step presents an inverse problem which uses the Tikhonov regularization method to create a Moore‐Penrose generalized inverse that solves for an underspecified solution (*λ* = .01 Tikhonov regularized and *λ*_2_ = .1 spatially variant regularization). These parameters are used to create a balance between the high‐spatial frequency noise and spatial smoothness in the DOT image, to control for the superficial image reconstruction that DOT often produces, and to optimize spatial correspondence with fMRI data (Wheelock et al., 2019).

We then conducted a general linear model to estimate the amplitude of hemodynamic response to each condition. Using the stimulus timing information for each trial in each condition, a 10 s boxcar function was aligned to each trial onset and convolved with a canonical hemodynamic response function. The GLM then estimated the amplitude of change in HbO and HbR within each voxel associated with each condition. Data were then transformed into a common group space (MNI). Lastly, an ANOVA was carried out with factors of interest (described in subsequent chapters) using 3dMVM in AFNI (Chen et al., 2014). 3dMVM contains many advantages over other ANOVA methods such as 3dLME. 3dMVM does not limit the number of explanatory variables, allows for missing data in some subject files, accepts covariates, and can be corrected for sphericity. The results of the 3dMVM reveal brain regions with significant effects or interactions. Family wise error was calculated using 3clustsim (Cox et al., 2017) with voxel‐wise *α* < .05 and cluster‐wise *α* < .05.

## IV Dimensional Label Comprehension and Production

In this chapter, we present the methods and results for the dimensional labeling tasks administered at both 30 and 54 months of age. These tasks require children to either verbally label features in the production blocks or to select an object from an array of items using a touchscreen computer monitor in the comprehension blocks. Similar tasks have been previously used to assess children's dimensional label knowledge for color and shape but have not been previously used together in the same group of children (Sandhofer & Smith, 1999; Verdine et al., 2016).

### Method

**Figure 9:**
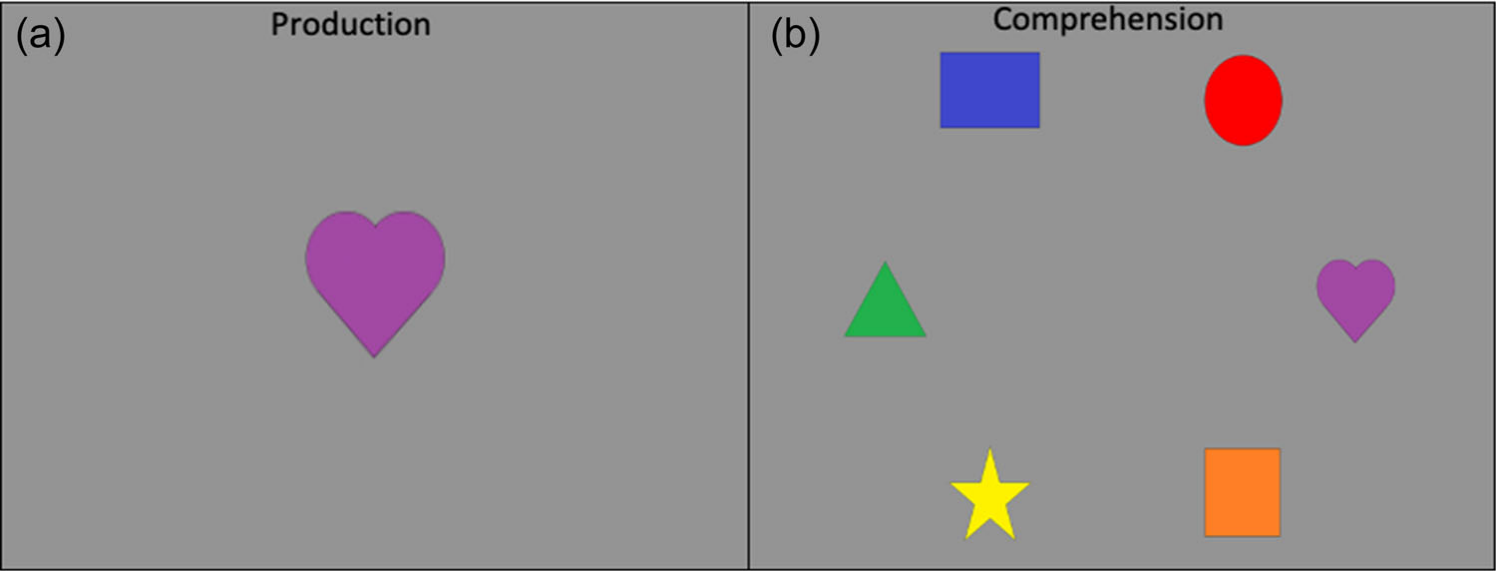
In the production task (a), children see one image appear on the screen and are instructed to produce a verbal label for either the color or shape of the object, as prompted by the experimenter. In the comprehension task (b), children see an array of six objects and are instructed to press the exemplar based on either shape or color, as prompted by the experimenter.

The dimensional labeling task used production and comprehension to assess children's knowledge of color and shape labels. Stimuli were comprised of objects with six canonical shapes (triangle, square, circle, heart, star, rectangle) and colors (red, blue, green, yellow, purple, orange). First, the production task required children to verbally label a single object that appeared at the center of the screen with either a shape or color label (see Figure 9a). The researcher specified which feature dimension the child should attend to at the start of each trial with the prompt, “Can you tell me out loud what shape/color this is?” In the comprehension task, children selected the prompted color/shape from a circular array of six objects with a tap on the touchscreen monitor (see Figure 9b). In this task, children were prompted, for example, with “Can you touch the purple one?” Each child performed four blocks containing 12 trials each of production and comprehension for both the color dimension and shape dimensions (see Figure 9). Children also completed a version of this task with real‐world shape‐based objects that were administered in two additional blocks of production and comprehension. Results from these blocks will not be presented here.

Behavioral accuracy was analyzed using a 2 (Task: Production, Comprehension) × 2 (Dimension: Shape, Color) × 2 (Age: 30 months, 54 months) repeated measures ANOVA. Neural activation was analyzed with a 2 (Task: Production, Comprehension) × 2 (Dimension: Color, Shape) × 2 (Age: 30 months, 54 months) × 2 (Oxy: HbO, HbR) repeated measures ANOVA using 3dMVM (Chen et al., 2014). Family‐wise error calculations (described in Chapter 2) indicated a cluster threshold of 82 mm^3^ was needed for *α* < .05 with voxel‐wise *p* < .05. Average beta values of HbO and HbR were extracted from clusters that satisfied these threshold criteria, and follow‐up tests were performed using SPSS (IBM, version 29). To reduce the dimensionality of the data and simplify the follow‐up tests, we first computed an *activation* variable as the difference between HbO and HbR. In general, functional brain activation is reflected by an increase in HbO relative to HbR levels.

### Behavioral Results

**Figure 10:**
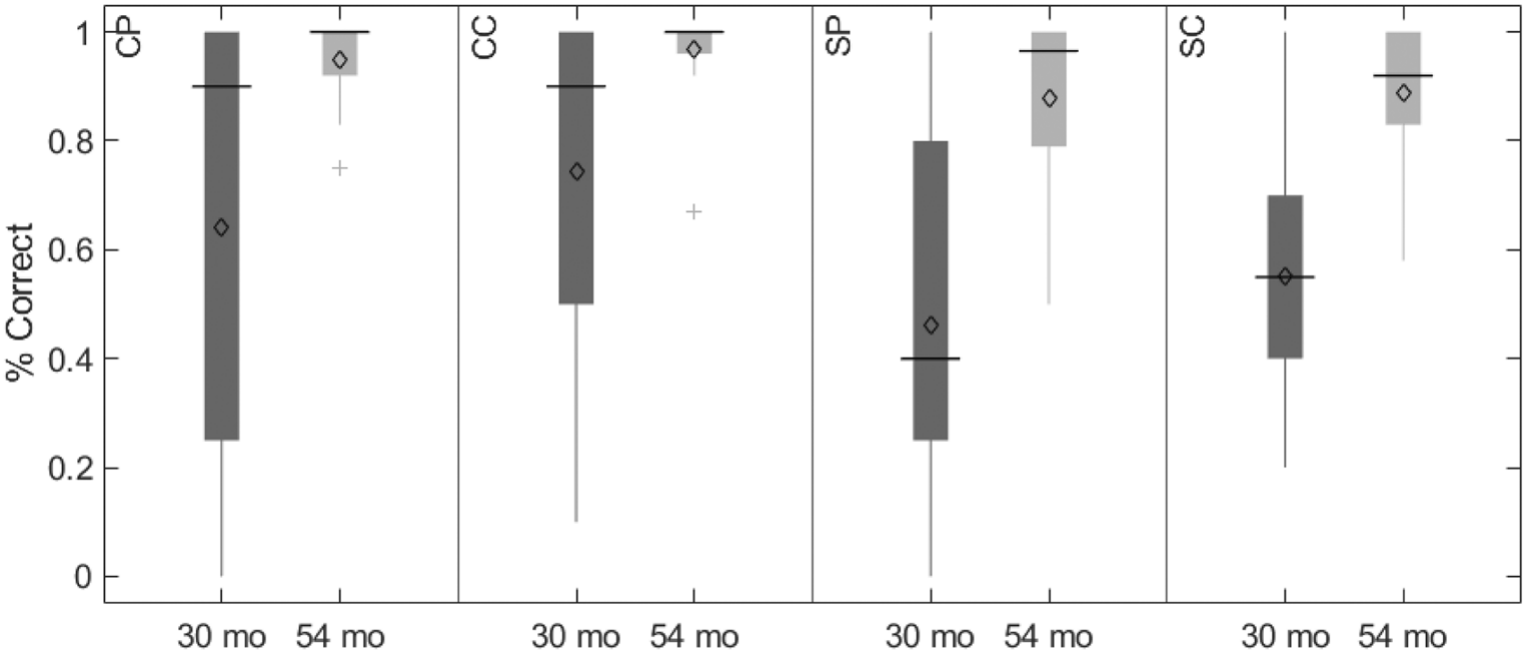
Behavioral results of the dimensional labeling task. Box plots of accuracy data are plotted for color production (CP), color comprehension (CC), shape production (SP), and shape comprehension (SC) trials for 30 months (dark grey) and 54 months (light grey). Means are marked by diamond, median values are marked by horizontal lines, standard deviation is marked by thick bars, confidence intervals representing 95% of distribution are marked by thin bars, and outliers are marked with crosses (+).

Behavioral data are plotted in Figure 10. Unsurprisingly, there was a main effect of age on behavioral accuracy, *F*(1,19) = 35.384, *p* < .001, *η* = .651, such that children performed better at 54 months (*M* = .92) compared to 30 months (*M* = .60). There was also a main effect of task, *F*(1,19) = 8.665, *p* = .008, *η* = .313, such that children performed better on comprehension (*M* = .79) compared to production (*M* = .73). Lastly, a main effect of dimension, *F*(1,19) = 29.486, *p* < .001, *η* = .608, showed that children performed better on color trials (*M* = .83) compared to shape (*M* = .70). No interactions were significant.

These accuracy results give the first insight into the relative rate of label learning across dimensions. Overall, performance on tasks measuring knowledge of color labels were easier than tasks measuring knowledge of shape labels. Previous work has assessed color (Sandhofer & Smith, 1999) and shape (Verdine et al., 2016) labels separately, but no research has directly compared the acquisition of color and shape labels within the same cohort of children. Within both color and shape dimensions, comprehension was an easier task than production for these children at both ages, consistent with general findings on vocabulary comprehension and production differences (Webb, 2008). As described by Sandhofer and Smith (1999), comprehension can be performed via direct associations between labels such as “red” and the red hue. Performing production, however, requires an integrated mapping of dimensional labels (e.g., “color”), featural labels (e.g., “red”), and hues. In this way, the representation of the label “color” can be used to guide attention to the relevant feature and select the appropriate featural label. In other words, it is easier for a child to use the label “red” and select a feature that is directly associated with the label from an array of objects than to use the label”color” to attend to a particular aspect of an object to select another label based on a feature. That is, the label “color” is not directly associated with visual features. Rather, using the label “color” requires understanding both that there are subsets of labels associated with “color” and that those subsets of labels map onto particular visual dimensions of an object's appearance.

### Neural Results

Analyses of the neural data revealed several interesting patterns. In total, 22 clusters showed significant effects during the dimensional label tasks. However, five of these regions showed only deactivation (HbO < 0) and will not be included in further analyses. Table 2 lists the brain regions, coordinates, cluster volumes, and the observed effects for each cluster.

**Figure 11:**
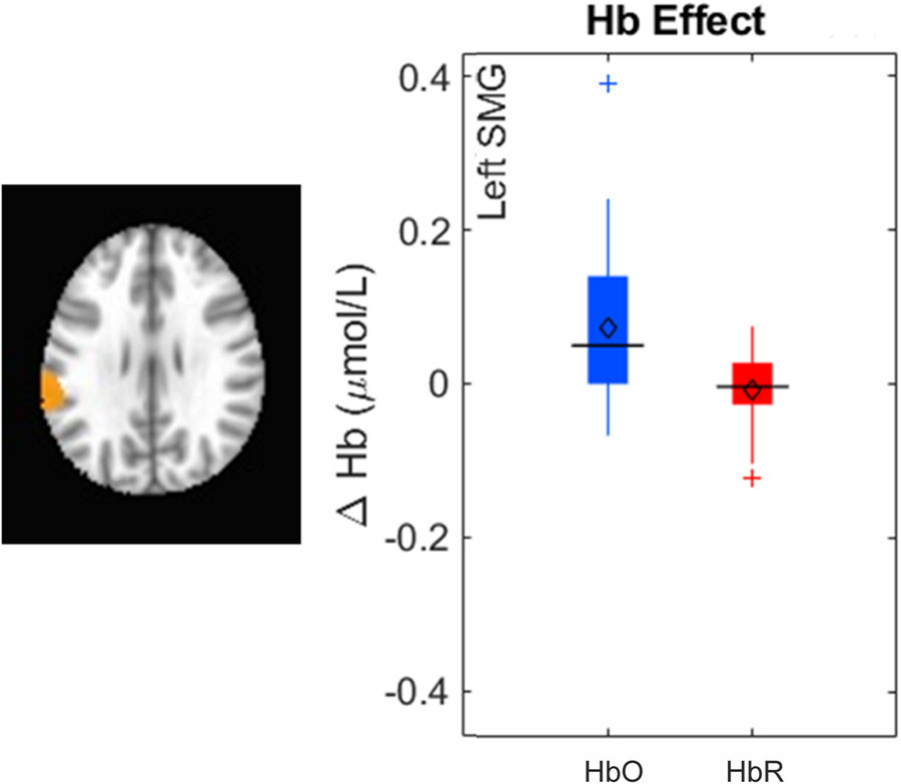
Cluster of neural activation in which a main effect of Hb driven by activation was observed when collapsing by every age/condition. Activation is operationalized as an inverse relationship between oxygenated (HbO) and deoxygenated (HbR) hemoglobin where HbO is higher.

An effect of Hb (Figure 11) was observed in right angular gyrus (AG). In the case of an Hb effect, participants showed general increases in HbO that were not specific to any factor of the task. The AG is a region of the temporal lobe that lies at the convergence of many sensory‐processing areas such that information coming from visual and auditory modalities are integrated in this region (Seghier, 2013). As the AG is implicated in integration of multiple sensory inputs, it is often also associated with processing of word meaning (Binder et al., 2009). Activation of this area could be due to the engagement of associations between the visual (object features) and auditory (verbal feature labels) stimuli during these tasks. An effect of Hb was also found in left supramarginal gyrus (SMG), but this region displayed deactivation.

**Figure 12:**
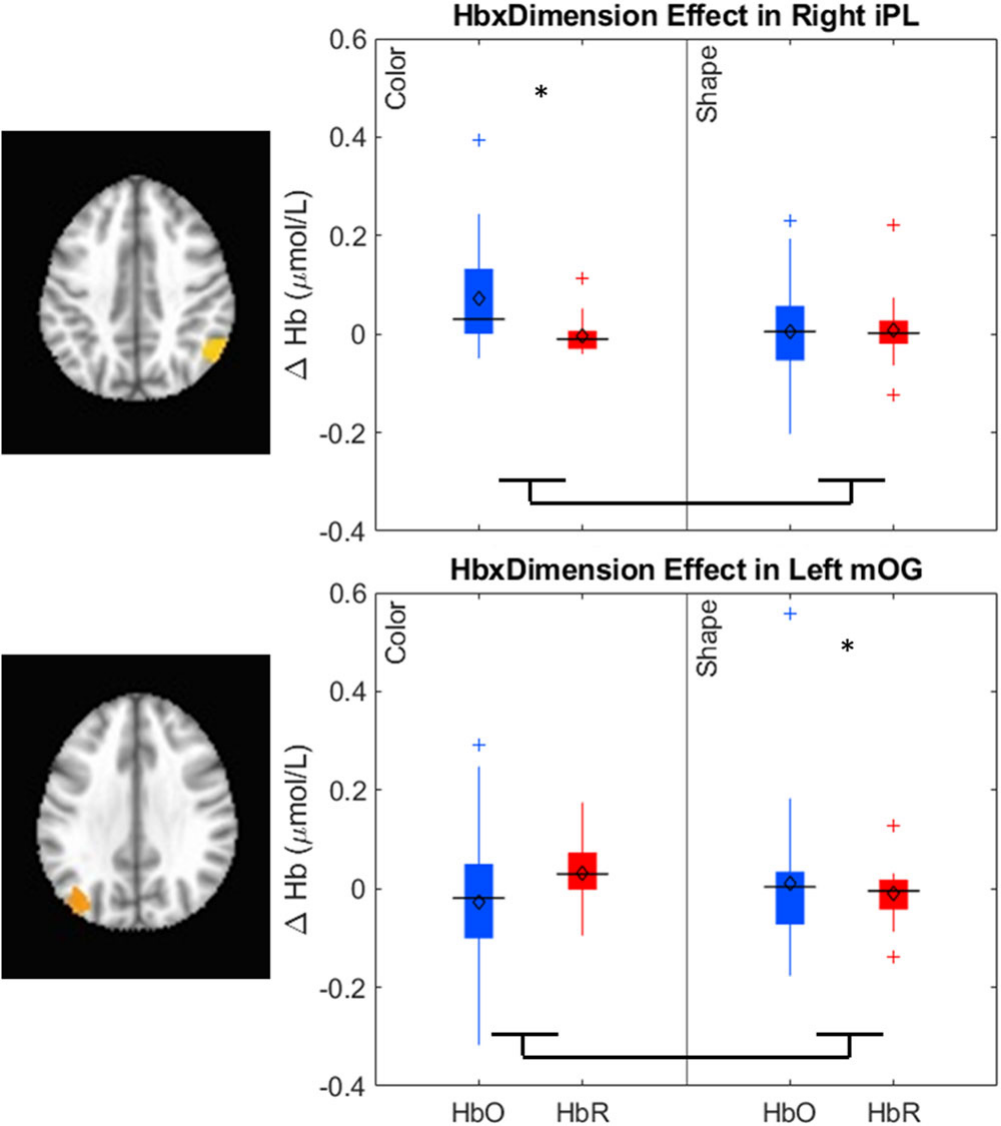
Clusters of neural activation in which an interaction between Dimension and Hb were observed. Right inferior parietal lobule was activated more for color trials (top) whereas left middle occipital gyrus was more activated for shape trials (bottom). Note that black bars indicate the comparisons being made and an * indicates which comparison is showing activation.

An interaction between dimension and Hb (Figure 12) was found in right inferior parietal lobule (iPL) where activation was stronger during color tasks relative to shape tasks, *p* = .003. Another cluster was found in left middle occipital gyrus (mOG) where activation was stronger during shape tasks relative to color tasks, *p* = .022. These indicate that color and shape labels were processed in different neural regions early in development. Both occipital and parietal regions are typically implemented in category learning contexts (Morrison et al., 2015), so these findings are consistent with that literature. Moreover, previous category learning studies have found that as individuals become more proficient at novel category‐identification, a shift occurs from primarily occipital activation to more frontoparietal regions (Pérez‐Gay Juárez et al., 2019). This is because the occipital gyrus is implicated in the processing of basic visual information such as edges and line orientation (Vinberg & Grill‐Spector, 2008), whereas the parietal lobule engages more complex processes like the integration of multiple sources of feature information, such as binding features to spatial locations (Ibos & Freedman, 2014; Shafritz et al., 2002). The different localization of activation that we found for shape and color stimuli could reflect differences in the level of categorization across labels for these feature dimensions. That is, the occipital gyrus was engaged more strongly during shape labeling tasks because children were still processing these features at a lower perceptual level. Activation in the parietal lobule during the color tasks, however, could be due to these children having a better understanding of the broader category of “color.” Children, especially 30‐month‐olds, performed worse on shape trials, indicating that there was less categorization knowledge during shape tasks than color tasks. It is also possible that, because of the difficulty associated with the shape tasks, children relied more heavily on the basic visual information in the stimuli. Additional clusters were found in left superior parietal (sPL) lobule and left SMG, but these regions displayed deactivation.

**Figure 13:**
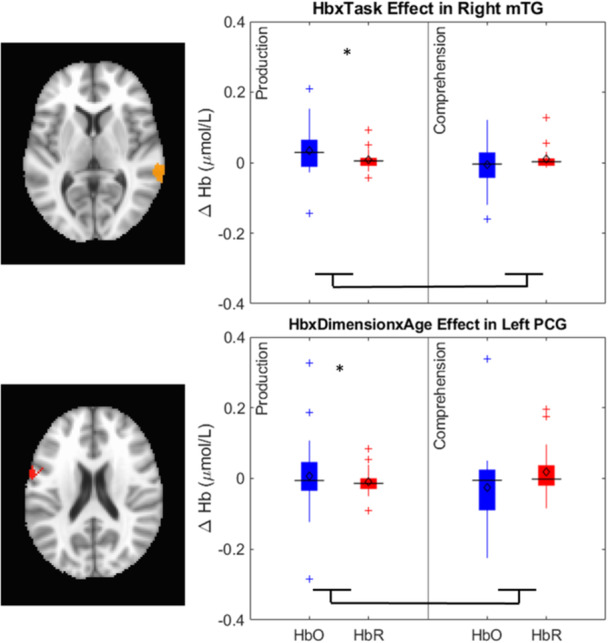
Clusters of neural activation in which an interaction between Task and Hb was observed. Both the right middle temporal gyrus (top) and the left precentral gyrus were more strongly activated for production tasks compared to comprehension.

An interaction between task and Hb (Figure 13) was observed in both right middle temporal gyrus (mTG) and left precentral gyrus (PCG) where activation was stronger during the production tasks (*p* = .004 and *p* = .027, respectively). The fact that there were significant task‐related effects only for the production tasks suggests that these additional neural resources were needed to perform these more difficult tasks. These results are also consistent with research showing that mTG is involved in language processing (Margulies & Petrides, 2013; Noonan et al., 2013; Papeo et al., 2019; Sugimoto et al., 2023). Moreover, PCG, particularly in the left hemisphere, is near Broca's area and is historically a region involved in language production and sensorimotor integration functions (Baldo et al., 2011; Itabashi et al., 2016).

**Figure 14:**
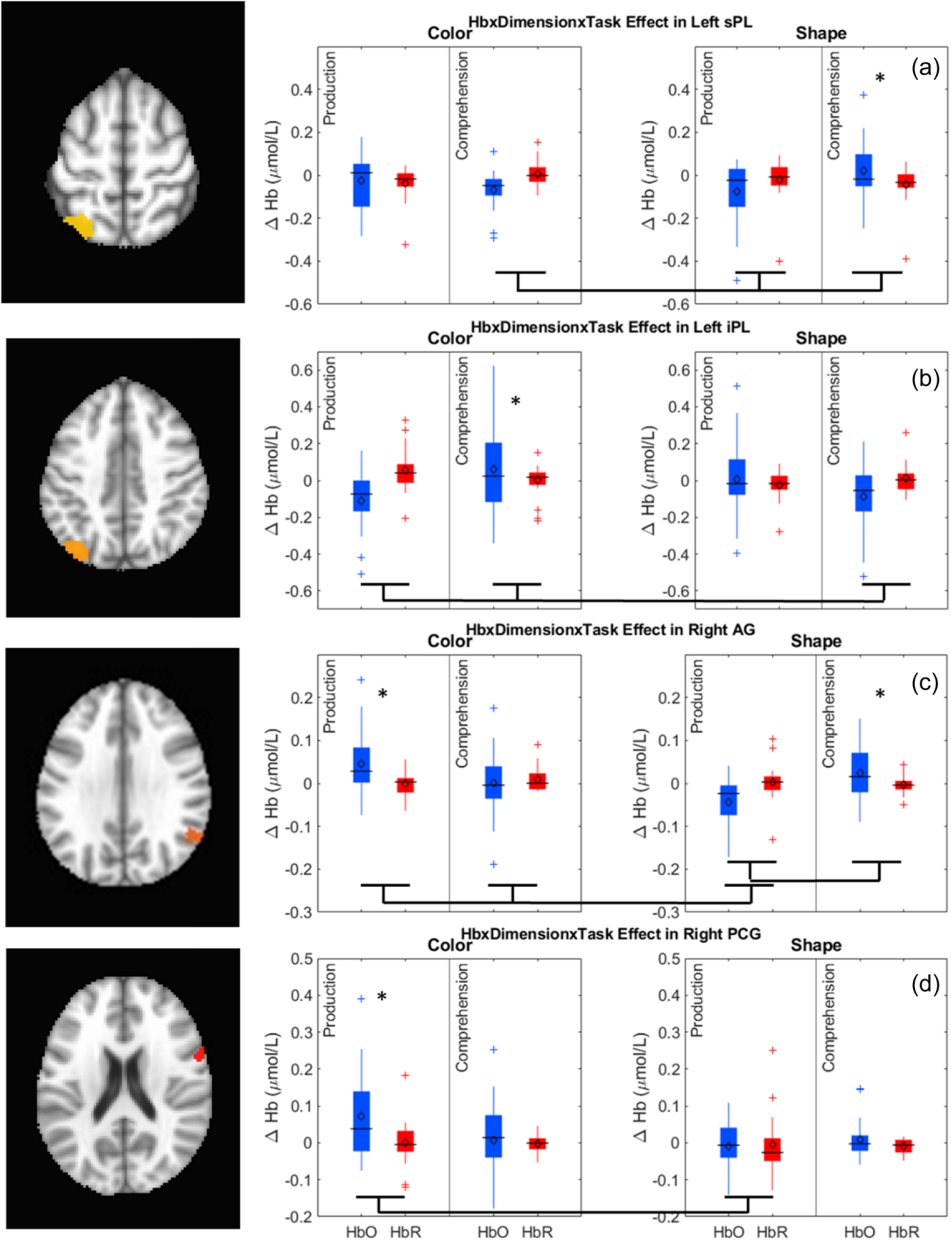
Clusters of neural activation in which an interaction between Hb, dimension, and task was observed. Left superior temporal lobule was more strongly activated during shape comprehension tasks when compared to color comprehension (a). Left inferior parietal lobule was more strongly activated during color comprehension tasks when compared to both color production and shape comprehension (b). Right angular gyrus was more active for shape comprehension compared to shape production and more strongly for color production when compared to both color comprehension and shape production. Lastly, right precentral gyrus was more strongly activated during color production tasks when compared to shape production.

We found an interaction between dimension, task, and Hb (Figure 14) in the left sPL, left iPL, right AG, and right PCG. Left sPL showed activation during shape comprehension (greater than shape production, *p* = .014, and color comprehension, *p* = .006), whereas left iPL showed activation during color comprehension (greater than color production, *p* = .016, and shape comprehension, *p* = .026) and shape production (greater than color production, *p* = .028). Right AG, located in iPL, showed activation during color production (greater than color comprehension, *p* = .039, and shape production, *p* < .001) and shape comprehension (greater than shape production, *p* = .008). Typical views on the neural networks involved in language emphasize frontal and temporal lobes; however, parietal cortex has long been considered to play a critical role in language (Coslett & Schwartz, 2018; Tabassi Mofrad & Schiller, 2020). The fact that shape and color comprehension were associated with distinct regions of parietal lobe highlights the nuanced nature of word‐feature associations in relation to the type of visual information being associated with labels. Recent reviews of the functional role of AG highlight a diverse set of functions implicated by AG (located in iPL), including multisensory integration, processing concepts, memory retrieval, and spatial attention (Chambers et al., 2004) in addition to its previously discussed role in language processing (Binder et al., 2009). Thus, it is not surprising that this region displayed a complex pattern of activation across our task types. Lastly, right PCG showed activation during color production (marginally greater than shape production, *p* = .051). As with the task × Hb interaction discussed above in left PCG, selective activation during production was observed in this task, specifically during color tasks in this case. It is noteworthy that color production engaged right PCG, which is not typically implicated in language function, but rather is associated with motor control (Nebel et al., 2014). The engagement of this region that we found during color production in our sample of young children may reflect a lack of lateralization of language function that emerges later in development.

**Figure 15:**
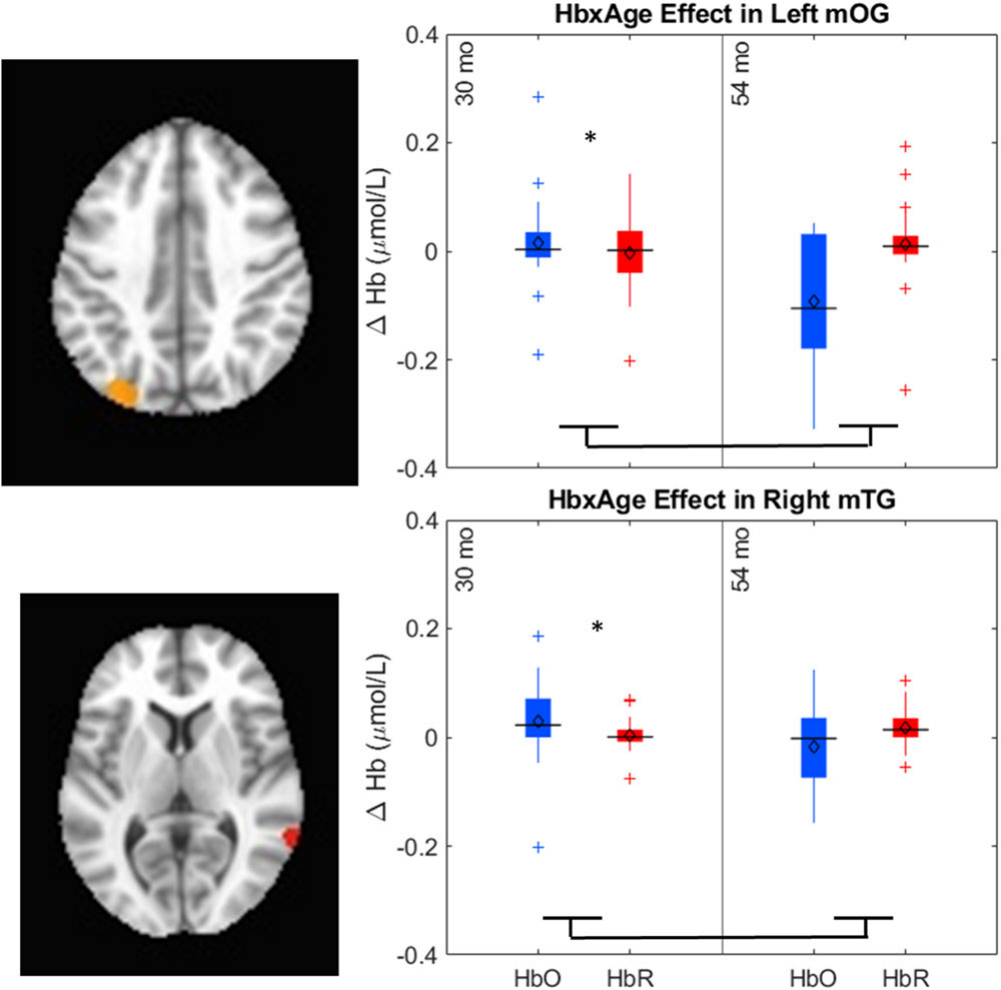
Clusters of neural activation in which and interaction between Hb and Age were observed. Both left middle occipital gyrus (top) and right middle temporal gyrus (bottom) were more strongly activated at 30 months when compared to 54 months.

Figure 15 displays an interaction between age and Hb found in left mOG and the right mTG in which activation was stronger at 30 months compared to 54 months (*p* = .005 and *p* = .019, respectively). As previously discussed, occipital regions are involved with processing low‐level visual stimuli (Vinberg & Grill‐Spector, 2008), which may reflect the fact that 30‐month‐olds, in general, have less robust feature label representations at this earlier point in development. In other words, if a child does not have adequate knowledge of their color and shape labels, then they will rely more heavily on processing low‐level stimulus information. At 54 months, children typically have better integrated associations between labels and visual features which relies more heavily on the parietal and frontal regions identified throughout this section. As previously mentioned, the left temporal gyrus is commonly associated with language processing (Margulies & Petrides, 2013; Noonan et al., 2013; Papeo et al., 2019; Sugimoto et al., 2023). The cluster revealed by this interaction is adjacent to the cluster identified task and Hb interaction above, suggesting that children in this sample were engaging a broader section of temporal cortex early in development. Stronger activation of this region at 30 months could indicate that at the younger age, children required more neural resources to build representations of label‐feature mappings. On the other hand, we could be seeing this effect because at 30 months, children simply required more verbal instruction and thus were exposed to more language while completing the task.

**Figure 16:**
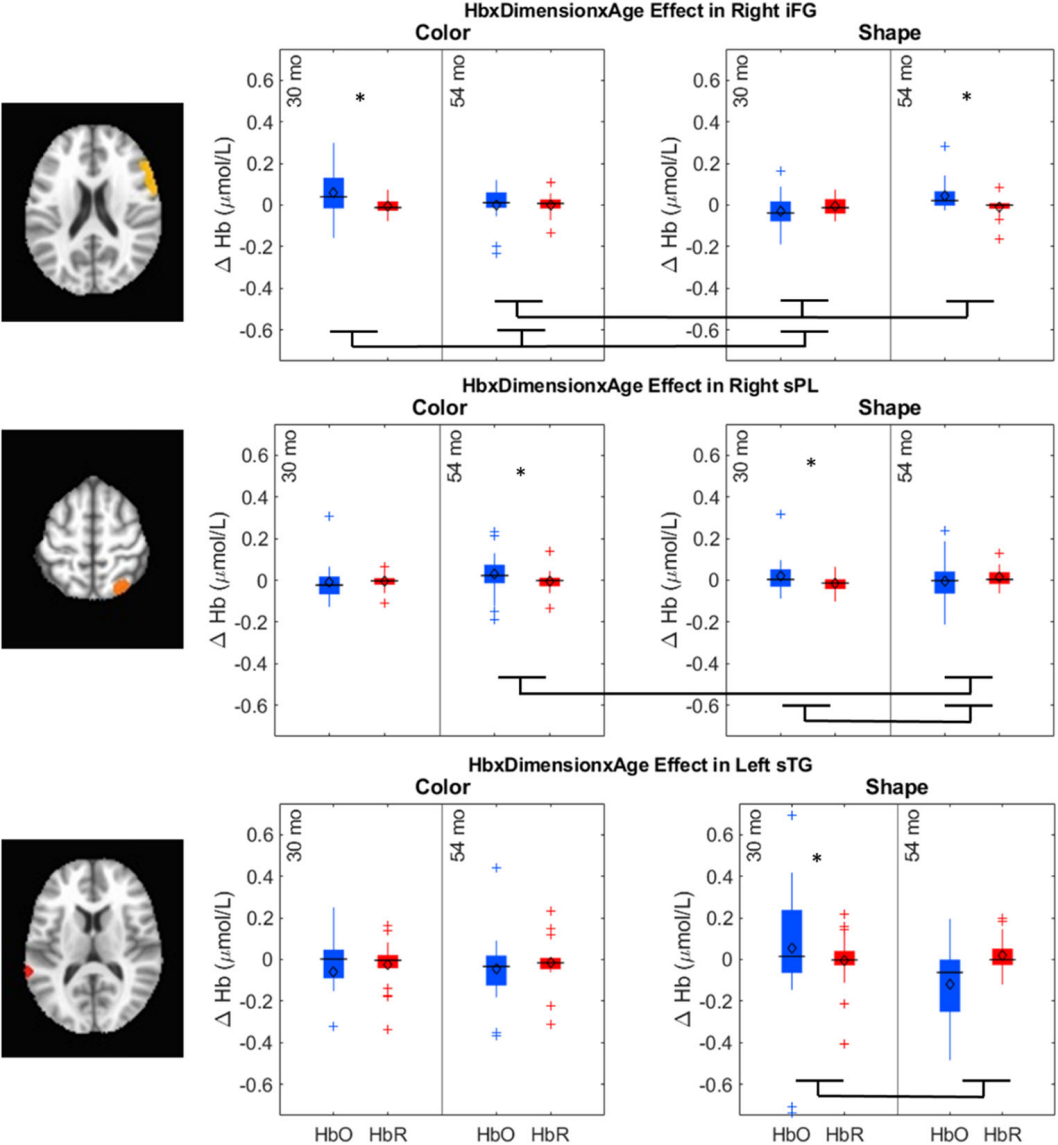
Clusters of neural activation in which an interaction between oxygenation, dimension, and age was observed. Activation in right inferior frontal gyrus (top) was greater for color tasks at 30 months when compared to color at 54 months and shape at 30 months but greater for shape tasks at 54 months when compared to shape tasks at 30 months and color tasks at 54 months. Activation of right superior parietal lobule (middle) was stronger for color tasks at 54 months when compared to shape tasks at 54 months but greater for shape tasks at 30 months when compared to shape tasks at 54 months. Activation in left superior temporal gyrus (bottom) was greater only during shape tasks at 30 months when compared to shape tasks at 54 months.

We found a three‐way interaction between age, dimension, and Hb in the right inferior frontal gyrus (iFG), right sPL, and left superior temporal gyrus (Figure 16). In right iFG, we observed activation during color trials at 30 months (compared to shape at 30 months, *p* = .019, and color at 54 months, *p* = .045) and activation during shape trials at 54 months (compared to color at 54 months, *p* = .023, and shape trials at 30 months, *p* = .010). In right sPL we observed activation during shape trials at 30 months (compared to color trials at 30 months, *p* = .045) and activation during color trials at 54 months (compared to shape trials at 54 months, *p* = .037). Thus, right iFG and right sPL displayed inverse relationships regarding their relative involvement with shape and color trials developmentally. These shifts could reflect coupling between these regions and relative shifting of the relative reliance on attention control to visual features in rIFG (Morton et al., 2009) and attentional control to spatial information (Corbetta et al., 1995). In left superior temporal gyrus, we also observed activation during shape trials at 30 months (compared to shape trials at 54 months, *p* = .036). Left superior temporal gyrus contains Wernicke's area and is associated with processing language and auditory stimuli (Ardila et al., 2016a; Bhaya‐Grossman & Chang, 2022; Graves et al., 2008; Yi et al., 2019). Thus, activation in this region during shape trials at 30 months of age may reflect the fact that children needed greater neural resources on shape trials due to shape labels being less familiar (Buss & Nikam, 2020).

**Figure 17:**
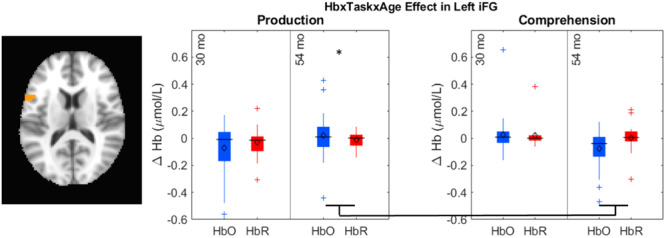
Cluster of neural activation in which an interaction between oxygenation, task, and age was observed in left inferior frontal gyrus. Activation was greater for production tasks at 54 months when compared to comprehension tasks at 54 months.

We found three‐way interactions between age, task, and Hb in left iFG and right sPL (Figure 17). In left iFG, we observed activation during the production task at 54 months (compared to the comprehension task at 54 months, *p* = .027). In general, left iFG is a region associated with cognitive control (Buss & Spencer, 2018; Moriguchi & Hiraki, 2009; Morton et al., 2009). Activation during production tasks likely reflects the additional cognitive demands required to use the labels “color” and “shape” to attend to specific feature dimension and then to resolve competition among the feature labels associated within each dimension (Lowery et al., 2022). In right sPL, this effect was driven by increases in HbR during the production task and was, thus, not indicative of activation.

**Figure 18:**
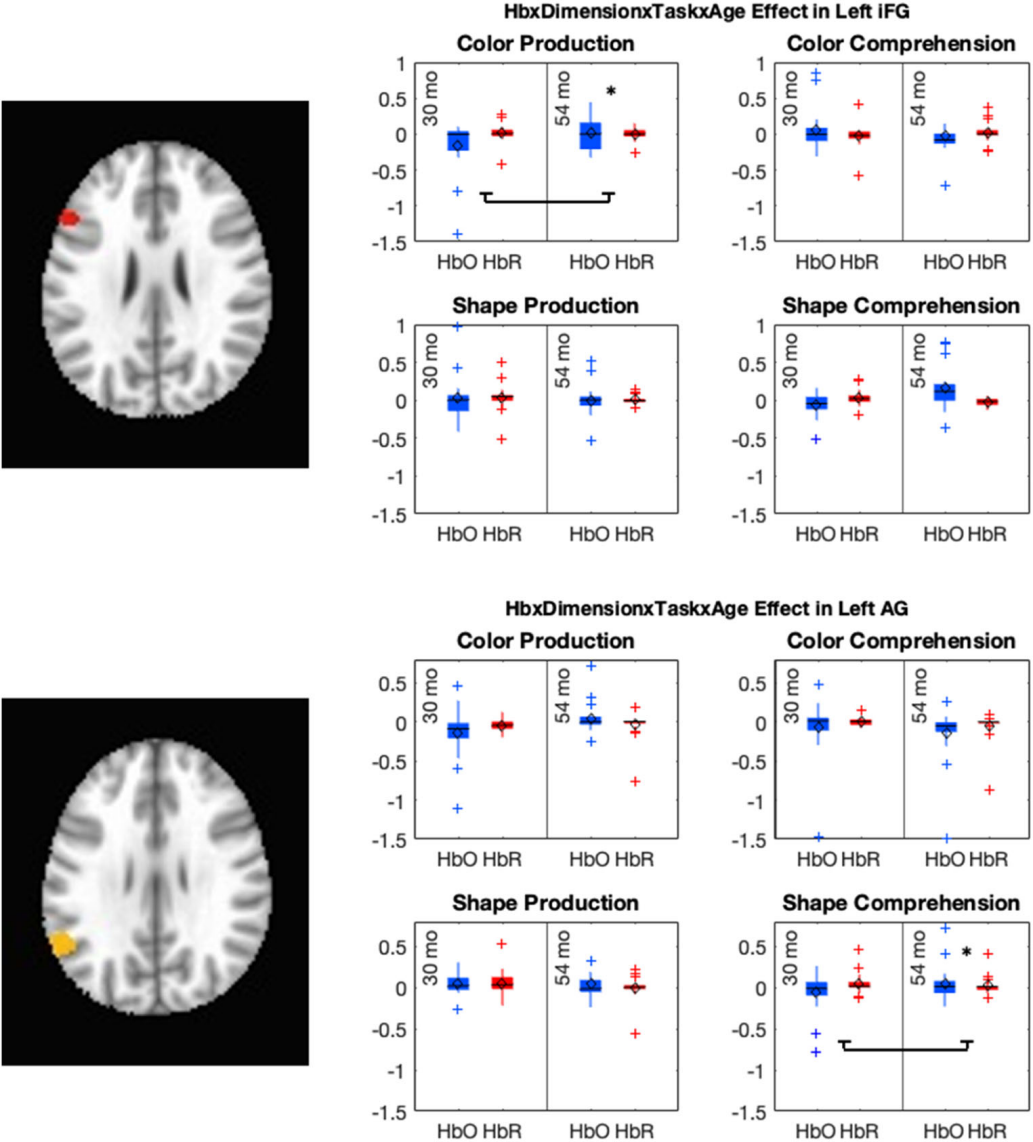
Cluster of activation in which an interaction between oxygenation, dimension, task, and age was observed in left inferior frontal gyrus (top) and left angular gyrus (bottom) such that inferior frontal gyrus was activated during color production trials at 54 months compared to 30 months and left angular gyrus was activated during shape comprehension tasks at 54 months compared to 30 months.

Lastly, we found four‐way interactions between dimension, task, age, and Hb in left AG, left iPL, and left iFG (Figure 18). For follow‐up tests in these clusters, we focused on comparing changes in activation within each task between 30 and 54 months. These follow‐ups revealed that the effect in left iPL was driven by deactivation during color production at 30 months. In left AG, activation was greater in our cohort of children at 54 months relative to 30 months during shape comprehension, *p* = .006. As mentioned previously, AG is implicated in the convergence of information, including comprehension of words, coming through both visual and auditory modalities. Children were thus better at integrating the semantic information coming in through the auditory modality and associating this with the visual information coming in through what they saw on the computer screen at 54 months. At 30 months, children did not have strong enough mappings between feature dimensions and their labels to integrate the two. In left iFG, activation was greater during color production at 54 months relative to 30 months, *p* = .027. It is worth noting that this cluster is adjacent to the cluster we found in left iFG in the Hb × task × age interaction, which was also implicated in production tasks. This 4‐way interaction suggests that the children in our cohort were using greater attentional resources during color production tasks. In general, performance on color label tasks was superior to that of shape label tasks, suggesting that the children were capable of using greater attention control during color labeling. Together, these findings speak to the nature of attentional resources needed for production trials.

### Conclusion

The findings presented here reflect the first investigation of dimensional labeling ability in children as young as 30 months of age, and many of the first neural findings for differences in neural activation between color and shape labeling abilities. Our results demonstrated a broad network of regions that were engaged during the comprehension and production tasks. First, we found that colors were processed in regions involved in higher level categorization in parietal cortex whereas shapes were processed in areas that are associated with lower‐level visual features in occipital cortex. Additionally, we found that producing dimensional labels more strongly engaged language‐specific areas in left PCG and right mTG relative to comprehending dimensional labels. Across parietal cortex, we also found a complex pattern of activation in relation to task and dimension. Left inferior parietal cortex showed activation during color comprehension and shape production whereas right AG showed activation during shape comprehension and color production. We also found aspects of parietal cortex were activated to specific task and dimension conjunctions: right superior parietal cortex was activated during shape comprehension trials and right PCG was activated during color production trials. These initial insights into the neural mechanisms of dimensional label learning provide more questions than answers.

More work is needed to tease apart the relative rate of label learning between shape and color dimensions in relation to the different task demands of production and comprehension tasks. As initially discussed by Sandhofer and Smith (1999), dimensional label learning requires a system of mappings. At one level, features and labels can be directly associated when learning labels such as “red” or “star.” In this case, features and labels can be reliably associated with each other across learning opportunities—when hearing “red” or “star” there is likely to be a red or star object present, respectively. When performing the comprehension task, children can use this direct mapping to guide spatial attention to select the appropriate target object from the array. Learning labels such as “color” or “shape,” however, is not as straightforward. Specifically, across learning opportunities, there will not be any visual stimulus that can be reliably associated with this category of broader labels. Instead, it seems that children may learn associations between labels and labels—even if not producing the correct label, children will reliably produce a color label during the production task (Sandhofer & Smith, 1999). For example, the label “color” can be associated with other labels such as “red” and “blue.” In the context of talking about colors, it is likely that these labels will be present together. Thus, using labels such as “color” and “shape” requires using multiple levels of associations. First, when asked, “What color is this?,” for example, children can engage associations between “color” and the array of specific color labels. These specific color labels will have associations with color features, which can then provide the basis for a form of dimensional attention so that children can select and produce the label associated with the presented feature. Together, our results showing that attentional control regions in iFG were engaged during the production task are consistent with the idea that the production task requires additional attentional control.

Interestingly, we also observed differences in activation between visual dimensions. The color tasks engaged regions associated with higher‐level categorization in parietal cortex whereas the shape tasks engaged regions associated with lower‐level feature processes in occipital cortex. These findings are consistent with a study that examined the relative frequency of the labels for the shape and color dimensions in the CHILDES database (Buss & Nikam, 2020). This study reported that labels for the visual dimension of shape were much less frequent that labels for the visual dimension of color. The relative frequencies of these labels may foster different rates of learning for these sets of labels. This study also showed that older children had significantly more difficulty using the label “shape” to guide attention than the label “color.” Specifically, 4‐year‐olds were administered the DCCS task in two conditions. In one condition, standard instructions were provided that indicated the dimension and the specific labels for the features within each dimension (e.g., “Sort by shape. Stars go here, and circles go there.”). In a second condition, children were only provided with dimensional labels during instruction (e.g., “Sort by shape. This shape goes here, and that shape goes there.”). Results showed that the 4‐year‐olds in our study performed well when switching to color regardless of instruction condition but perseverated at a significantly higher rate when switching to shape and instructed only with the label “shape.” Thus, the specific details of how children are exposed to labels differ between dimensions. This may impact the neural mechanisms supporting these label representations, as shown in our data, and may also impact their ability to use these labels to guide attention as shown by Buss and Nikam (2020).

Developmentally, we observed reductions in activation in left mOG and right mTG. These reductions in activation likely reflect a shift away from relying on low‐level feature information and an increase in lateralization of language functions, respectively. We also observed shifts in activation associated with the different dimensions. For color, 30‐month‐olds in our study activated right iFG but activated right sPL and left superior temporal gyrus at 54 months. For shape, 30‐month‐olds activated right sPL and left superior temporal gyrus but activated right iFG at 54 months. These shifts could reflect differential reliance on nodes of the dorsal attention network as a function of the changes in difficulty with these dimensions over development. By 54 months of age, the children in our study were nearly perfect in color label performance but still had room for improvement with shape labels. We also observed the emergence of activation in left iFG specifically on production trials. As discussed above, this likely reflects the additional demands on dimensional attention imposed by the production tasks. Lastly, we observed developmental differences in activation on specific task‐dimension conjunctions. At 54 months of age, children engaged left AG during shape comprehension trials and left iFG during color production trials. The left AG activation on shape comprehension could reflect greater reliance on binding between auditory labels and visual features that has been documented in this region. Activation in left iFG, which was adjacent to the cluster identified in the task × age × Hb interaction, could reflect greater skill in recruiting dimensional attention when prompted with the label “color” during color production tasks.

Together, these results provide the first window into the complex nature of neural mechanisms supporting dimensional label learning in early childhood. Not only are traditional language areas such as Broca's and Wernicke's areas implicated, but more generally multimodal association areas, broad regions across parietal cortex, and regions involved in attention control such as inferior frontal cortex and superior parietal cortex. Thus, these tasks not only provide a window into the associative learning process likely underlying dimensional label learning, but also the unique task demands as a function of comprehension and production and relative differences in difficulty based on the history of learning opportunities between shape and color labels.

## V Dimensional Understanding

In this chapter, we describe the methods and results for the dimensional understanding tasks administered at 30 and 54 months of age. This task required children to match items using a general understanding of similarity. No specific instructions were provided as to which dimension or features should match. Rather, children were shown two reference objects that were either the same shape or color and then an array of items to pick from to determine if they could generalize that similarity to another object. The tasks we administered were modeled after tasks that have been previously used to assess children's dimensional understanding in relation to label knowledge for color (Sandhofer & Smith, 1999) and shape (Verdine et al., 2016). Here, the timing, sequence of stimulus presentation, and number of trials has been optimized for use with functional neuroimaging. As with the dimensional label knowledge tasks, the matching tasks have not been administered in prior research to assess both color and shape dimensional understanding within the same group of children.

### Methods

**Figure 19:**
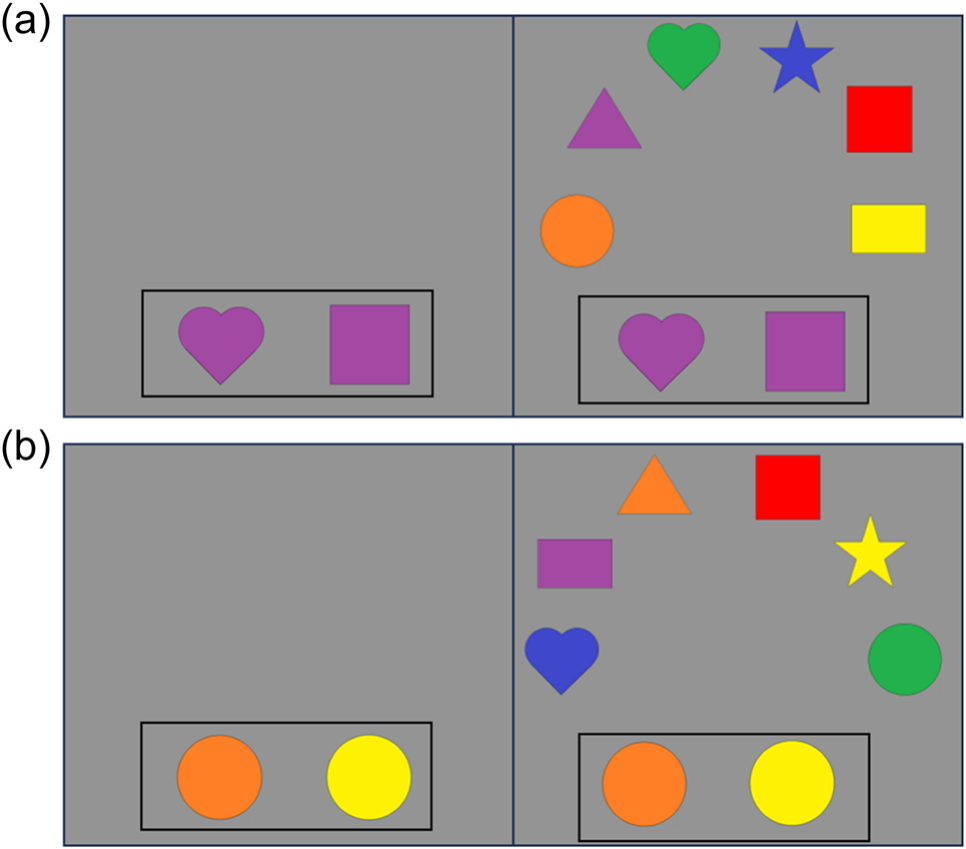
The matching tasks for a color trial (a) and a shape trial (b). First, two reference objects were shown at the bottom (left) and children were told, “See how these two are the same?” Next, an array of six objects were presented in a semicircle above the reference objects (right) and children were told to “Find one that is the same like these.” Only one item matched each feature present on the reference objects.

The dimensional understanding task used the same shapes (circle, square, rectangle, triangle, heart, star) and colors (red, blue, green, yellow, orange, purple) as the dimensional label comprehension and production tasks presented in Chapter IV. In the dimensional understanding task (see Figure 19), two stimuli matching in one feature dimension first appeared inside a box at the bottom of the screen, accompanied with the verbal prompt, “Do you see how these are the same?” Next, an array of six stimuli (every shape and color was presented and no features repeated) was presented along with the verbal instruction “find the one here (gesture to the array) that is the same as these (gesture to the stimuli in the box).” Children indicated a response by touching the selected stimulus on the touchscreen. Behavioral data were analyzed using a 2 (Dimension: color, shape) × 2 (Age: 30 mo, 54 mo) repeated measures ANOVA.

Neural data were processed as described in Chapter II. We analyzed group data with a 2 (Dimension: color, shape) × 2 (Age: 30 months, 54 months) × 2 (Oxy: HbO, HbR) ANOVA using 3dMVM (Chen et al., 2014). Family‐wise error calculations (described in Chapter II) indicated a cluster threshold of 56 mm^3^ was needed for *α* < .05 with voxel‐wise *p* < .05. Average *β* values of HbO and HbR were extracted from clusters that satisfied these threshold criteria and follow‐up tests were performed using SPSS (IBM, version 29). To reduce the dimensionality of the data and simplify the follow‐up tests, we first computed an *activation* variable as the difference between HbO and HbR. In general, functional brain activation is reflected by an increase in HbO relative to HbR levels.

### Behavioral Results

**Figure 20:**
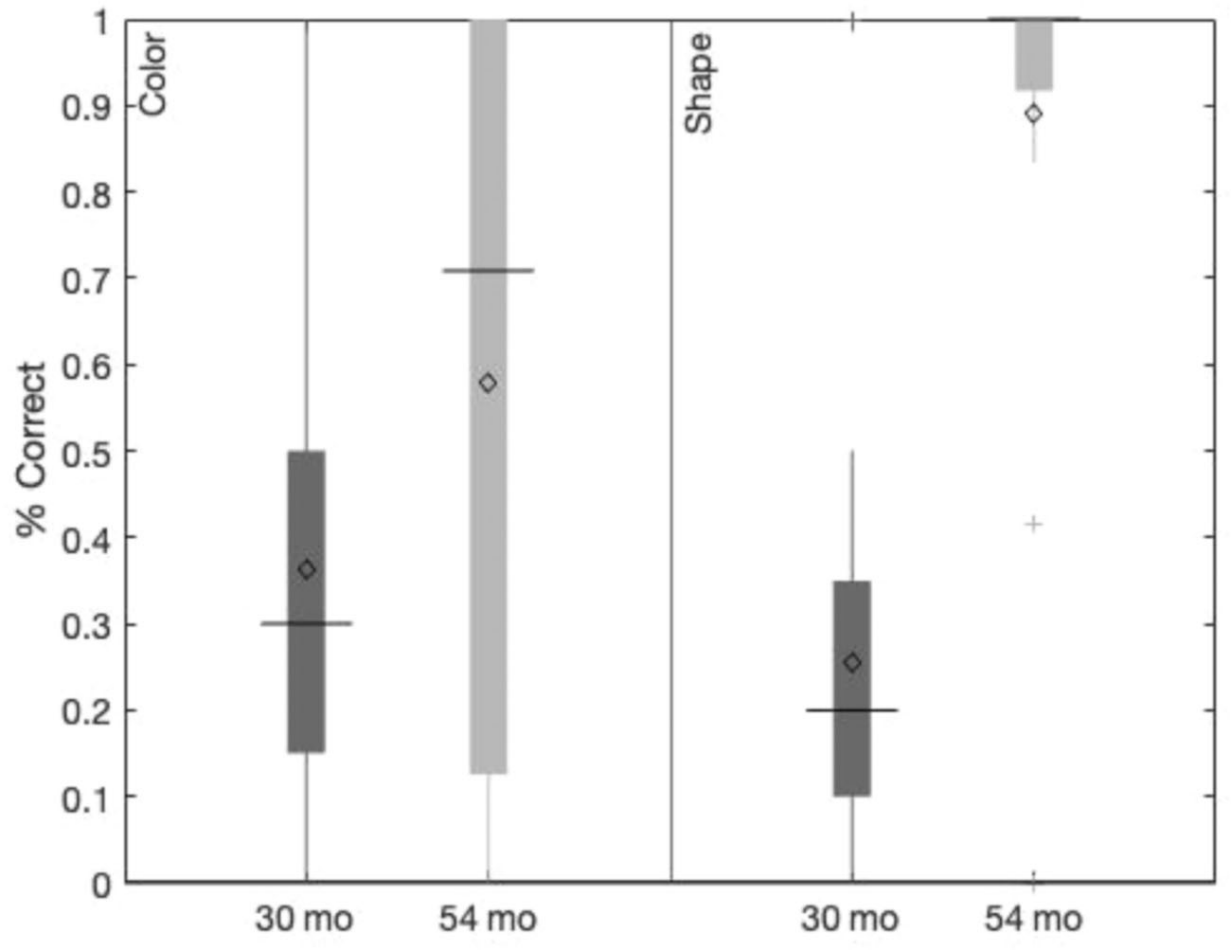
Box plots of accuracy data are plotted for color (left) and shape (right) trials for 30 months (dark grey) and 54 months (light grey). Means are marked by diamond, median values are marked by horizontal lines, standard deviation is marked by thick bars, confidence intervals representing 95% of distribution are marked by thin bars, and outliers are marked with crosses (+).

Behavioral accuracy is plotted in Figure 20. We found a main effect of age, *F*(1,19) = 50.991, *p* < .001, *η* = .729. Children were more accurate at 54 months (*M* = .735) than at 30 months (*M* = .308). There was no effect of dimension, but there was an interaction between age and dimension, *F*(1,19) = 8.760, *p* = .008, *η* = .316. Children were significantly better at shape matching (*M* = .892) than color matching (*M* = .579) at 54 months. Thus, although children struggled with both dimensions at 30 months of age (but were still above chance, which was 1/6), performance significantly improved, especially for the shape dimension, by 54 months of age.

### Neural Results

Analyses of the neural data revealed 13 clusters that showed significant effects during the dimensional understanding tasks. However, two of these regions showed only deactivation (HbO < 0) and were not included in further analyses. Table 3 lists the brain regions, coordinates, cluster volume, and the observed effects for each cluster.

**Table 3:** Clusters Identified in Analysis of Functional Near‐Infrared Spectroscopy Data From Matching Task

*Neural Regions of Activation Associated With the Matching Task*							
		MNI Coordinates					
Effect	Region	*X*	*Y*	*Z*	Volume (mm^3^)	GES	Pattern
Hb	Right iFG	−52.9	−28.2	10.8	409	.064	HbO > HbR
	Left iFG	51.5	−17.3	23.9	344	.068	HbR > HbO
Hb × Dim	Left iPL	29.3	75.6	45.0	191	.052	Color > Shape
	Right iFG	−54.3	−16.5	−5.0	143	.017	Color > Shape
	Right sTG	−64.4	34.4	8.6	122	.028	Shape > Color
	Left sPL	31.5	58.1	64.2	99	.019	Deactivation
	Left mTG	64.7	33.4	6.0	57	.020	Shape > Color
Hb × Age	Left AG	53.4	57.7	32.6	235	.056	54 > 30 months
	Right sOG	−28.5	77.8	45.1	105	.053	30 > 54 months
	Left PoCG	62.1	15.9	20.5	64	.032	30 > 54 months
	Right AG	−50.4	59.0	38.1	57	.045	54 > 30 months
Hb × Dim × Age	Left AG	42.5	60.8	46.1	326	.029	30 months Color:
							HbO > HbR
	Right iFG	−56.1	−11.3	−2.1	67	.025	30 months:
							Color > Shape

*Note*. AG = angular gyrus; HbO = oxygenated hemoglobin; HbR = deoxygenated hemoglobin; iFG = inferior frontal gyrus; iPL = inferior parietal lobule; mOG = middle occipital gyrus; mTG = middle temporal gyrus; PoCG = postcentral gyrus; sOG = superior occipital gyrus; sPL = superior parietal; sTG = superior temporal gyrus.

**Figure 21:**
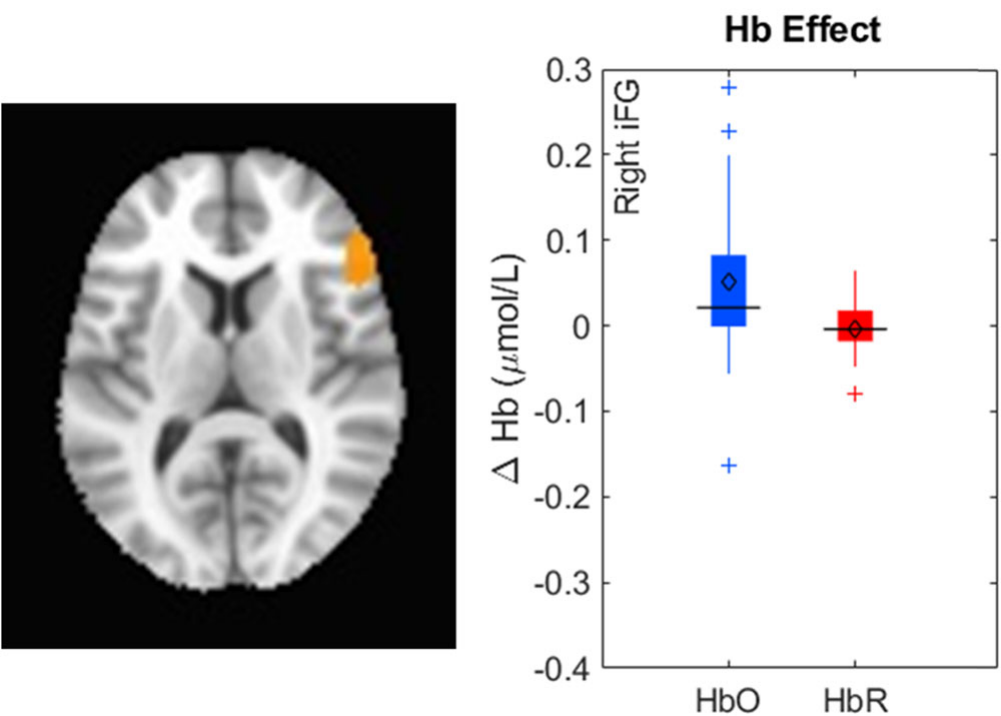
Clusters of neural activation in which oxygenated hemoglobin is significantly higher than deoxygenated hemoglobin. Right inferior frontal gyrus displays greater activation during the task when collapsing by every age/condition.

First, we found a main effect of Hb in right iFG, suggesting that this region was activated to the general demands of the task (Figure 21). An additional cluster was observed in left iFG, but this effect was driven by deactivation.

**Figure 22:**
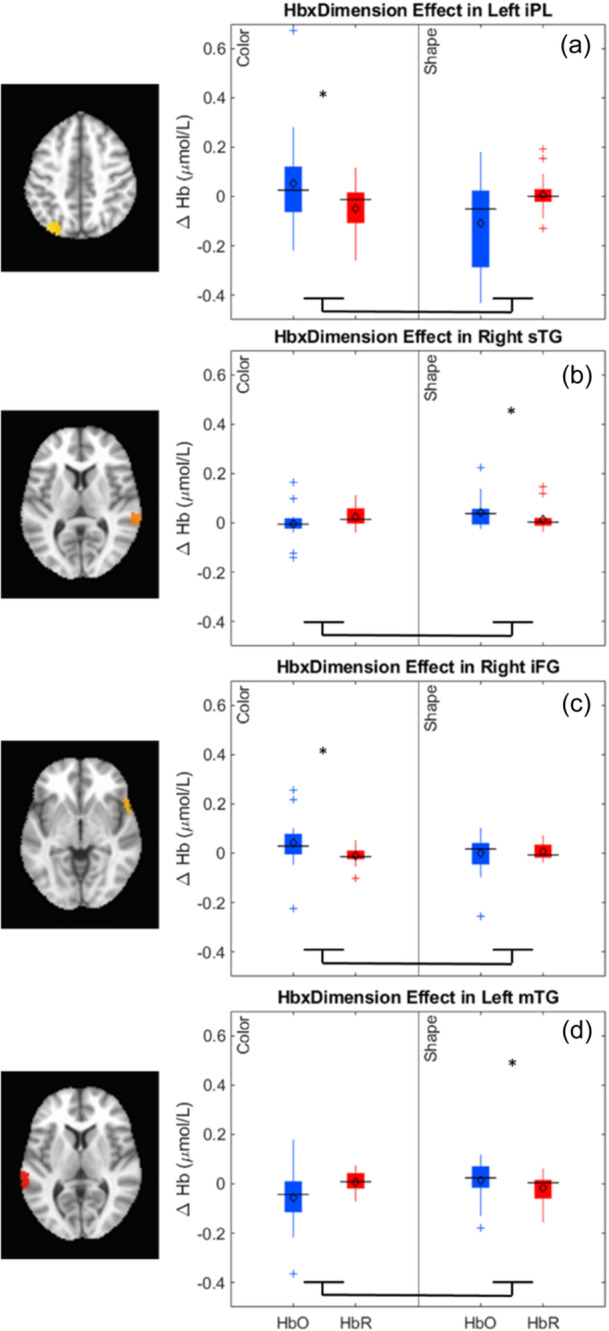
Clusters of neural activation in which an interaction between oxygenation and Dimension was observed. Left inferior parietal (a) and right inferior frontal gyrus (c) are activated during color tasks when compared to shape tasks, while right superior temporal gyrus (b) and left middle temporal gyrus (d) are activated during shape tasks when compared to color.

We found an interaction between dimension and Hb in both left iPL (*p* = .010) and right iFG (*p* = .015). These results are displayed in Figure 22. In both clusters, activation was stronger during the color task compared to the shape task. Additional clusters were found in right superior temporal gyrus (*p* = .009) and left mTG (*p* = .015). In these clusters, activation was greater for shape trials compared to color.

These different networks of activation could indicate different attentional strategies being used across dimensions. The engagement of frontal‐parietal cortex during the color task could indicate that the children were using a strategy that involves spatial attention and inhibition (Szczepanski et al., 2014, 2013). On the other hand, engagement of temporal cortex during the shape tasks could indicate that the children had a stronger mapping between the shape dimension and general language processes. This may be due to the influence of the shape bias, where children in general are more likely to attend to shape when categorizing objects. This interpretation may appear to conflict with the observation that the children in our task performed more poorly on the shape comprehension and production tasks (Chapter IV). However, the children's difficulty with these dimensional label tasks could be due to slower development of (due to fewer opportunities to learn) the mapping between specific shape labels and shape features (Buss & Nikam, 2020), despite an overall stronger association between language processing and the shape dimension. One final cluster was found in left sPL, but follow up analyses revealed that this relationship was driven by deactivation.

**Figure 23:**
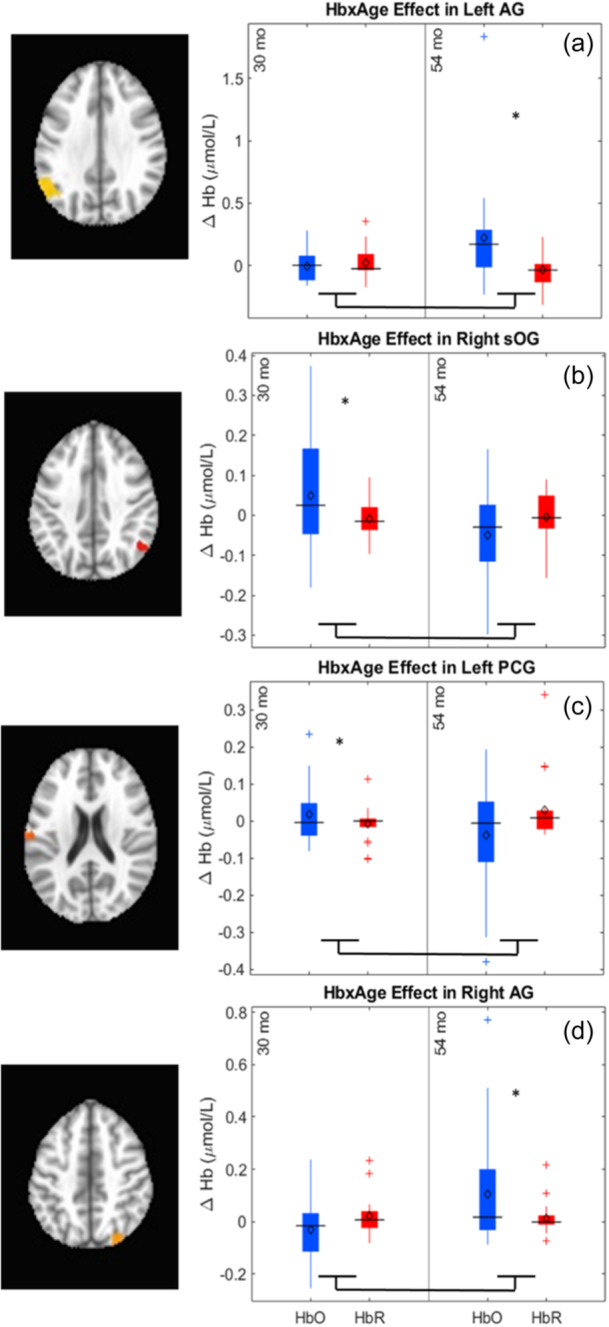
Clusters of neural activation in which an interaction between oxygenation and age was observed. Activation in both left (a) and right angular gyrus (d) was stronger at 54 months compared to 30 months. Activation in superior occipital gyrus (b) and left precentral gyrus (c) was stronger at 30 months when compared to 54 months.

Interactions between Hb and age (Figure 23) were found in bilateral AG (AG) where the children in our study showed greater activation at 54 months (left *p* = .026; right *p* = .035). This finding coincides with some of our previous interpretations as AG is involved in semantic understanding (Davey et al., 2015; Seghier, 2013). We identified an additional cluster in right superior occipital gyrus (sOG) where activation was greater at 30 months (*p* = .010). As this region is involved in lower‐level visual processing, it is not surprising that the 30‐month‐olds in our study, who did not have strong categorical representation of feature dimensions, showed stronger activation in regions associated with lower‐level feature processing. One final cluster was found in left postcentral gyrus (PoCG) where activation was greater at 30 months (*p* = .019). While this region is a part of the somatosensory cortex, it is also often activated in language tasks (Correia et al., 2014; Tremblay & Small, 2011). There is also evidence that lesion to this area leads to speech disorders (Mitani & Sakurai, 2024). It is likely that the children activated left PoCG more at 30 months than at 54 months because of the inevitable differences in task administration. At 30 months, the children in our study required more repetition of instruction, and were thus exposed to more language during the task.

**Figure 24:**
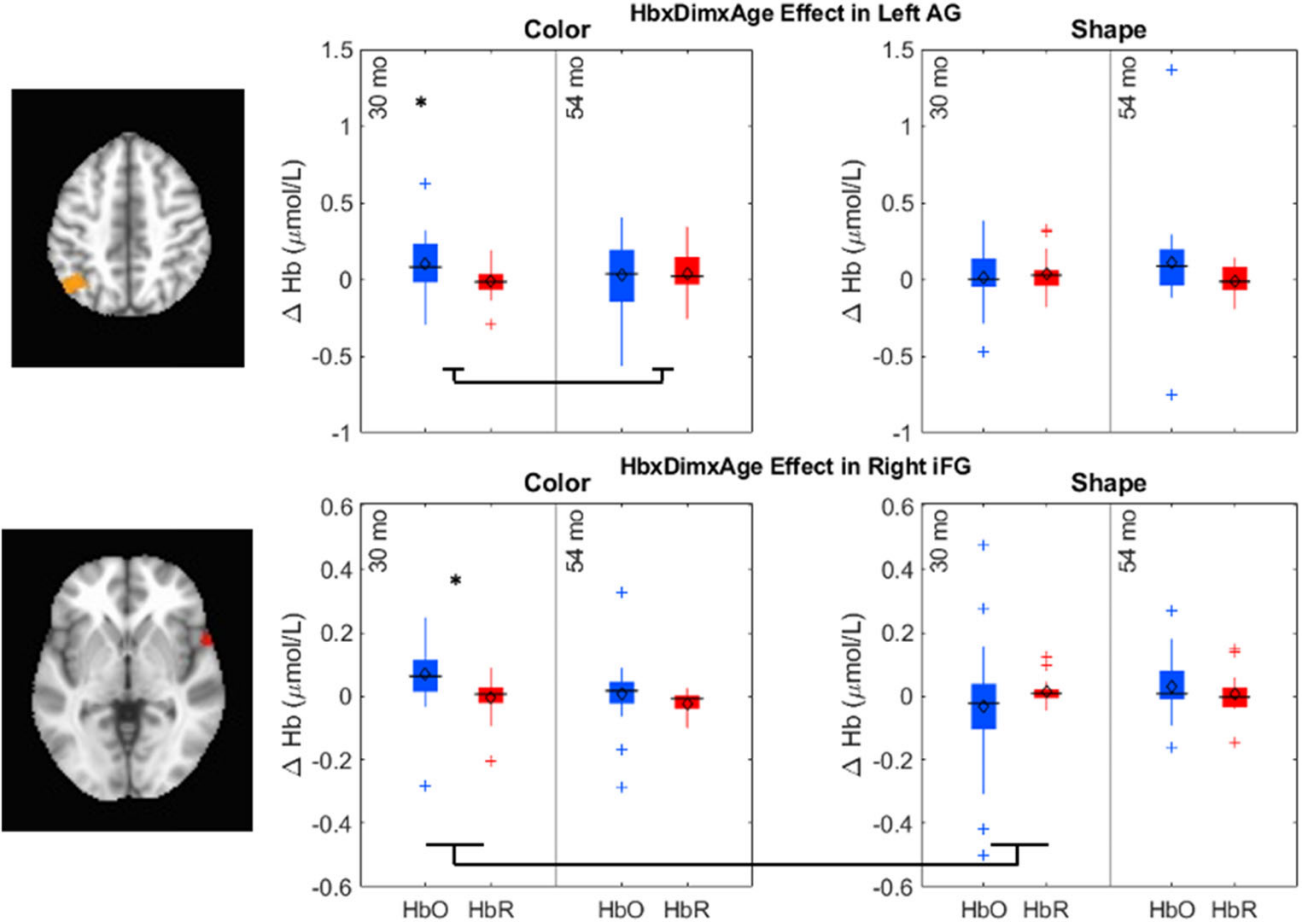
Clusters of neural activation in which an interaction between oxygenation, dimension, and age was observed. Activation in left angular gyrus was stronger during color tasks at 30 months when compared to 54 months whereas activation of right inferior frontal gyrus was stronger during color tasks at 30 months when compared to shape tasks at 30 months.

Lastly, we found a three‐way interaction between age, Hb, and dimension in right iFG (Figure 24) where activation was greater at 30 months during color trials compared to shape (*p* = .018). As mentioned previously, this region is associated with the integration of information from many modalities, such as multiple sensory inputs and previous experience. In accordance with this, a study by Ekerdt et al. (2020) found that changes in white matter in this region were associated with word learning in preschoolers. It is possible that at 30 months children were still consolidating label‐feature associations whereas 54‐month‐olds were already largely proficient color and shape labelers. An additional cluster was found in left AG; however, no comparisons of our activation metric were significantly different. Breaking data down by chromophore, we found that HbO was greater than HbR during the color task at 30 months of age. Note that these clusters were adjacent to clusters showing an effect of Hb (right iFG) and an interaction between age and Hb (left AG), suggesting that activation in these regions became more strongly localized developmentally, and that neural resources were less coordinated for color in particular in the context of this task.

### Conclusion

In this chapter we provided the first comparison of dimensional understanding development across shape and color dimensions. Moreover, we also provided the first examination of the neural mechanisms supporting dimensional understanding development. First, our results showed asymmetrical development of the ability to attend to the dimensions of shape and color. While the children in our study improved on both the shape and color matching tasks from 30 to 54 months of age, they were significantly more accurate on the shape matching task relative to the color matching task at 54 months. This bias in attention to shape is consistent with the overall attention to shape that children typically display in early childhood in the context of novel label learning and categorization. This bias appears in English speaking populations as early as age two (Hupp, 2015) and becomes rigidly established by age 3 (Samuelson et al., 2008). Although the matching tasks we used did not involve generalizing a novel label, these tasks did present children with an ambiguous context in which they may have been more capable of deploying attentional resources to the shape dimension. In other words, the improved accuracy of our cohort on shape trials at 54 months of age may reflect children's general predisposition to attend to the shape dimension over any other feature dimension.

The original studies that the matching task was modeled after (Sandhofer & Smith, 1999; Verdine et al., 2016) suggested that these tasks tapped into a form of dimensional understanding that is supported by a system of mappings between visual features and dimensional labels. Our results, however, suggest that the relationship between dimensional label learning and dimensional understanding may be more complex. Specifically, the children in our study performed better on shape trials in the matching task but performed better on color trials during the dimensional label tasks (Chapter IV). These conflicting findings may reflect an inherent difference in the two tasks. On the label tasks, children were directly provided with verbal input as to which features or dimensions are being probed. Previous research has shown that feature labels for the color dimension are more common in children's language input than labels for the shape dimension (Buss & Nikam, 2020). This means that specific associations between features and labels may form more readily within the color dimension than the shape dimension. As previously mentioned, children are also developing more generalized attentional strategies as they learn how aspects of objects are important for categorizing or grouping objects together. Thus, in the ambiguous context of the matching task, children may fall back on such a strategy when making decision about how to generalize the similarity of the reference objects. Together, our data suggest that children's learning of feature/label bindings interacts with more general attentional strategies that children develop.

Neurally, we found that the left iFG was generally engaged by the tasks. We also identified a frontal‐parietal network (left inferior parietal cortex and right iFG) that was engaged specifically during the color task. In contrast, a network of regions in temporal lobe (right superior temporal gyrus and left mTG) were activated more strongly for the shape task. These different networks of activation could indicate different attentional strategies being used across dimensions. The engagement of frontal‐temporal cortex during the color task could indicate that children used a strategy that involves spatial attention and inhibition, which would not be helpful for the goal of matching by a particular feature dimension. On the other hand, engagement of temporal cortex during the shape tasks could indicate that children had a stronger mapping between the shape dimension and general language processes. This mapping to language processes may provide the bias to attend to shape in ambiguous categorization tasks.

Developmentally, we found that the children in our study activated right superior occipital cortex at 30 months but activated bilateral AG at 54 months. This pattern of results reveals a shift from lower‐level visual processing (occipital cortex) to higher level processing. In particular, AG has been associated with language processing and strategic attention (Seghier, 2013). Lastly, we also found that children engaged more regions of cortex during color trials at 30 months of age. This may be due to the overall challenge of extracting similarity along the color dimension in the ambiguous context provided by the matching task. This may also reflect less organized neural mechanisms supporting attention to color early in development.

In summary, these results shed important new light on the development of dimensional understanding and dimensional attention. Our results showed that the children in this study developed attention to shape more readily than to color. These results are particularly striking given the overall proficiency that children displayed when using attention that is explicitly instructed. For example, the children in our study were better at color labels tasks than shape label tasks. These differences in the deployment of attention are likely due to the implicit nature of the matching tasks which required the children to infer the relevant dimension based on the configuration of stimuli in the task. Our results also highlight the critical role of language development in dimensional understanding. Specifically, we observed age‐related increases in activation in bilateral AG. Moreover, the accelerated development in performance on the shape task was associated with activation in bilateral temporal cortex during shape trials.

## VI Response Selection and Stimulus‐Response Conflict Resolution

In this chapter, we describe the methods and results for the Simon task administered at both 30 and 54 months of age. The Simon is a response selection and inhibition task and is considered a measure of simple EF, meaning even 30‐month‐olds are typically able to perform it. Children were required to make a categorical response via a button press to two objects based on a defined feature of the objects (in the case of the present study, whether the image is of a cat or a dog) while ignoring the lateralized placement of the stimulus which is irrelevant for response selection. In our task, the image of a cat corresponds to a leftward button press and the image of a dog represents a rightward press. If the image of a cat appeared on the left side of the screen, this was considered a congruent trial as the spatial location information corresponds with the location of the correct button. If the image of a cat appeared on the right side of the screen, however, this was considered an incongruent trial as the spatial location information conflicted with the location of the correct button. During some trials, the image appeared in the center of the screen. As this trial type confers no spatial location information, this was considered a neutral trial.

### Methods

The Simon task requires children to make a lateralized (left/right) button press to two different categories–in the task for this study a picture of either a cat or a dog–while ignoring the spatial location of the stimuli. The children in our study received 64 total trials where an image of an animal appeared on the screen and, based on the animal present, made a response on a button box. Prior to completing the task, children were shown two example trials performed by the experimenter and instructed, “In this game, you are going to see puppies and kittens and it's your job to feed them. These animals are very hungry but remember: puppies don't like kitty food and kitties don't like puppy food. When you see a kitty, you will push this button to feed it (*gesture to button on the left)* and when you see a puppy, you will push this button to feed it (*gesture to button on the right)*. I am going to do the first two for you and then it will be your turn.”

**Figure 25:**
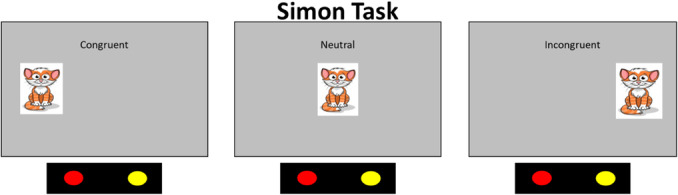
Methods image describing the different Simon conditions. Below is a visualization of a button box where the red button corresponds to the cat category and the yellow button corresponds to dog.

Children received three different randomly permuted conditions: neutral, congruent, and incongruent (see Figure 25). If the child pressed the correct button, an audio clip of the word “yummy!” was played, otherwise the image of the animal was removed from the screen. If no button was pressed after three seconds of stimulus presentation, the image disappeared, and the trial was reinitiated. If the child did not press any button after reinitiating the trial three times, the trial was canceled and the experiment moved onto the next trial.

We analyzed behavioral accuracy and reaction time using separate 3 (Condition: Neutral, Congruent, Incongruent) × 2 (Age: 30 months, 54 months) repeated measures ANOVAs. Neural data were processed as described in Chapter III. Group data were analyzed with a 3 (Condition: Neutral, Congruent, Incongruent) × 2 (Age: 30 months, 54 months) × 2 (Oxy: HbO, HbR) ANOVA using 3dMVM (Chen et al., 2014). Family‐wise error calculations (described in Chapter III) indicated a cluster threshold of 57 mm^3^ was needed for *α* < .05 with voxel‐wise *p* < .05. Average beta values of HbO and HbR were extracted from clusters that satisfied these threshold criteria and follow‐up tests were performed using SPSS (IBM, version 29). To reduce the dimensionality of the data and simplify the follow‐up tests, we first computed an *activation* variable as the difference between HbO and HbR. In general, functional brain activation is reflected by an increase in HbO relative to HbR levels.

### Behavioral Results

**Figure 26:**
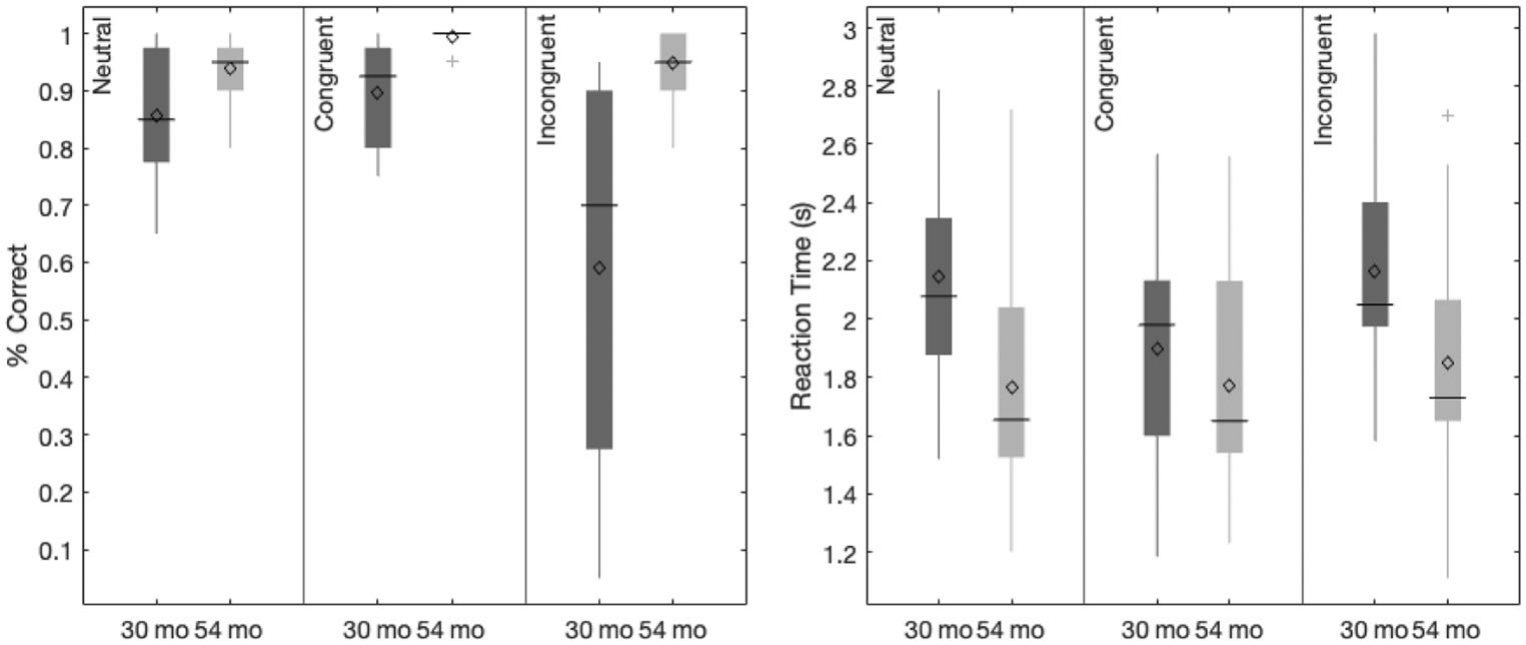
Graph depicting results for accuracy (left) and reaction time (right) during the Simon task, with 30‐month‐olds displayed in darker gray and 54‐month‐olds in a lighter gray.

Behavioral results are depicted in Figure 26. In the accuracy data, we found a main effect for condition, *F*(2,19) = 15.104, *p* = <.001, *η* = .443. Follow‐up comparisons showed that the children in the study were more accurate in the congruent condition (*M* = .945) than both the neutral condition (*M* = .901; *p* = .008) and the incongruent condition (*M* = .767; *p* < .001). Children were also more accurate on the neutral condition than the incongruent condition (*p* = .011). An additional main effect was found for age, *F*(1,19) = 41.692, *p* = <.001, *η* = .687, where children performed with higher accuracy at 54 months (*M* = .961) compared to 30 months (*M* = .781). There was also a significant interaction between condition and age, *F*(2,19) = 14.575, *p* = <.001, *η* = .434. Follow‐up comparisons showed that accuracy on neutral (*M* = .855) and congruent (*M* = .898) conditions did not differ at 30 months of age (*p* = .190), but the incongruent (*M* = .590) condition was lower than both the neutral (*p* = .004) and congruent (*p* = .002) conditions. At 54 months, the neutral (*M* = .946) and incongruent (*M* = .944) conditions did not differ (*p* = 1.000), but the congruent condition (*M* = .992) was higher than both the neutral (*p* = .004) and incongruent conditions (*p* = .006).

In the reaction time data, we found a main effect for age, *F*(1,19) = 14.305, *p* = .001, *η* = .430, indicating that children performed more quickly at 54 months (*M* = 1,721 ms) compared to 30 months (*M* = 2,113 ms). We also found a main effect of condition, *F*(2,19) = 14.887, *p* < .001, *η* = .439. Follow‐up analyses showed that reaction time was faster on congruent trials (*M* = 1,807 ms) compared to neutral (*M* = 1,932 ms; *p* = .013) and incongruent (*M* = 2,014 ms; *p* < .001). Reaction time did not differ between neutral and incongruent trials (*p* = .174). Lastly, there was a significant interaction between condition and age, *F*(2,19) = 8.270, *p* = .001, *η* = .303. At 30 months, reaction time did not differ between neutral and incongruent trials (*p* = 1.000), but was faster on congruent trials compared to both neutral and incongruent trials (both *p* < .001). At 54 months, reaction time did not differ between any conditions (all *p* > .136).

The Simon effect is traditionally observed in adults as an increase in reaction time on incongruent trials (Simon, 1990) due to the need to resolve the spatial conflict between the position of the stimulus and the position of the button‐press response. We did not observe this pattern in our cohort of children at either age. Instead, the spatial location of the stimulus strongly influenced the children at 30 months in terms of both accuracy and reaction time: children in our study were fastest on congruent trials and most error‐prone on incongruent trials. At 54 months, children performed with highest accuracy on congruent trials, but reaction time did not differ between any conditions. Children may not have shown a significant slow‐down on incongruent trials because their reaction times were already slow (>1,500 ms in all conditions), likely due to lack of appropriate dexterity needed for efficient button pressing.

### Neural Results

**Figure 27:**
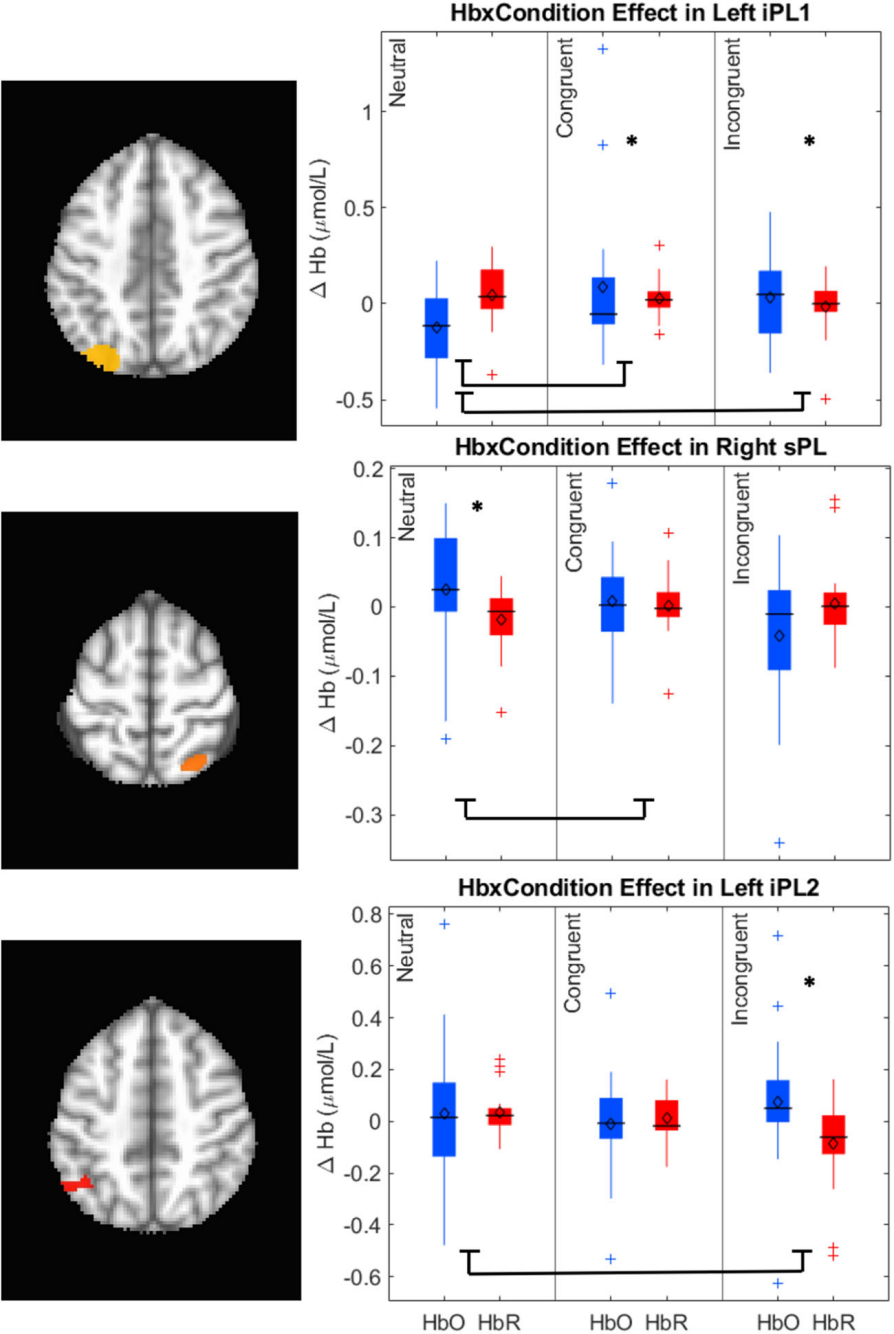
Clusters of neural activation in which an interaction between oxygenation and condition was observed. One region of left inferior parietal lobule (top) showed activation in both incongruent and congruent trials when compared to neutral trials. Activation in right superior parietal lobule (middle) was stronger during neutral trials compared to congruent trials. Activation in a different region of left inferior parietal lobule (bottom) was stronger for incongruent trials compared to neutral trials.

Table 4 displays the observed effects, brain regions of the clusters, cluster coordinates, cluster volumes, and patterns of activation. First, we observed effects of Hb bilaterally in the iFG and the left AG but follow up analyses revealed that these effects were driven by deactivation. Interactions between condition and Hb were found in two clusters of left iPL and one cluster in right sPL (Figure 27). In the first cluster of left iPL, activation was weaker on neutral trials compared to incongruent (*p* = .002) and congruent (*p* = .013) trials. Children showed less activation during neutral trials perhaps because these trials provide no spatial location information that overlaps with the response locations. Thus, this region may be responding to the lateralized presentation of the stimulus. In a lower region of left iPL, activation during incongruent trials was stronger compared to congruent (*p* = .023) and neutral (*p* = .041) trials, suggesting that this region is involved in the inhibition of spatial location information while completing the task. iPL is a region with complex organization and many subdivisions (Igelström & Graziano, 2017). Relevant for the consideration of performance on the Simon task, it is a region that connects Broca's area and Wernicke's area (Ardila, 2010) and is involved in bottom‐up attentional processes (Corbetta et al., 2000). Thus, this region may be involved in simple labeling of the object identity needed for response selection and registering the spatial location of the stimulus in a bottom‐up fashion. Of note, fMRI research with adults has shown that this region responds to incongruent trials as well (Kerns, 2006). In contrast, an Hb × condition interaction in right sPL showed greater activation for neutral trials compared to congruent (*p* = .041) and incongruent (*p* = .006) trials. This region has been previously discussed for its implication in spatial attention (Corbetta et al., 1995), and thus could reflect that spatial attention does not need to shift laterally in the neutral condition.

**Table 4:** Clusters Identified in Analysis of Functional Near‐Infrared Spectroscopy Data From Simon Task

*Neural Regions of Activation Associated With the Simon Task*							
		MNI Coordinates					
Effects	Region	*X*	*Y*	*Z*	Volume (mm^3^)	GES	Pattern
Hb	Left iFG	53.8	−19.4	8.3	1,488	.030	HbR > HbO
	Left AG	43.9	66.1	37.5	920	.031	HbR > HbO
	Right iFG	−57.5	−11.9	8.0	792	.026	HbR > HbO
Hb × Cond	Left iPL	29.1	76.1	44.7	2,648	.025	C > N
							I > N
	Right sPL	−26.9	62.5	60.1	1,216	.016	N > C
	Left iPL	42.8	56.5	48.2	560	.022	I > N
Hb × Age	Left sTG	62.2	21.1	8.1	896	.026	30 > 54 months
Hb × Cond × Age	Right RO	−60.9	4.2	9.0	768	.015	30 months: C > I
							30 months: N > I

*Note*. AG = angular gyrus; HbO = oxygenated hemoglobin; HbR = deoxygenated hemoglobin; iFG = inferior frontal gyrus; iPL = inferior parietal lobule; mOG = middle occipital gyrus; RO = rolandic operculum; sPL = superior parietal; sTG = superior temporal gyrus.

**Figure 28:**
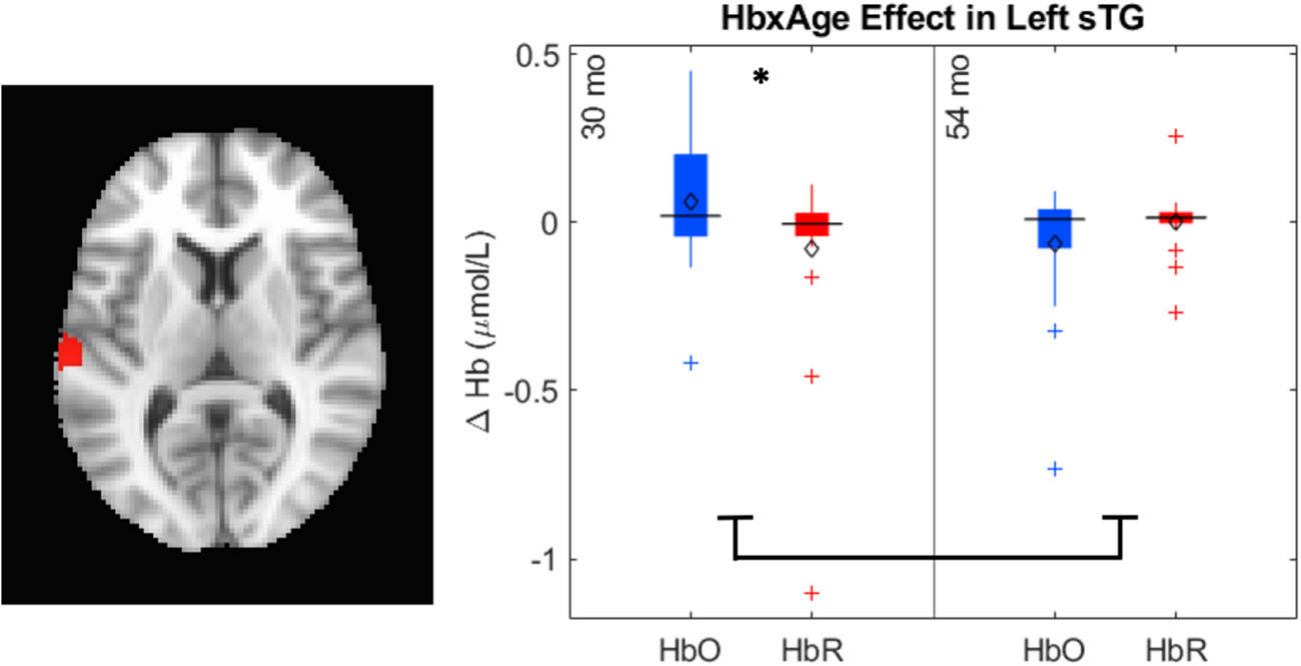
Cluster of neural activation in left superior temporal gyrus in which an interaction between oxygenation and age was observed such that this region was more active at 30 months compared to 54 months.

We also observed a significant age × Hb interaction in left superior temporal gyrus (sTG) (Figure 28) where activation was stronger at 30 months than at 54 months (*p* = .013). This region is typically implicated in language and auditory processing (Leff et al., 2009; Vander Ghinst et al., 2016), and specifically left superior temporal gyrus also contains Wernicke's area, a brain region involved in speech processing (Ardila et al., 2016b; Bhaya‐Grossman & Chang, 2022; Graves et al., 2008; Yi et al., 2019). Greater activation at 30 months could be because children required more verbal prompting from the experimenter to stay on task at 30 months compared to 54 months of age. Thus, this effect may be less due to the actual processing of the stimuli in the task and more to the research environment at large.

**Figure 29:**
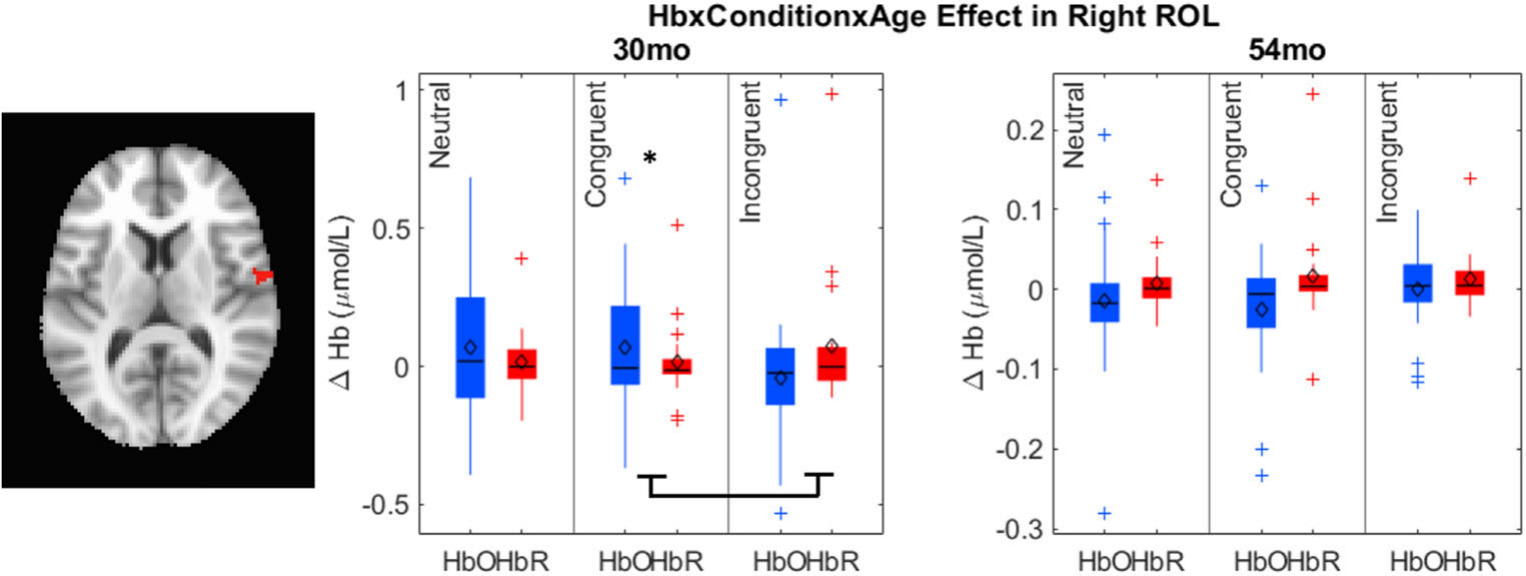
Cluster of neural activation in right Rolandic operculum displaying an interaction between oxygenation, condition, and age where 30‐month neutral trials showed significantly stronger activation than 30‐month incongruent trials.

Lastly, we found an interaction between Hb, condition, and age in right rolandic operculum (RO) (Figure 29). At 30 months, activation was stronger on congruent (*p* = .022) and neutral (*p* = .026) trials when compared to incongruent trials. Rolandic operculum in the left hemisphere is caudally adjacent to Broca's area (Perrone‐Bertolotti et al., 2020). Increased activation in this region at 30 months of age may reflect additional resources for labeling the target stimulus needed for response selection and this may decrease developmentally due to increased lateralization of language functions.

### Conclusion

This is the first examination of functional neural activation during a response selection task this early in development. At 30 months, the children in our study showed a pattern of response that suggested they were very strongly influenced by the spatial location of the stimulus. In terms of reaction time, children were fastest on congruent trials, suggesting that the spatial location of the stimulus facilitated the execution of a response. In contrast, 30‐month‐olds were most error prone on incongruent trials, showing that they, as expected, impulsively responded based on the location of the stimulus rather than suppressing attention to this aspect of the stimulus. At 54 months, however, there were no differences in performance across conditions. Reaction time did not differ between conditions, and accuracy was only marginally higher (.992 ms) for congruent trials than incongruent (.946 ms) or neutral (.945 ms) trials.

Interestingly, all developmental changes in neural activation that we found were associated with reductions in activation strength across age. Specifically, we observed reductions in activation in left superior temporal gyrus and that 30‐month‐olds showed increased activation in right Rolandic operculum on congruent and neutral trials. These findings may generally indicate that the brain becomes more efficient at recruiting task relevant regions or that more neural resources are needed to support performance on the task earlier in development. Consistent with previous literature (Kerns, 2006), we observed that inferior parietal cortex responded to incongruent trials, likely responding to the demands to suppress the task‐irrelevant spatial location of the stimulus. Other aspects of parietal cortex showed responses that were specific to other task conditions. Together, our results shed light on emerging neural mechanisms of response selection and inhibition of task‐irrelevant information.

## VII Predicting EF at 54‐Months With Measures of Neurocognitive Function

In this chapter, we examine which measures of neurocognitive function across the three domains examined in Chapters IV through VI (dimensional label learning, dimensional understanding, and simple EF) predict our target measure of EF on the DCCS task. The DCCS task is widely regarded as a standard measure of the developmental status of EF. The version we administered was modeled from the NIH Toolbox (Zelazo et al., 2013). We computed a continuous score of performance rather than the typical binary pass/fail measure from this task. This scoring system takes into consideration accuracy, reaction time, and previous sorting history to give a more holistic performance review than just accuracy alone.

As a follow‐up to these analyses, we also examined which measures of neurocognitive function across these three domains predicted performance on the Simon task in our cohort at 54 months of age. The Simon task does not yield a single metric of performance since three different conditions were administered (neutral, congruent, and incongruent) to each participant. To characterize performance on this task at 54 months of age, we chose to use reaction time on incongruent trials. Accuracy on this task was near ceiling, so meaningful variation was not likely to be found in terms of accuracy. The incongruent trials were slowest at 54 months of age and this slow down reflects participants taking the time needed to inhibit the automatic processing of the task‐irrelevant spatial location of the stimulus to instead respond based on the stimulus identity.

We treated each effect from the task‐based analyses above as a separate test on whether activation predicted our target behavioral outcome measures. For example, the Hb × dimension × age effect from the dimensional labeling tasks contained 12 measures of activation (computed as HbO minus HbR), four each (activation during 30 months color, 30 months shape, 54 months color, 54 months shape) from 3 clusters of activation showing this effect. These 12 measures were entered into a forward‐selection regression model to identify whether measures of activation from these clusters were meaningfully related to our target outcome measure from the DCCS task. Forward selection was used so that we could identify the best predictor of these outcomes and ensure that unique variance in outcomes were being accounted for if multiple predictors were selected.

### Methods

The DCCS task we administered was modeled off the NIH Toolbox version (Zelazo et al., 2013). This version of the DCCS was structured so that the children in our study made their responses by tapping on a touchscreen monitor. Children completed three phases: a pre‐switch, a post‐switch, and a mixed block. In the pre‐switch phase, children were instructed to sort by one dimension. For example, children may be told to sort by color first. Two cards were shown at the bottom of the screen: a yellow fish to the left and a purple house to the right. A card then appeared in the center of the screen which could be either a yellow house or a purple fish. Note that the dimensions of the test cards did not match the dimensions of the sorting cards. The experimenter demonstrated two trials to the child while giving the instructions, “This is the color game. In the color game we put yellow ones here (point to the yellow fish card at the bottom left) and purple ones here (point to the purple house card at the bottom right). I'll do the first two for you. This one is yellow so it goes here (touch the yellow fish card) and this one is purple so it goes here (touch the purple house card). Are you ready to play?” If children sorted incorrectly, they were reminded of the rules of the color game.

After five trials of the pre‐switch phase, children moved onto the post‐switch phase where they sorted based on the other dimension. In this example, children sorted based on shape. The sorting cards at the bottom of the screen did not change, and the experimenter did not demonstrate any trials. Instead, they verbally prompted, “Now we're going to play the shape game. In the shape game, fish go here (point to the yellow fish at the bottom left) and houses go here (point to the purple house at the bottom right).” If children sorted incorrectly, they were reminded of the correct dimension with the prompt, “remember we're playing the shape game. In the shape game, fish go here and houses go here.”

**Figure 30:**
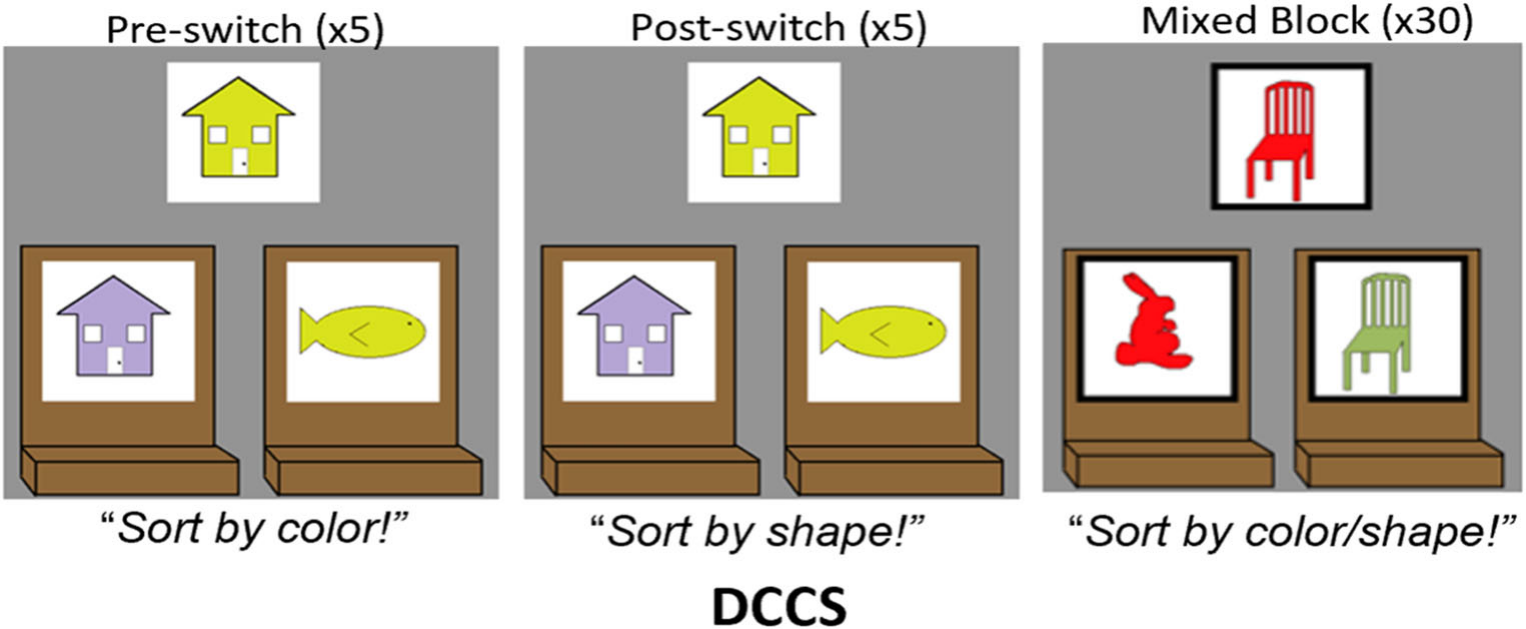
Phases of the dimensional change card sort task. Participants receive five consecutive trials sorting by the pre‐switch dimension, in this example color. After these trials, participants will receive five trials post‐switch sorting by the post‐switch dimension, in this example shape. Note that the placement of the cards do not change, resulting in a sorting conflict where children are sorting the same cards to opposite spatial locations between the pre‐ and post‐switch phases. Next, the child completes 30 randomized mixed block trials. 20 of these trials are in the post‐switch dimension, and the other 10 are pre‐switch.

After five post‐switch trials, children moved onto the mixed‐block phase. In this, phase children sorted based off of color or shape and were instructed verbally which dimension to sort by at the beginning of each trial. For the mixed block, the sorting cards switched to a red bunny on the bottom left and a green chair on the bottom right. The test cards were a red chair and a green bunny. Children were prompted, “Now sometimes we're going to play the color game and sometimes we're going to play the shape game. In the color game red ones go here (point to the red bunny in the bottom left) and green ones go here (point to the green chair in the bottom right). In the shape game bunnies go here (point again to the red bunny) and chairs go here (point to the green chair). I'll tell you which one to play.” If the child sorted incorrectly, they were reminded of both rules with the prompt, “Remember. Sometimes we're going to play the color game. In the color game red ones go here (point to the red bunny in the bottom left) and green ones go here (point to the green chair in the bottom right). Sometimes we're going to play the shape game. In the shape game, bunnies go here (point to the red bunny in the bottom left) and chairs go here (point to the green chair in the bottom right).” Children completed 30 randomly permutated mixed‐block trials, with 20 trials instructing children to sort by the post‐switch dimension and 10 trials instructing them to sort by the pre‐switch dimension. Figure 30 displays the methods of the NIH toolbox version of the DCCS.

### Predicting DCCS Performance

**Figure 31:**
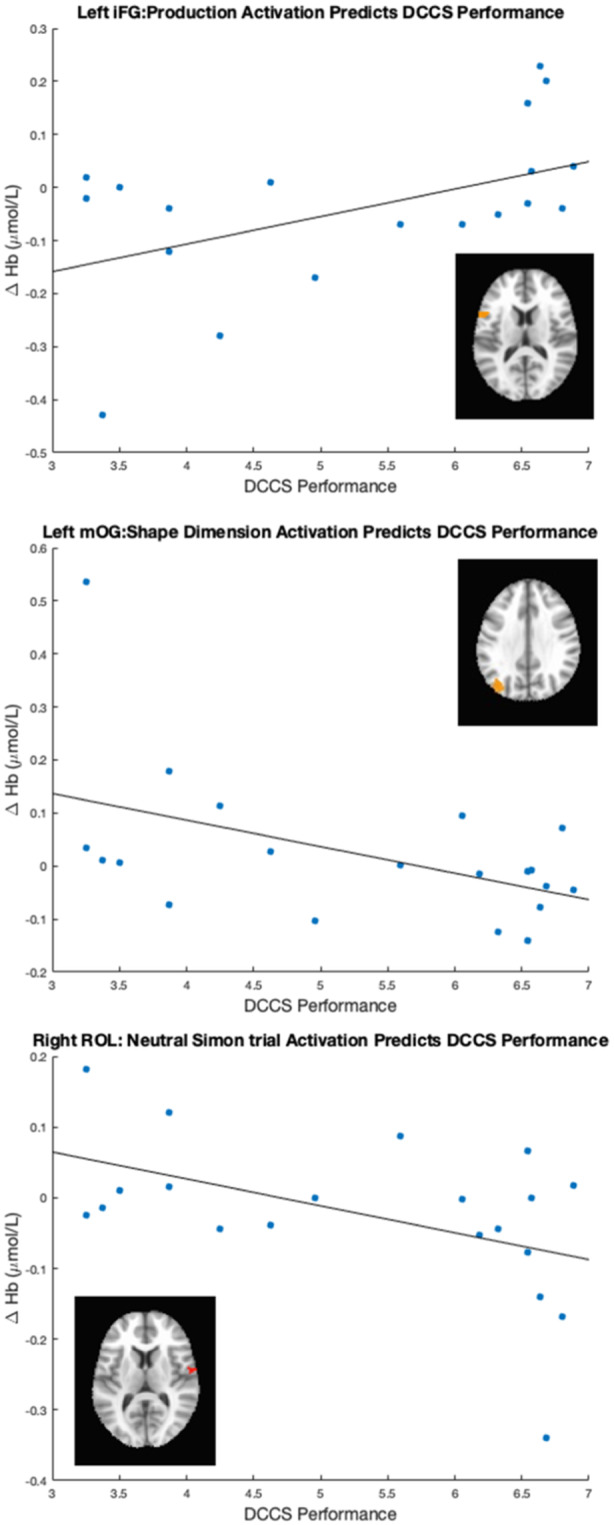
Correlations depicting the relationship between neural activation (*Y* axis) and performance on the DCCS task (*X* axis) using the neural activation clusters found in earlier analyses. Activation in left inferior frontal gyrus from a dimensional labeling Hb × task × age effect during production tasks predicts better DCCS performance (left), activation of left middle occipital gyrus from a dimensional labeling Hb × dimension effect during shape dimension tasks predicts worse DCCS performance (right).

Table 5 and Figure 31 show the measures of neurocognitive function that predicted performance on the DCCS task. First, we examined patterns of activation in relation to the dimensional label learning tasks. Activation during the production tasks (coming from the age × task × Hb interaction) in left iFG were positively associated with performance on the DCCS task, *F*(1,17) = 6.149, *p* = .024, *R* ^2^ = .266. Thus, the more strongly children in our study activated this region during production at 30 months of age, the better they did on the DCCS at 54 months of age. The task‐based analysis of activation from this cluster showed greater activation during the production task relative to the comprehension task at 54 months of age. Greater activation during the production task at 30 months of age suggested that children were showing more developmentally advanced activation in this region. These findings replicated those from one of our previous studies examining the association between dimensional label learning at 33 months of age and DCCS performance at 45 months of age. Lowery et al. (2022) reported that activation in left middle frontal gyrus during dimensional label production predicted performance on the DCCS task. This cluster is near that reported in the current study. Additionally, activation during shape tasks (coming from the dimension × Hb interaction) in left mOG was negatively associated with DCCS performance, *F*(1,16) = 6.769, *p* = .019, *R* ^2^ = .297. Thus, weaker activation in this region during shape tasks was associated with better performance on the DCCS task. Task‐based analyses in this cluster showed greater activation for shape compared to color, which we interpreted as reflecting greater engagement of lower‐level stimulus processing for shape. Thus, weaker activation during shape trials could reflect a shift to higher level processing of shape information. In this case, we would expect weaker engagement of this region during shape tasks to be associated with processing shapes at a higher‐level of categorization, and thus, better attention skills and better performance on the DCCS task. This interpretation is in line with the fact that shape is often the most salient feature for categorization.

**Table 5:** Measures of Neural Activation That Predicted Dimensional Change Card Sort (DCCS) Performance

*Neural Regions of Activation Found to Predict DCCS Performance*						
Task	Effect	Predictor	Region	*β*	*p*	Adj *R* ^2^
DLL	Age × Task × Hb	30 months production	Left iFG	.515	.024	.222
	Dim × Hb	Shape	Left mOG	−.545	.019	.253
Simon	Age × Cond × Hb	54 months neutral	Right RO	−.482	.031	.190

*Note*. DLL = dimensional label learning; iFG = inferior frontal gyrus; mOG = middle occipital gyrus; RO = rolandic operculum.

In relation to patterns of activation from the Simon task, activation in right Rolandic operculum (coming from the condition × age × Hb interaction) predicted DCCS performance. Specifically, weaker activation in this region during neutral Simon trials at 54 months was associated with better performance on the DCCS, *F*(1,18) = 5.447, *p* = .031, *R* ^2^ = .190. Task‐based analyses of activation in this region showed that activation was stronger on neutral and congruent trials relative to incongruent trials at 30 months of age. Thus, these analyses suggested that activation in this region declines with age, and the decline in activation in this region is associated with better EF.

Examinations of clusters of activation from the Matching task yielded no significant associations with performance on the DCCS task.

### Predicting Simon Performance

**Figure 32:**
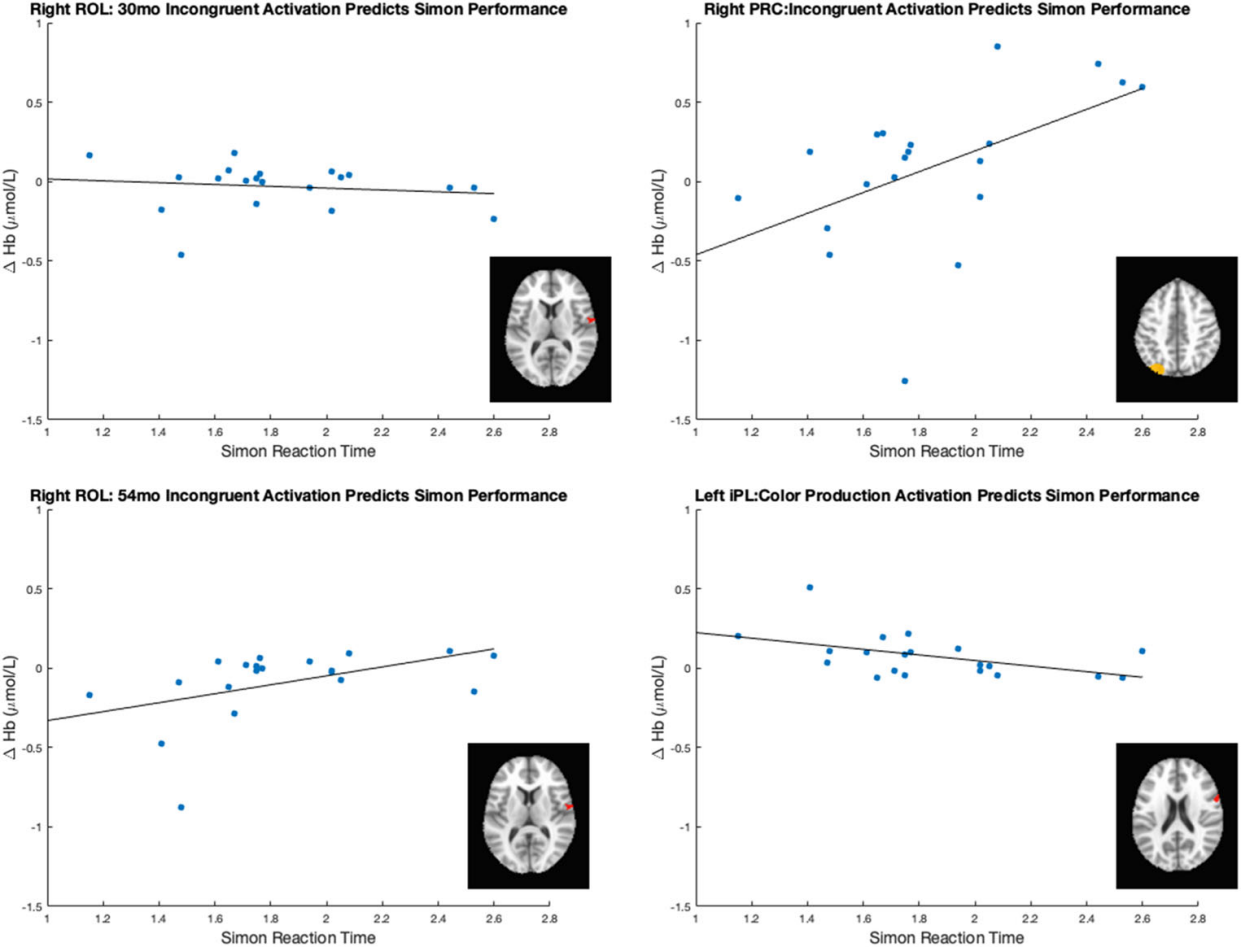
Correlations depicting the relationship between neural activation (*Y* axis) and Simon reaction time at 54 months (*X* axis) using the neural activation clusters found in earlier analyses. Right Rolandic operculum activation from a Simon task Hb × condition × age effect during incongruent trials at 30 months predicts slower Simon reaction times (top left), right precentral gyrus activation from a Simon task Hb × condition effect during incongruent Simon trials predicts faster Simon reaction times (top right), right Rolandic operculum activation from a Simon task Hb × condition × age effect during incongruent trials at 54 months predicts faster Simon reaction times (bottom left), and left inferior parietal lobule activation from a dimensional labeling Hb × dimension effect during color production dimensional labeling trials predicts slower Simon reaction times.

Table 6 and Figure 32 show the measures of neurocognitive function that predicted performance on the Simon task. As a reminder, we used reaction time during Incongruent trials at 54 months of age as an index of performance on the Simon task. First, activation during incongruent trials at 30 (coming from the age × condition × Hb effect) in right Rolandic operculum were associated with Simon task performance, *F*(1,18) = 10.132, *p* = .005, *R* ^2^ = .325. Greater activation in this region at 30 months of age was associated with slower reaction time at 54 months of age, but greater activation in this region at 54 months of age was associated with faster reaction time at 54 months of age. Task‐related activation in this region showed greater activation on congruent trials relative to incongruent trials at 30 months of age. To further explore the brain‐behavior relationship in the context of these contradictory associations between neural activation at different ages and Simon task performance, we computed the change in activation on incongruent trials in this cluster by subtracting activation at 30 months from activation at 54 months. As shown in Figure 32, the amount of change in activation was associated with Simon performance. Specifically, larger increases in activation on incongruent trials was associated with faster RTs on incongruent trials at 54 months of age, *r*(18) = −.572, *p* = .008. Thus, although task‐based analyses of neural activation did not indicate activation in this region on incongruent trials, these results suggest that this region is developmentally increasing in response to task demands on incongruent trials.

**Table 6:** Measures of Neural Activation That Predicted Simon Performance

*Neural Regions of Activation Found to Predict Simon Task Performance*						
	Effect	Predictor	Region	*β*	*p*	*R* ^2^
Simon	Age × Condition × Hb	30 months incongruent	Right RO	.600	.005	.325
	Condition × Hb	Incongruent	Left iPL	.491	.028	.199
DL	Dim × Task × Hb	Color production	Right PCG	−.491	.033	.196

*Note*. iFG = inferior frontal gyrus; iPL = inferior parietal lobule; PCG = precentral gyrus; RO = rolandic operculum.

Second, activation during incongruent trials (coming from the condition × Hb interaction) in left iPL was associated with performance on the Simon task, *F*(1,17) = 7.568, *p* = .015, *R* ^2^ = .335. Greater activation in this region was associated with slower reaction time at 54 months of age. Task‐based activation in this region showed that activation was greater on incongruent trials relative to neutral trials. Thus, activation in this region was specific to the presence of task‐irrelevant spatial information. Activation in this region, then, reflects the amount of distraction due to this task irrelevant information.

We also found that activation in the dimensional labeling tasks was associated with our target outcome measure of performance on the Simon task. Specifically, activation during color production at 54 months (coming from the dimension × task × age × Hb effect from the analyses of the dimensional label learning tasks) was associated with performance on the Simon task, *F*(1,17) = 5.386, *p* = .033, *R* ^2^ = .196. Greater activation during color production at 54 months of age was associated with faster reaction times on incongruent trials of the Simon task at 54 months of age. Task‐based analysis of activation in this region showed activation was greatest during color production. Thus, activation in this region reflected not only the quality of representations used in the color production task, but also reflected inhibitory control abilities with greater activation during color production being associated with more efficient suppression of task irrelevant information in the Simon task.

### Conclusion

Our results supported our primary predictions: performance on the DCCS task was predicted by neurocognitive measures of dimensional label comprehension and production. First, left iFG showed developmental increases in activation on the production task. Remarkably, stronger activation in this region during the production tasks at 30 months of age was associated with better performance on the DDCS task at 54 months of age. Thus, not only did activation in this region increase developmentally in the context of the dimensional label production tasks, activation in this region was also predictive of performance on our target EF task. Second, the degree of engagement in low‐level perceptual areas in mOG during shape comprehension and production trials was also associated with the development of EF. The weaker that this low‐level perceptual area was engaged, the better children performed on the DCCS task. To the extent that the children in our study relied less on lower‐level processing of shape information on shape comprehension and production trials, the better they did on the DCCS task.

Importantly, performance on the DCCS was not well predicted by measures of neurocognitive function from the Simon task. Based on traditional approaches to the study of EF, it would be expected that performance on the DCCS task would be predicted by neurocognitive measures from a simpler task that required similar components of EF, such as rule‐use or response selection. The Simon task is an ideal candidate in this regard because it requires children to follow a set of arbitrary stimulus‐response mappings but without the additional stimulus conflict and switching demands that make the DCCS task too difficult for children younger than 3 to attempt. From the Simon task, weaker activation in right Rolandic operculum during neutral trials at 54 months of age was associated with better performance on the DCCS task. The specific functions associated with this region are not clear from previous research; however, it is a homologue of Broca's area in the right hemisphere. During the Simon task, this region showed activation specifically during neutral and congruent trials, suggesting this region is engaged for basic stimulus categorization. Moreover, activation decreased from 30 to 54 months of age. Thus, we interpreted this region as showing a decrease in activity due to the lateralization of language regions with age. Framed in this way, this reduction in activation appears to be a developmental consequence of more general developmental phenomena that are unrelated to the specific demands on the Simon task. Indeed, when examining changes in Simon task performance, the best predictors were activation during incongruent trials which encapsulate the key challenges imposed by this simpler EF task. Together, these findings support our prediction that to better understand EF development, we need to look beyond traditional measures of EF to other aspects of perception/action development to understand the processes that give rise to EF.

Examining aspects of neurocognitive function that predicted performance on the Simon task, we observed that different measures of neural function during incongruent trials predicted reaction time on incongruent trials at 54 months of age. This is unsurprising since the neural and behavioral measures come from the same type of trial. In particular, these patterns of activation showed that greater activation was associated with slower reaction time on incongruent trials, indicating that these regions reflect the degree of distraction due to the task‐irrelevant spatial location of the stimulus. More surprisingly, we found that neurocognitive function in right PCG during color production trials predicted reaction time on incongruent trials. Thus, children's ability to label visual feature dimensions impacted performance on other tasks that do not involve feature labels in the way the DCCS task does.

Together, these results highlight a new insight on EF development. Whereas previous research has focused on other EF measures to predict future EF or other developmental outcomes, the current study looked beyond EF to identify whether other processes could explain the development of EF. We found that measures of neurocognitive function during dimensional label learning tasks predicted the future development of EF. This finding is noteworthy because it suggests a tangible learning process contributes to changes in EF. Previous research on EF interventions have administered EF tasks for participants to practice. This strategy treats EF like a muscle that would be exercised by training or intervention protocols. Although participants would improve on the trained tasks, this training rarely generalized to other EFs or other developmental outcomes. The findings reported here motivate an alternative approach that instead focuses on training associations between perception‐action systems. In this case, forming associations between labels and visual features provides neural mechanisms by which children can guide internal processing of visual information. We return to the issue of the nature of EF and interventions in the final chapter.

## VIII General Discussion

### Summary of the Current Project

The research project described in this monograph was motivated by a DF model that has been used to explain a wide range of behavioral and neural effects in the literature on the benchmark measure of EF that we used, the DCCS task. A novel aspect of this model that is central to its explanation of EF development, is that verbal labels are used to enhance processing of visual information. This enhanced processing gives rise to a form of dimensional attention that can boost activation within visual dimensions such as shape or color. The central prediction of the model is that changes in EF are driven by changes in label learning. In the case of the DCCS task, this specifically involves labels for visual dimensions (e.g., “shape” or “color”).

Previous work has developed this computational framework to explain existing behavioral and neural data from the DCCS task as well as make empirically supported predictions about behavior and neural data from the DCCS task. Although previous data has supported the role of dimensional label learning on EF development, prior research has not rigorously tested this relationship. In the current project, we directly probed the relationship between dimensional label learning and EF outcomes using a sample of children that was followed longitudinally from 30 to 54 months of age. To test the role of dimensional label learning in predicting EF, we measured neurocognitive function across multiple domains at 30 months of age. We focused on domains that have been previously implicated in development of performance on the DCCS task, namely simple EF and dimensional understanding. We first identified the cortical regions that were associated with performing each task and regions that changed in activation over development within these domains. We then determined which of these measures of neurocognitive function were associated with EF outcomes on the DCCS task at 54 months of age. The best predictor of DCCS performance was neural activation in left inferior frontal cortex during label production at 30 months of age. The more strongly children activated this region during dimensional label production at 30 months of age, the better they did on the DCCS task at 54 months of age. Importantly, this region of inferior frontal cortex also showed developmental increases in activation between 30 and 54 months during the production tasks, suggesting that this region is changing in function in relation to the dimensional label tasks and these changes are associated with EF outcomes. It is also important to note that this central finding replicates a previous report that activation in left frontal cortex during dimensional label production at 33 months of age was associated with DCCS performance at 45 months of age (Lowery et al., 2022).

### Rethinking EF Development

The DF model offers a unique perspective on the nature of EF. EF is a property of the model, not the result of any single component in the model. Moreover, there are no components in the model that are inherently for EF. In this way, the model motivates looking beyond measures of EF to identify the mechanisms that give rise to EF. The model provides a computational framework in which dimensional label learning plays a central role in the emergence of cognitive control skills. In particular, labels serve as a way of organizing thought and cognition around perceptual dimensions. This organization is not task, context, or EF specific. Rather, these associations are task general and confer a means of structuring cognition in both EF tasks and non‐EF tasks (Buss & Kerr‐German, 2019). This is the only framework to state explicitly the mechanisms that give rise to EF. Incremental changes in the strength of associations between labels and visual features give way to new cognitive and attentional skills. In this way, the model is built around perception/action dimensions involved with forming representations of objects and multi‐modal associative learning that links labels with visual feature dimensions. Together, this system displays autonomous rule‐following behaviors, attention to visual dimensions, and ultimately, cognitive flexibility on the DCCS task.

In this monograph, we focused on the application of a DF model to the DCCS. However, the application of DF models is not limited to this particular EF task. Other models have been applied to performance of young adults on a wide range of tasks. Erlhagen and Schöner (2002) used a simple DF model to demonstrate how various canonical EF effects arise from neural population dynamics. They discussed the Simon effect, which would be relevant to the current project. In this work, differences in reaction time based on condition were a result of lateral inhibition slowing down a response peak when the spatial location of the stimulus conflicted with the response location associated with the stimulus. Future work can probe the neural mechanisms responsible for changes in the Simon effect over development. In an expanded DF model that we discuss below, we would be able to implement the full suite of tasks that we administered in the work described in this monograph. Recall, 30‐month‐olds were heavily influenced by the object location information, showing the greatest error rate on incongruent trials. Older children, however, showed a more canonical effect of slower reaction times on incongruent trials, suggesting they were engaging EF processes to suppress the task‐irrelevant spatial location information. Such effects may result from a label learning process that we have discussed in the context of the DCCS task. For example, associating labels for the dog and cat category may serve to enhance processing of object identity above and beyond stimulus location. Effects in the Simon task may also be due to developmental changes in neural tuning that increase the strength of excitation and inhibition within neural populations. Referred to as the spatial precision hypothesis, such a mechanism has been used to explain changes in visual working memory and spatial working memory (Schutte & Spencer, 2009; Simmering, 2016; Simmering & Patterson, 2012).

The conceptual framework offered by the DF model also lends to thinking about individual differences and the influence of context and culture, which has been a focus of recent research (Doebel, 2020; Gaskins & Alcalá, 2023). A key aspect of this new perspective is the centrality of autonomy to behavior. The autonomous behavior of the model is influenced by a range of factors, such as the biological properties governing the strength of excitation and inhibition within neural populations (e.g., Schutte et al., 2003; Simmering & Patterson, 2012), the salience of stimuli (e.g., Diedrich et al., 2001; Thelen et al., 2001), the structure of a task (Buss & Kerr‐German, 2019; Buss & Spencer, 2014), and the specific history of learning that has shaped the patterns of connectivity between neural populations in the model (akin to its conceptual structure; Bhat et al., 2022). This connection is particularly important because it provides a platform to explore how the amount and quality of label input and variability in exposure to objects impacts label learning and the subsequent impact of labels on attention and measures of EF. Moreover, this platform can also be used to explore how unique aspects of language use across cultures might foster different patterns of learning that can differentially impact attention and EF. Other factors such as motivation, engagement, or arousal can be linked to dynamic changes in resting levels within populations of neurons which can influence the overall amount of energy needed to engage a population, the relative stability of activation within a population, or the amount of output from a population. Thus, framing EF as a property of a neurocognitive system that produces autonomous behavior provides a general conceptual framework that can not only reconcile the domain general and context specific aspects of EF, but is also flexible enough to explore how EF emerges in the context of different learning opportunities.

### EF Training, Interventions, and Individual Differences: Looking Beyond EF

The most important insight provided by this neurocomputational framework is that dimensional label learning provides a mechanism by which EF develops. To assess this prediction, we conducted a longitudinal study that assessed an unprecedented breadth of neurocognitive function in early childhood. Our goal was to explore whether neurocognitive functions involved in dimensional label comprehension and production predicted success on measures of EF better than other domains of neurocognitive function that have been implicated in the literature. In this regard, we compared the ability of dimensional label learning measures to predict EF against measures of simple EF as assessed in the Simon task and measures of dimensional understanding tapped into by our matching tasks. The only domain that predicted success on the DCCS task was dimensional label learning. Our results also replicated previously reported findings from a smaller scale longitudinal study (Lowery et al., 2022). Together, these findings suggest that neural activity during dimensional label production in left frontal cortex is predictive of future performance on the DCCS task.

### A Learning‐Based Approach to EF Development

These results suggest that new strategies can be devised for training EF in early childhood. Specifically, training dimensional labels should confer benefits on developmental trajectories of EF. This type of intervention is distinct from the intervention methods previously explored in the literature. For example, work from the CCC theory by Zelazo and colleagues (Espinet et al., 2013) have shown that prompting children to stop and reflect on the nature of a task can alter children's performance and neural indices of cognitive conflict. Other programs such as CogMed (Pearson Education; www.cogmed.com) and school‐based curricula such as Tools of the Mind (Bodrova & Leong, 2018) have had some impacts on trained domains, but inconsistent evidence of generalization and transfer (for reviews, see Diamond & Lee, 2011; Niebaum & Munakata, 2023). The challenge with these other approaches is relative ambiguity of the mechanisms engaged by the interventions. For example, in reflection training, research shows that having children take time to reflect on the nature of a task improves performance on EF tasks. What is not clear, however, is whether the effect of reflection changes developmentally, what mechanisms mediate the relative impact of reflection on performance, or how learning and experiences shape the cognitive systems involved in task performance.

### Limitations and Future Directions

One of the most important limitations of this study is the homogeneity of our participant sample. All our participants were White and from upper/middle class households. Part of the challenge in this regard are the constraints on conducting lab‐based research that requires multiple hours of data collection per year to assess the broad range of neurocognitive functions that we assessed here. For most families, especially those from economically disadvantaged backgrounds, having the time and resources to participate in such research is not possible. One way of addressing this challenge is with portable neuroimaging. Recent work highlights the ability to not only take neuroscience into the classroom, daycare, or home (McKay et al., 2021), but also abroad to study neurocognitive development in some of the most challenging circumstances (Wijeakumar et al., 2023). Such efforts may yield valuable insights into neurocognitive development that would not be available using traditional convenience sampling. The unfortunate reality is that research using a convenience sample of children has been dominated by samples from White middle‐class families.

Recent work highlights the need to take culturally, ethnically, and socially diverse approaches to understand the role of context on brain development. First, a large community‐sample of children showed that associations between age and brain structure are not consistent across SES, race/ethnicity, or sex, suggesting that largely homogenous convenience samples will not yield generalizable results (LeWinn et al., 2017). fNIRS presents unique challenges in this regard because research has demonstrated that current methods are unable to ensure that all racial and ethnic groups can provide data. fNIRS relies upon the measurement of near‐infrared light and measures of fNIRS can be impacted by melanin levels and hair type, leading data from participants with darker skin tones or coarser hair to be discarded and unusable (Webb et al., 2022). This issue is compounded by the fact that reporting racial and ethnicity information is not standard practice in fNIRS research (Kwasa et al., 2023). Thus, efforts need to be focused on developing the technology and data processing methods to ensure high quality fNIRS data can be collected from all individuals. Second, studies of EF in particular have shown that performance on EF tasks can be influenced by a wide range of factors related to the cultural relevance of the task. For example, the logic or demands imposed by a task may be unrelated to the typical sources of motivation, the structure of social interactions, or the beliefs held across cultures (Gaskins & Alcalá, 2023). Thus, by recruiting more representative samples of participants, we can also develop measures that more accurately reflect the construct of EF.

It is of vital importance to address these limitations with future research so that we can more broadly understand the nature of cognitive function and the dynamics driving brain development. Possible strategies for obtaining more inclusive and diverse samples of participants include ensuring a diverse team of researchers engage in community‐based recruitment and targeting the specific communities of interest. It is also essential to establish a relationship with the local community and to communicate the importance of research. Even more important, research activities should directly benefit the communities, either through sharing of information about research findings or by translating research findings into actions that can benefit these communities. In any case, future research must make serious efforts to make the sample of families in research more representative of the underlying population. Doing so can help draw more accurate conclusions from our studies that will allow our research findings to be translated into actions or programs that are beneficial to all children's development. As reviewed in Chapter I, culture and SES impact performance on tasks assessing EF (Assari, 2020; Howard et al., 2020). However, it is not clear how much of the discrepancy we see in developmental studies arise from actual developmental differences versus artifacts of measurement due to lack of sensitivity to language or culture. For example, African American children on average perform less well on the Peabody Picture Vocabulary Test than their White peers not because they are behind vocabulary development but because they are simply less likely to be exposed to the words in the test (Champion et al., 2003).

In relation to the issues regarding generalizability, we also recognize that a limitation of our study is the small (*n* = 20) sample size. Although the pandemic had a negative impact on our ability to follow all of the children initially recruited into the study, we found no significant differences between children who completed both years of data collection and those who completed only the first year in terms of behavioral and neural measures of EF, suggesting that performance on EF tasks was unrelated to attrition. It is also important to note, however, that within each participant we obtained a tremendous amount of longitudinal data on neurocognitive function and our study design assessed predictions within children. In the longitudinal assessments, the child's scores on EF assessments were compared to earlier measures of neurocognitive function. In this way, we measured individual development. Future work should address whether these relationships between early neurocognitive function and later EF outcomes are also present within groups that are more representative of the general population and whether these aspects of early neurocognitive function predict EF outcomes cross‐culturally. The basic premise of the DF model that motivated this research is that multimodal associative learning between auditory labels and visual dimensions confers cognitive control over processing of visual information. This process will inherently be influenced by how language emphasizes types of information (Gibson et al., 2017). Even within cultures, there are likely differences in the ways that children are exposed to language (Hoff & Tian, 2005). Future work using more representative samples or comparing across cultures and across languages can explore how the structure and use of language shape how cognitive control develops.

Another strength of our findings as they relate to eventual intervention programs is the simplicity of the mechanism of growth we describe. This framework predicts that exposure to color and shape labels will facilitate better understanding of the feature dimensions and, consequently, cognitive control. The specific details of how objects and labels are presented to children to optimize this learning remains to be discovered, but this process is expected to be general and to facilitate development regardless of children's developmental status in early childhood. Although this work does not speak to identifying children “at‐risk” or children that would benefit to different degrees from intervention, we expect such intervention efforts to close the gap between developmental trajectories for children of different backgrounds.

Future work can leverage recent advancements in other applications of DF models. The work we have carried out in the context of EF development highlights the first and only learning‐based mechanisms of EF development. Interventions that target label learning can be cost‐effective and easily portable. Although this research highlights the role of dimensional label learning in EF development, the specific mechanisms guiding changes in neurocognitive function in relation to label learning remain to be identified. Other applications of a DF model have simulated the dynamics of cross‐situational word learning. This model architecture contains the same architecture of the model discussed in Chapter II with additional components that form associations between labels and visual features (Bhat et al., 2022). Thus, this model provides an ideal context to explore the types of experiences that are most effective in facilitating developmental change in relation to dimensional labels. Note, too, that this model can perform a wide range of tasks, including other EF tasks that we can use to probe developmental processes. We are currently working to implement the comprehension, production, Simon, matching, and DCCS tasks all within this single model architecture. Other advances with the DF model framework illustrate how real‐time neural responses can be simulated and rigorously tested against neural measures from human participants (Buss et al., 2020; Buss & Spencer, 2018). This provides the opportunity to test intervention protocols not only in terms of a target behavioral outcome, but also in terms of target neural responses. The results presented in this monograph illustrate the patterns of neural activation that should be targeted for optimizing this learning problem.

### Conclusion

The ultimate goal of developmental research is to put our understanding of development to use to improve developmental outcomes. EF presents an important topic for study due to the widespread influence it has on developmental outcomes in academics and general quality of life. The work presented here identifies a novel learning‐based mechanism that interventions can target with the goal of improving developmental outcomes. Of note is the simplicity of the learning mechanism suggested. Shape and color label learning at 30 months of age may lead to better EF abilities at 54 months of age. This finding provides ample opportunity for many possible interventions that are both inexpensive and easy to implement in a wide range of learning contexts, including by parents in the home and by teachers in formal educational settings. Our results suggest that forming associations between labels and aspects of the visual world builds mechanisms by which children can control attention to stimuli in their environment. For example, a strong knowledge of color labels will lead to a better understanding of the color dimension which in turn leads to successful performance on measures of EF. Of course, the real impact of this work will be revealed by future efforts to engage these learning processes to examine their generalized impact on developmental outcomes that impact the quality of lives of children. We are hopeful that the data provided here may point the way to facilitate broad aspects of developmental trajectories.
